# Fluorinated Solvents for Chemoselective Oxidations: A Strategy toward Synthetic Ideality in Natural Product Synthesis

**DOI:** 10.1021/acsorginorgau.5c00121

**Published:** 2026-01-23

**Authors:** Victor C. S. Santana, Lucas D. P. Gonçalves, Yasmin N. Salmazo, Julian C. S. Pavan, Deborah de A. Simoni, Vladimir C. G. Heleno, Emilio C. de Lucca

**Affiliations:** † Instituto de Química, 28132 Universidade Estadual de Campinas, 13083-970 Campinas, SP, Brazil; ‡ Núcleo de Pesquisa em Ciências Exatas e Tecnológicas, 92917 Universidade de Franca, 14404-600 Franca, SP, Brazil

**Keywords:** chemoselectivity, metal-catalysis, oxidation, synthetic ideality, fluorinated solvents, diterpenes, natural products

## Abstract

Late-stage catalytic oxidations of complex natural products have been shown to exhibit dramatically improved chemoselectivity through the crucial use of 1,1,1,3,3,3-hexafluoro-2-propanol (HFIP), even in the presence of oxidatively sensitive groups, such as hydroxyls and diols. This strategy reduced the need for protecting groups, effectively mimicking enzymatic pathways, and substantially enhancing synthetic ideality in the preparation of *ent*-beyerane and *ent*-kaurane metabolites.

## Graphical abstract

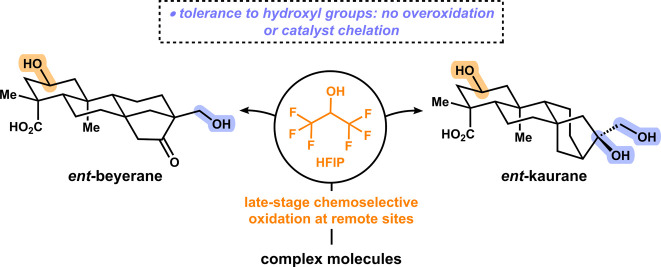

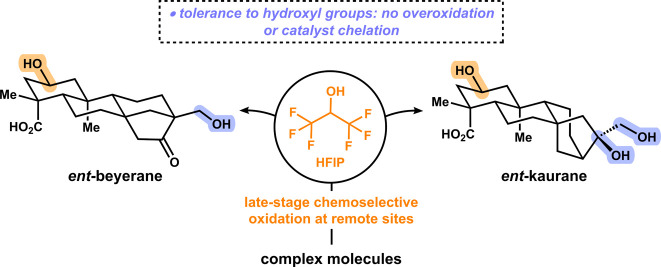

The increasing demand in the past decades for the invention of even more selective transformations has become critical, and chemoselectivity^1^ and synthetic ideality^2^ are now part of the lexicon of our scientific community. While the discovery of new reactions and methodologies using concepts such as step economy,^3^ redox economy,^4^ and the minimization of protecting group use^5^ has greatly expanded the chemistry toolkit,^6^ the use of solvents as key agents in achieving chemoselectivity remains underappreciated.

The choice of solvents in organic chemistry plays an essential role in the development of new reactions, as they not only are responsible for dissolving the species in the reaction medium, but also exert a significant influence on reactivity and selectivity.^7^

On the other hand, it was only recently that fluorinated solvents such as nonafluoro-*tert*-butyl alcohol (NFTBA), 1,1,1,3,3,3-hexafluoro-2-propanol (HFIP), and 2,2,2-trifluoroethanol (TFE), which exhibit noteworthy properties such as high polarity, high Brønsted acidity, low nucleophilicity, and strong hydrogen bond donating (HBD) ability,^8^ became widespread. These interesting features enable activation of hydrogen peroxide (H_2_O_2_) and organic functionalities,^9^ and have attracted attention for applications in a variety of organic reactions, including oxidations, ring openings, and cycloadditions.^10^

Fluorinated solvents were also applied for chemoselective C—H bond oxidations in hydroxyl-containing substrates (Scheme 1A).^11^ The activation of the carbinolic C—H bond via hyperconjugation with the adjacent oxygen atom can result in overoxidation products.^12^ However, hydrogen-bonding interactions promoted by HFIP and TFE have been shown to mitigate this issue by reversing the polarity of the substrate.^13^ This effect deactivates carbinolic C—H bonds, thereby enabling selective oxidation at remote sites. Compared with the sole use of acetonitrile (MeCN), fluorinated alcohols consistently afford higher selectivities and yields for hydroxylated products.

**Scheme 1:**
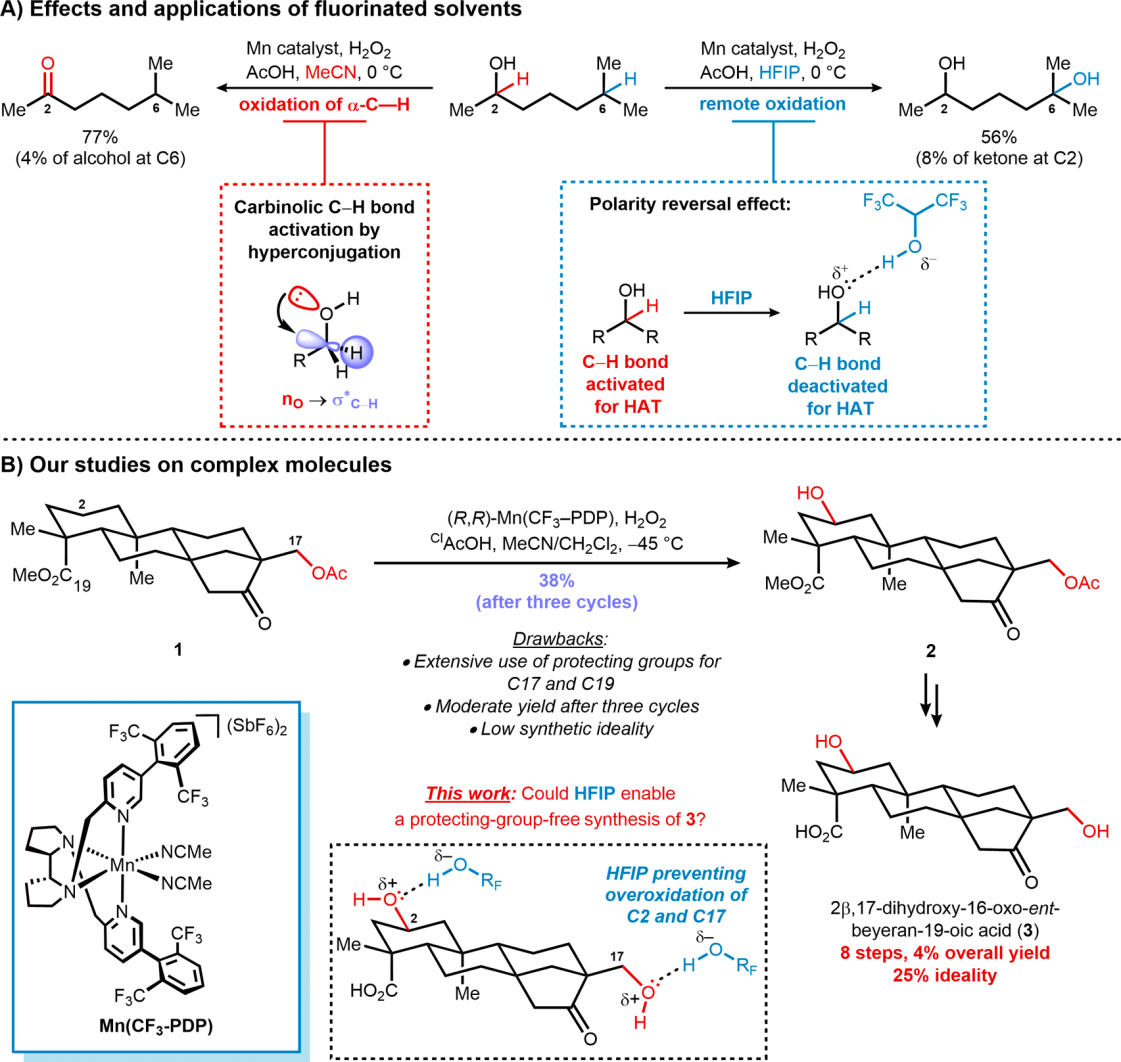
A) Effects and Applications of Fluorinated Solvents and B) Our Studies on Complex Molecules

While these beneficial effects are easily recognized in simple molecules, the lack of complex substrate examples in these studies tends to undermine their use in natural product synthesis. Such transformations, especially when applied at a late-stage,^14^ allow rapid access to molecules, expediting structural diversification and broadening the chemical space.^15^

By combining the deactivation of functional groups with the prevalent formation of alcohols, fluorinated solvents enhance the biomimetic character of metal-oxo species by modulating their reactivity to more closely resemble that of enzymatic systems, particularly the P450 superfamily.^16^ The ability of small molecules to imitate the selectivity of enzymes enables new reactivity patterns and more chemoselective transformations,^17^ affording synthetic routes in higher yields and fewer reaction steps by reducing or even eliminating the need for protecting groups.

We envisioned that the use of HFIP and TFE in late-stage, nondirected C—H oxidations could be challenged by complex diterpenes in the presence of White–Gormisky–Zhao catalyst Mn­(CF_3_–PDP) and H_2_O_2_ (Scheme 1B).^18^ When compared to our previous report,^19^ the use of fluorinated solvents could prevent overoxidation of the C2 position affording higher selectivities and yields for alcohol **2**. Moreover, the polarity reversal effect could assist in the tolerance for a preinstalled C17-hydroxy group, eliminating the need for protecting groups, which hampered our synthetic ideality in the first synthesis. These outcomes would facilitate a more chemoselective and optimal synthesis of *ent*-beyerane **3** and would further advance the investigation of *ent*-kaurane metabolite synthesis.

Initially, we investigated the chemoselective C—H bond oxidation of isosteviol methyl ester (**4**) to alcohol **5** employing HFIP and TFE along with CH_2_Cl_2_, and compared these results with the MeCN/CH_2_Cl_2_ media (Table 1).^20^ Using MeCN/CH_2_Cl_2_ 1:1 at 0 °C and 10 equiv of H_2_O_2_, we obtained 50% isolated yield in a ratio of 80:20 ketone/alcohol (Entry 1). When acetonitrile was replaced with TFE (TFE/CH_2_Cl_2_ 1:1), the yield was slightly improved, while the selectivity for ketone **6** remained the same (Entry 2). The use of HFIP/CH_2_Cl_2_ 1:1 led to a dramatic inversion in selectivity and the formation of alcohol **5** was favored (25:75, Entry 3) with a decrease in yield. Lowering the temperature to −36 °C increased the yield to 62% and the selectivity for alcohol remained the same (Entry 4). While decreasing the amount of H_2_O_2_ to 2 equiv proved unproductive (Entry 5), increasing the HFIP/CH_2_Cl_2_ ratio to 4:1 afforded alcohol **5** as the sole product in 61% yield (Entry 6). In these experiments, solely the starting material and products **5** and **6** were detected and successfully isolated.

**Table 1:**
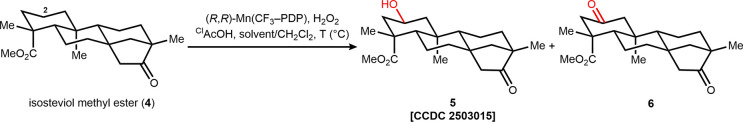
C—H Bond Oxidation of Compound **4** with (*R*,*R*)-Mn­(CF_3_–PDP)^a^

Entry	*T* (°C)	H_2_O_2_ (equiv)	Solvent/CH_2_Cl_2_ (ratio)	Total yield^b^	Selectivity (5:6)	RSM (%)
1	0	10	MeCN (1:1)	50	20:80	15
2	0	10	TFE (1:1)	56	22:78	0
3	0	10	HFIP (1:1)	49	75:25	0
4	–36	10	HFIP (1:1)	62	82:18	0
5	–36	2	HFIP (1:1)	58	83:17	26
**6**	**–36**	**10**	**HFIP** (4:1)	**61**	100:0	**0**

a Reaction conditions: **4** (0.20 mmol), (*R*,*R*)-Mn­(CF_3_–PDP) (10 mol %), ^Cl^AcOH (15 equiv), H_2_O_2_ (10 or 2 equiv), solvent:CH_2_Cl_2_, 0 or −36 °C, 3 h. Isolated yields, selectivity, and recovery of starting material (RSM) are averages of two runs.

b The yield corresponds to the total yield of products **5** and **6**.

Notably, the bulky Mn­(CF_3_–PDP) catalyst strongly favors sterically accessible sites over electron-rich ones, resulting in exclusive oxidation at C2, the only position flanked by two methylenes, even in the presence of more electron-rich sites such as C1. This same factor underlies the preferential equatorial C2 oxidation: the axial C2 C—H bond is sterically shielded by axial ester and methyl groups, leaving the equatorial C2 hydrogen significantly more exposed.^21^

The chemoselective synthesis of alcohol **5** in good yield promoted by HFIP enabled us to realize a second-generation synthesis for metabolite **3**, isolated from *Streptomyces griseus* ATCC 10137,^22^ in a route that would not require any use of protecting groups (Scheme 2). In addition, due to the strong ability of HFIP in interacting with polar moieties, we proposed a synthesis without the need to protect the C19 carboxylic acid as the corresponding methyl ester.

**Scheme 2:**
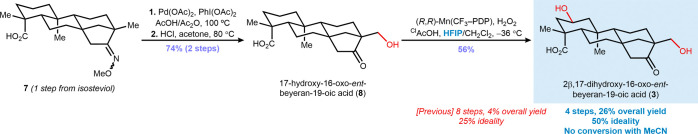
Second-Generation Synthesis of 2β,17-Dihydroxy-16-oxo-*ent*-beyeran-19-oic Acid (**3**)

Then, we submitted oxime **7** (obtained in one step from isosteviol) to Sanford acetoxylation^23^ followed by acid hydrolysis to furnish 17-hydroxy-16-oxo-*ent*-beyeran-19-oic acid (**8**) in 74% yield over two steps. Subsequent C—H oxidation of compound **8** catalyzed by (*R*,*R*)-Mn­(CF_3_–PDP) in HFIP/CH_2_Cl_2_ proceeded smoothly to afford 2β,17-dihydroxy-16-oxo-*ent*-beyeran-19-oic acid (**3**) as the only product in 56% yield.

The control experiment conducted in MeCN/CH_2_Cl_2_ resulted in no substrate conversion (see Supporting Information for experimental details), which we hypothesize is due to a nonanticipated chelation between the manganese center and the β-hydroxy ketone, leading to catalyst deactivation and preventing the desired oxidation.^24^ Although not observed in our experiments, we expect that an extensive screening of different carboxylic acids could enable lactone formation from compound **8**.^20^

The synthesis of metabolite **3** was accomplished in four steps from isosteviol in 26% overall yield, representing a substantial improvement facilitated by using HFIP. Compared to the previous synthetic route, this approach not only increased the overall yield and synthetic ideality but also reduced the number of steps by half. Most notably, this application demonstrated the effectiveness of fluorinated alcohols as important agents to increase ideality in remote late-stage C—H bond functionalization, emulating the ambitious selectivity of enzymes by leaving all other sites and functional groups of the molecule unaltered.

Since the oxidation of an *ent*-beyerane was smoothly achieved in a hydroxyl-containing intermediate, we envisioned performing the same approach to oxidize the C2 position in the more challenging *ent*-kaurane **10**. The first step consisted of a *syn*-dihydroxylation reaction with aqueous OsO_4_ and NMO to obtain 16α,17-dihydroxy-*ent*-kauran-18-oic acid (**10**) in 60% yield (Scheme 3). This intermediate was submitted to the same C—H bond oxidation conditions highlighted in the previous synthesis, furnishing natural product **11** in 40% yield, with no formation of byproducts. A control experiment conducted in MeCN/CH_2_Cl_2_ resulted in no substrate conversion, with complete recovery of the starting material.

**Scheme 3:**
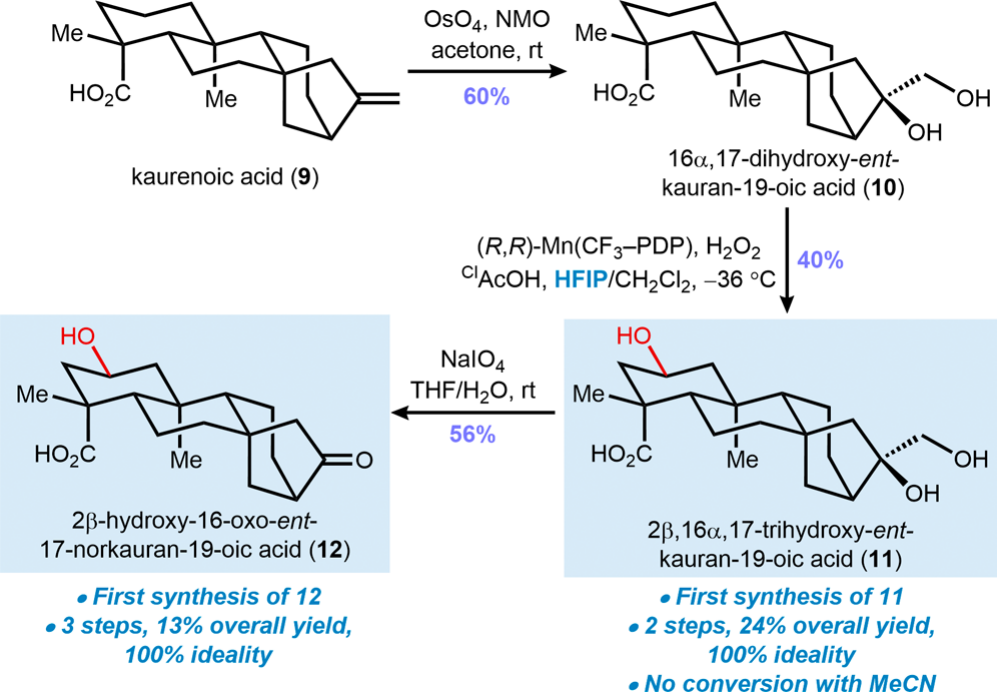
Synthesis of 2β,16α,17-Trihydroxy-*ent*-kauran-19-oic Acid (**11**) and 2β-Hydroxy-16-oxo-*ent*-17-norkauran-19-oic Acid (**12**)

This two-step procedure enabled the first synthesis of 2β,16α,17-trihydroxy-*ent*-kauran-19-oic acid (**11**)^26^ with no overoxidation of the diol moiety, in 24% overall yield and 100% of synthetic ideality, providing a practical and concise exhibition of how fluorinated solvents can improve the chemoselectivity in nondirected C—H bond oxidations. The oxidative cleavage of diol **11** with NaIO_4_ afforded 2β-hydroxy-16-oxo-*ent*-17-norkauran-19-oic acid (**12**) in 56% yield, the first reported synthesis of this natural product.^27^

The syntheses of **11** and **12** were both accomplished in 100% ideality from kaurenoic acid (**9**), providing 24% and 13% overall yield, respectively. By taking advantage of oxidized natural product intermediate **11**, we avoided an alternative dedicated route to **12**, which would require oxidative cleavage of **9**, followed by C2 functionalization. Instead, by performing a three-reaction route, we achieved improved step economy and ideality compared to two fully independent syntheses, thereby expanding the chemical space with higher efficiency.

With the preparation of the *ent-*kauranes **11** and **12** ensured, we decided to expand the beneficial use of HFIP in oxidations of terpenes using (*S*,*S*)-Fe­(PDP) to prepare tricalysiolide B (**14**) (Scheme 4).^28^ After a (*S*,*S*)-Fe­(PDP)-catalyzed epoxidation followed by a Pinnick oxidation,^29^ natural product **14** was delivered in 55% yield from the *ent*-kaurene cafestol (**13**).

**Scheme 4:**
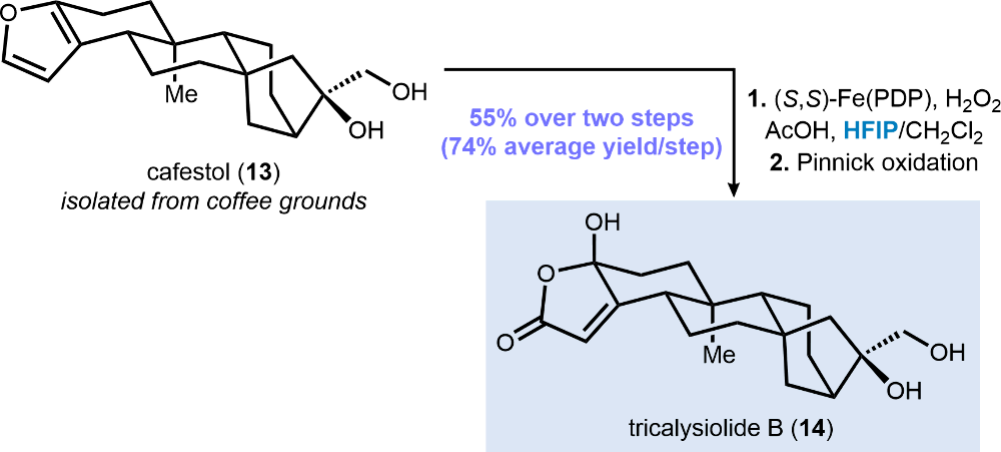
Synthesis of Tricalysiolide B (**14**)

Notably, the control experiment of **13** replacing HFIP with MeCN delivered the natural product **14** in 54% yield, indicating that the epoxidation occurs faster than the oxidation of the diol moiety regardless of the solvent used. Although fluorinated alcohols did not show a significant improvement for this reaction, we report a straightforward method to synthesize tricalysiolide B (**14**) without the need for protecting groups, achieving higher synthetic ideality compared to the previously report.^29^

In summary, we reported that the use of HFIP can be beneficial for chemoselective nondirected C—H bond oxidations, preventing both overoxidation of alcohols and diols and deactivation of the catalyst in complex natural products. We have shown this efficacy in the synthesis of *ent*-beyerane and *ent*-kaurane natural products in four steps or less from available starting materials. Through these syntheses, HFIP played a crucial role, in combination with the Mn­(CF_3_–PDP) catalyst, in delivering hydroxylated products in a late-stage scenario.

## Supplementary Material



## Data Availability

The data underlying this study are available in the published article and its Supporting Information. Raw NMR data files can be found online at DOI: 10.5281/zenodo.18270798.

## Notes

Additional experimental details and spectra data for all compounds (^1^H and ^13^C NMR, optical rotation, HRMS, and crystallographic data) (PDF)
