# Supplementary material for: Fluorinated Solvents for Chemoselective Oxidations: A Strategy toward Synthetic Ideality in Natural Product Synthesis
Source: ACS Org Inorg Au. 2026 Jan 23;6(1):47–52. doi: 10.1021/acsorginorgau.5c00121 (PMC12879165; doi:10.1021/acsorginorgau.5c00121)

Supporting Information

Fluorinated Solvents for Chemoselective Oxidations: A Strategy toward Synthetic Ideality in Natural Product Synthesis

Victor C. S. Santana,[1] Lucas D. P. Gonçalves,[1] Yasmin N. Salmazo,[1] Julian C. S. Pavan,[2] Deborah de A. Simoni,[1] Vladimir C. G. Heleno,[2] Emilio C. de Lucca, Jr.[1]*

[1] Instituto de Química, Universidade Estadual de Campinas (UNICAMP), 13083-970, Campinas, SP, Brazil

[2] Núcleo de Pesquisa em Ciências Exatas e Tecnológicas, Universidade de Franca (UNIFRAN), 14404-600, Franca, SP, Brazil

E-mail: eluccajr@unicamp.br

Table of Contents

I. General Section S2

II. Experimental Procedures S3

III. Natural Product Comparison S18

IV. Crystallographic Data S21

V. References S23

VI. Spectral Data S24

I. General Section

Yields refer to chromatographically and spectroscopically (^{1}H NMR) homogeneous material, unless otherwise stated.

Tetrahydrofuran (THF) was distilled and stored over 3 Å molecular sieves for at least 48 h prior to use.[1] Acetone and methanol (MeOH) were stored over 3 Å molecular sieves for at least 48 h prior to use.[1] 1,1,1,3,3,3-hexafluoro-2-propanol (HFIP) and 2,2,2-trifluoroethanol (TFE) were bought from Sigma-Aldrich® and used without further purification. Acetonitrile (MeCN) was bought in HPLC grade and used without further purification. Acetic anhydride (Ac_2O) was fractionally distilled from K_2CO_3 prior to use. The other reagents were used without further purification, unless otherwise stated.

The purification of reaction products was performed by flash column chromatography using silica gel (220‒440 mesh).[2] Reactions were monitored by thin layer chromatography carried out on silica-gel 60 plates with layer thickness of 175‒225 μm, and visualization was accomplished using phosphomolybdic acid or *p*-anisaldehyde staining followed by heating.

Optical rotations were measured on a polarimeter with a sodium lamp using a 1.0 cm cell and are reported as follows: $[\alpha]_D^T$ (°C) (*c* (g/100 mL), solvent).

^{1}H and proton-decoupled ^{13}C NMR spectra were recorded at 400 MHz (^{1}H) and 100 MHz (^{13}C), 500 MHz (^{1}H) and 125 MHz (^{13}C), or 600 MHz (^{1}H) and 150 MHz (^{13}C). Chemical shifts (δ) are reported in ppm using residual undeuterated solvent signals as internal standards ($CDCl_3$ at 7.26 ppm, CD_3OD at 3.31 ppm, $(CD)_3SO$ at 2.50 ppm, and C_5D_5N at 7.22 ppm for ^{1}H NMR spectra and $CDCl_3$ at 77.16 ppm, CD_3OD at 49.00 ppm, $(CD)_3SO$ at 39.52 ppm, and C_5D_5N at 123.87 ppm for ^{13}C NMR spectra). Multiplicity data are reported as follows: s = singlet, d = doublet, t = triplet, br s = broad singlet, br d = broad doublet, dd = doublet of doublets, td = triplet of doublets, tt = triplet of triplets, ddd = doublet of doublet of doublets, and m = multiplet. The multiplicity is followed by the coupling constant(s) in Hz and integration. The values of coupling constants were measured directly in ^{1}H NMR spectra. Structural assignments were made with additional information from gCOSY, gHSQC, and gHMBC experiments.

High-resolution mass spectrometry (HRMS) was measured using electrospray ionization (ESI).

Single crystal X-ray diffraction data collection was conducted at 120 K in a Bruker Apex II Duo CCD diffractometer, using a fine focus sealed tube of a MoKα (λ = 0.71073 Å) as the source of radiation and the data collection strategy consisted of sets of *phi* and *omega* scans.

II. Experimental Procedures

(–)-Isosteviol (SI-2):

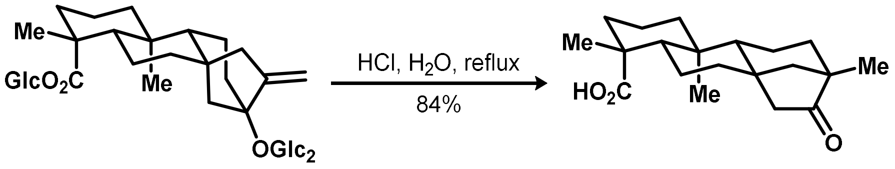

Isosteviol (**SI-2**) was prepared according to the literature procedure.[3] The ^{1}H and ^{13}C NMR data matched with those reported earlier.[4]

(–)-Methyl (4*R*,4a*S*,6a*R*,9*S*,11a*R*,11b*S*)-4,9,11b-trimethyl-8-oxotetradecahydro-6a,9-methanocyclohepta[a]naphthalene-4-carboxylate (4):

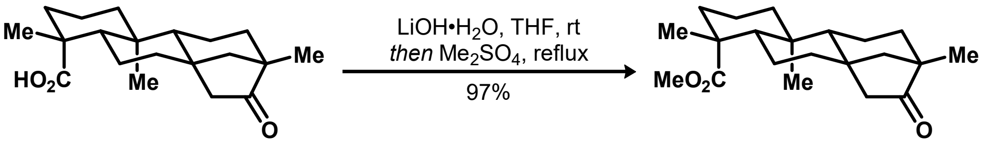

Compound **4** was prepared according to the literature procedure.[5]

^{1}H NMR (500 MHz, CDCl$_3$) δ 3.61 (s, 3H), 2.60 (dd, J = 18.6 and 3.8 Hz, 1H), 2.16 (br d, J = 13.3 Hz, 1H), 1.90–1.83 (m, 1H), 1.78 (d, J = 18.6 Hz, 1H), 1.80–1.73 (m, 1H), 1.71–1.61 (m, 4H), 1.60–1.55 (m, 1H), 1.52 (dd, J = 11.6 and 2.5 Hz, 1H), 1.47 (dd, J = 13.3 and 3.9 Hz, 1H), 1.43–1.31 (m, 3H), 1.24–1.14 (m, 2H), 1.16 (s, 3H), 1.10 (dd, J = 12.3 and 1.8 Hz, 1H), 1.00 (td, J = 13.5 and 4.3 Hz, 1H), 0.95 (s, 3H), 0.88 (td, J = 13.5 and 4.3 Hz, 1H), 0.66 (s, 3H).

^{13}C{^{1}H} NMR (125 MHz, CDCl$_3$) δ 222.6, 177.9, 57.1, 54.8, 54.4, 51.3, 48.8, 48.6, 43.9, 41.6, 39.9, 39.5, 38.0, 37.4, 28.9, 21.8, 20.4, 20.0, 19.0, 13.3.

Oxidation with Mn(CF_3-PDP) optimization

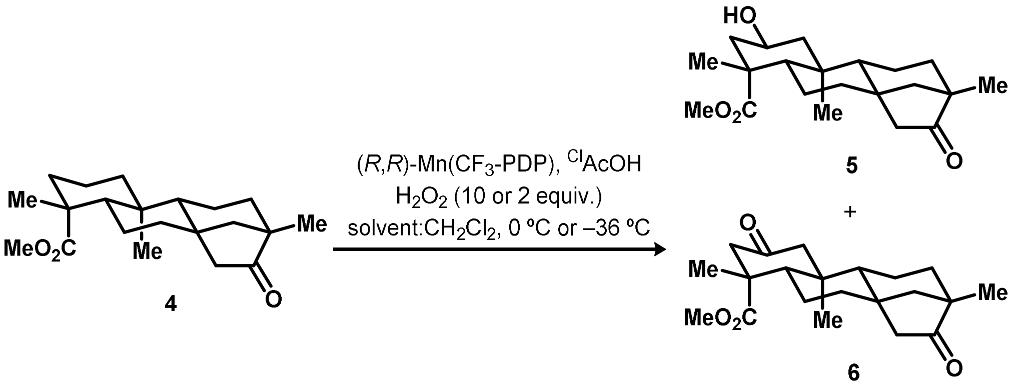

Table S1: C—H oxidation of compound **4** with (*R*,*R*)-Mn(CF_3-PDP).

Entry	T (ºC)	H_2O_2 (equiv.)	Solvent (solvent:CH_2Cl_2)	Total yield[a]	Selectivity (5:6)	RSM (%)
1	0	10	MeCN (1:1)	50	20:80	15
2	0	10	TFE (1:1)	56	22:78	0
3	0	10	HFIP (1:1)	49	75:25	0
4	–36	10	HFIP (1:1)	62	82:18	0
5	–36	2	HFIP (1:1)	58	83:17	24
**6**	**–36**	**10**	**HFIP (4:1)**	**61**	**100:0**	**0**

[a] The yield corresponds to the total yield of products **5** and **6**.

General procedure for Entries 1–5:

A vial was charged with ester **4** (66 mg, 0.20 mmol, 1.0 equiv.), (*R,R*)-Mn(CF_3-PDP) (27 mg, 0.02 mmol, 10 mol%), ^{Cl}AcOH (285 mg, 3.0 mmol, 15 equiv.), MeCN, HFIP, or TFE (0.3 mL), CH_2Cl_2 (0.3 mL), and a stir bar. The vial was cooled to 0 ºC with an ice/water bath or −36 ºC using a 1,2-dichloroethane/liquid nitrogen bath. A separate solution of H_2O_2 (137 mg, 2.0 mmol, 10 equiv., 50 wt.% aqueous solution) in MeCN, HFIP, or TFE (2.0 mL) and CH_2Cl_2 (0.5 mL) or H_2O_2 (27 mg, 0.40 mmol, 2 equiv., 50% w.t. aqueous solution) in HFIP (0.4 mL) and CH_2Cl_2 (0.1 mL) was loaded into a 3 mL or 1 mL syringe fitted with a 25 G needle and added dropwise to the reaction over 3 h via a syringe pump (0.833 mL/h addition rate or 0.166 mL/h addition rate) at 0 ºC or −36 ºC. After the H_2O_2 addition was complete, the reaction mixture was concentrated under reduced pressure, the residue was dissolved in CH_2Cl_2 (13 mL) and washed with saturated aqueous solution of $NaHCO_3$ (6 mL). The aqueous layer was extracted with CH_2Cl_2 (2 x 10 mL) and the combined organic layers were dried over Na_2SO_4, filtered, and concentrated under reduced pressure. The residue was purified by flash column chromatography (hexanes:EtOAc 60:40) to afford alcohol **5** and ketone **6** as white solids. X-

ray diffraction quality single-crystals were obtained by slow evaporation of a solution of compound **5** in $CHCl_3$.

Entry 1:

Run 1: alcohol **5**: 8.0 mg, 0.023 mmol, 12% yield; ketone **6**: 25.0 mg, 0.072 mmol, 36% yield; rsm: 12.0 mg, 0.036 mmol, 18%.

Run 2: alcohol **5**: 6.2 mg, 0.018 mmol, 9% yield; ketone **6**: 31.1 mg, 0.090 mmol, 45% yield; rsm: 7.7 mg, 0.023 mmol, 12%.

Average Overall Yield: alcohol **5**: 10%; ketone **6**: 40%; rsm: 15%.

Entry 2:

Run 1: alcohol **5**: 9.6 mg, 0.028 mmol, 14% yield; ketone **6**: 28.4 mg, 0.082 mmol, 41% yield.

Run 2: alcohol **5**: 6.9 mg, 0.020 mmol, 10% yield; ketone **6**: 33.2 mg, 0.096 mmol, 48% yield.

Average Overall Yield: alcohol **5**: 12%; ketone **6**: 44%.

Entry 3:

Run 1: alcohol **5**: 26.9 mg, 0.077 mmol, 39% yield; ketone **6**: 8.8 mg, 0.025 mmol, 13% yield.

Run 2: alcohol **5**: 24.4 mg, 0.070 mmol, 35% yield; ketone **6**: 8.3 mg, 0.024 mmol, 12% yield.

Average Overall Yield: alcohol **5**: 37%; ketone **6**: 12%.

Entry 4:

Run 1: alcohol **5**: 34.8 mg, 0.10 mmol, 50% yield; ketone **6**: 8.2 mg, 0.024 mmol, 12% yield.

Run 2: alcohol **5**: 37.2 mg, 0.11 mmol, 53% yield; ketone **6**: 6.9 mg, 0.020 mmol, 10% yield.

Average Overall Yield: alcohol **5**: 52%, ketone **6**: 11%.

Entry 5:

Run 1: alcohol **5**: 33.1 mg, 0.095 mmol, 48% yield; ketone **6**: 6.3 mg, 0.018 mmol, 9% yield; rsm: 22.5 mg, 0.068 mmol, 34%.

Run 2: alcohol **5**: 35.2 mg, 0.10 mmol, 51% yield; ketone **6**: 7.0 mg, 0.020 mmol, 10% yield; rsm: 12.7 mg, 0.038 mmol, 19%.

Average Overall Yield: alcohol **5**: 49%; ketone **6**: 9%; rsm: 26%.

Procedure for Entry 6:

A vial was charged with ester **4** (66 mg, 0.20 mmol, 1.0 equiv.), (*R,R*)-Mn(CF_3-PDP) (27 mg, 0.02 mmol, 10 mol%), ^{Cl}AcOH (285 mg, 3.0 mmol, 15 equiv.), HFIP (0.48 mL), CH_2Cl_2 (0.12 mL), and a stir bar. The vial was cooled to −36 ºC using a 1,2-dichloroethane/liquid nitrogen bath. A separate solution of H_2O_2 (137 mg, 2.0 mmol, 10 equiv., 50 wt.% aqueous solution) in HFIP (2.0 mL) and CH_2Cl_2 (0.5 mL) was loaded into a 3 mL syringe fitted with a 25 G needle, and added dropwise to the reaction over 3 h via a syringe pump (0.833 mL/h addition rate) at −36 ºC. After the H_2O_2 addition was complete, the reaction mixture was concentrated under reduced pressure, the residue was dissolved in CH_2Cl_2 (13 mL) and washed with saturated aqueous solution of $NaHCO_3$ (6 mL). The aqueous layer was extracted with CH_2Cl_2 (2 x 10 mL) and the combined organic layers were dried over Na_2SO_4, filtered, and concentrated under reduced pressure. The residue was purified by flash column chromatography (hexanes:EtOAc 60:40) to afford alcohol **5** as a white solid.

Run 1: alcohol **5**: 42.0 mg, 0.121 mmol, 60% yield**.**

Run 2: alcohol **5**: 43.2 mg, 0.124 mmol, 62% yield.

Average Overall Yield: alcohol **5**: 61%.

(–)-Methyl (2*S*,4*R*,4a*S*,6a*R*,9*S*,11a*R*,11b*S*)-2-hydroxy-4,9,11b-trimethyl-8-oxotetradecahydro-6a,9-methanocyclohepta[a]naphthalene-4-carboxylate (5):

TLC: R_f = 0.35 (hexanes:EtOAc 60:40).

Optical rotation: $[\alpha]_D^{20}$ –46 (*c* 0.95, $CHCl_3$).

^{1}H NMR (400 MHz, $CDCl_3$) δ 4.15–4.07 (m, 1H), 3.63 (s, 3H), 2.59 (dd, *J* = 18.6 and 3.8 Hz, 1H), 2.45 (ddd, *J* = 12.5, 4.3 and 2.1 Hz, 1H), 2.07 (ddd, *J* = 12.1, 4.3 and 2.0 Hz, 1H) 1.93–1.88 (m, 1H), 1.81 (d, *J* = 18.6 Hz, 1H), 1.77–1.71 (m, 1H), 1.61–1.62 (m, 1H), 1.67–1.62 (m, 1H), 1.66–1.57 (m, 1H), 1.59–1.52 (m, 1H), 1.53–1.47 (m, 1H), 1.44–1.38 (m, 1H), 1.43–1.34 (m, 1H), 1.26 (s, 3H), 1.27–1.22 (m, 2H), 1.11 (dd, *J* = 11.8 and 2.0 Hz, 1H), 1.04–1.01 (m, 1H), 0.97 (s, 3H), 0.81 (t, *J* = 11.8 Hz, 1H), 0.69 (s, 3H).

^{13}C{^{1}H} NMR (100 MHz, $CDCl_3$) δ 222.2, 177.4, 64.2, 56.5, 54.7, 54.3, 51.7, 48.8, 48.7, 48.6, 46.9, 45.1, 41.4, 39.5, 39.4, 37.2, 28.9, 21.5, 20.6, 19.9, 14.4.

(–)-Methyl (4R,4aS,6aR,9S,11aR,11bS)-4,9,11b-trimethyl-2,8-dioxotetradecahydro-6a,9-methanocyclohepta[a]naphthalene-4-carboxylate (6):

TLC: R_f = 0.50 (hexanes:EtOAc 60:40).

Optical rotation: $[\alpha]_D^{20}$ –51 (*c* 1.77, $CHCl_3$).

^{1}H NMR (500 MHz, $CDCl_3$) δ 3.61 (s, 3H), 2.90 (dd, *J* = 14.1 and 2.2 Hz, 1H), 2.52 (dd, *J* = 18.6 and 3.7 Hz, 1H), 2.44 (dd, *J* = 13.8 and 2.1 Hz, 1H), 2.09 (d, *J* = 14.1 Hz, 1H), 2.05–2.02 (m, 1H), 1.93 (d, *J* = 13.8 Hz, 1H), 1.81 (d, *J* = 18.6 Hz, 1H), 1.76–1.65 (m, 1H), 1.76–1.71 (m, 1H), 1.70–1.64 (m, 1H), 1.65–1.60 (m, 1H), 1.62–1.50 (m, 1H), 1.62–1.58 (m, 1H), 1.60–1.52 (m, 1H), 1.45–1.41 (m, 1H), 1.42–1.34 (m, 1H), 1.41–1.37 (m, 1H), 1.37 (s, 1H), 1.28–1.19 (m, 1H), 0.98 (s, 3H), 0.69 (s, 3H).

^{13}C{^{1}H} NMR (125 MHz, $CDCl_3$) δ 221.5, 207.9, 175.9, 56.2, 55.3, 54.2, 54.0, 52.1, 51.4, 48.7, 48.0, 47.9, 42.1, 40.8, 39.6, 37.1, 28.6, 21.5, 20.4, 19.9, 14.5.

(–)-(4*R*,4a*S*,6a*R*,9*S*,11a*R*,11b*S*)-8-(methoxyimino)-4,9,11b-trimethyltetradecahydro-6a,9-methanocyclohepta[a]naphthalene-4-carboxylic acid (7):

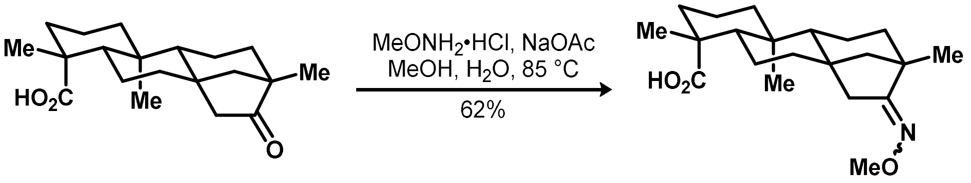

SI-2 7

A round-bottom flask was charged with isosteviol (**SI-2**) (2.0 g, 6.28 mmol, 1.0 equiv.), $MeONH_2$•HCl (10.5 g, 126 mmol, 20 equiv.), NaOAc (7.70 g, 93.9 mmol, 15 equiv.), a mixture of MeOH/H_2O (5:1) (63 mL, 0.1 M), and a stir bar. The flask was warmed to 85 ºC in an oil bath and stirred for 17 h. Then, the reaction mixture was allowed to cool to room temperature, concentrated under reduced pressure and the residue was partitioned between CH_2Cl_2 (60 mL) and H_2O (60 mL). The aqueous layer was extracted with CH_2Cl_2 (3 × 60 mL), the combined organic layers were dried over Na_2SO_4, filtered, and concentrated under reduced pressure. The residue was purified by flash column chromatography (hexanes:EtOAc 80:20) to afford oxime **7** (1.36 g, 3.91 mmol, 62%) as a white solid.

TLC: R_f = 0.70 (hexanes:EtOAc 75:25).

Optical rotation: $[\alpha]_D^{20}$ –52 (*c* 1.7, $CHCl_3$).

^{1}H NMR (500 MHz, $CDCl_3$) δ 3.83 (s, 3H), 2.89 (dd, *J* = 18.5 and 3.8 Hz, 1H), 2.16 (br d, *J* = 13.4 Hz, 1H), 1.92 (d, *J* = 18.7 Hz, 1H), 1.88–1.79 (m, 2H), 1.75 (m, 2H), 1.66–1.55 (m, 3H), 1.45–1.26 (m, 4H), 1.31–1.21 (m, 1H), 1.24 (s, 3H), 1.18 (dd, *J* = 11.2 and 3.1 Hz, 1H), 1.13–1.04 (m, 2H), 1.10 (s, 3H), 1.01 (td, *J* = 13.6 and 4.1 Hz, 1H), 0.88 (td, *J* = 13.1 and 3.9 Hz, 1H), 0.85 (s, 3H).

^{13}C{^{1}H} NMR (125 MHz, $CDCl_3$) δ 184.1, 169.6, 61.5, 57.3, 56.6, 55.1, 43.83, 43.78, 41.0, 40.7, 40.0, 39.7, 38.3, 37.9, 37.5, 29.2, 22.4, 21.7, 20.6, 19.0, 13.6.

HRMS (ESI TOF-MS) *m/z* calcd for $C_{21}H_{34}NO_3$ $[M + H]^+$: 348.2528, found: 348.2533.

(–)-17-hydroxy-16-oxo-*ent*-beyeran-19-oic acid (8):

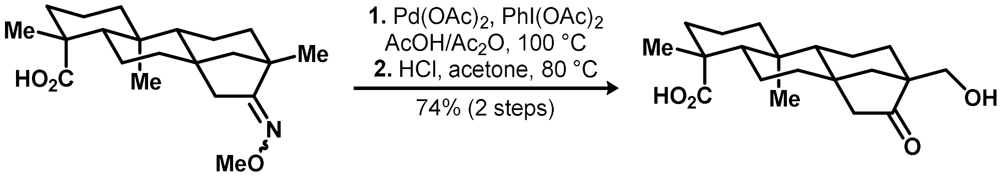

7 8

A tube was charged with oxime **7** (417 mg, 1.20 mmol, 1.0 equiv.), a mixture of AcOH/Ac_2O (1:1) (8.0 mL, 0.15 M), $Pd(OAc)_2$ (14.0 mg, 0.062 mmol, 5 mol%), and $PhI(OAc)_2$ (581 mg, 1.80 mmol, 1.5 equiv.). The tube was sealed, warmed to 100 ºC in a stainless-steel heating block, and stirred for 43 h. Then, the reaction mixture was allowed to cool to room temperature, diluted with EtOAc (50 mL) and washed with H_2O (4 x 50 mL). The organic layer was dried over Na_2SO_4, filtered, and concentrated under reduced pressure. The residue was dissolved in acetone (24 mL, 0.05 M) and treated with aqueous 1M HCl (12 mL). The mixture was warmed to 80 ºC in a stainless-steel heating block and stirred for 5 days. Then, the reaction mixture was allowed to cool to room temperature and concentrated under reduced pressure. The residue was dissolved in CH_2Cl_2 (100 mL) and washed with H_2O (2 × 50 mL). The organic layer was dried over Na_2SO_4, filtered, and concentrated under reduced pressure. The residue was purified by flash column chromatography (hexanes:EtOAc 50:50) to afford 17-hydroxy-16-oxo-*ent*-beyeran-19-oic acid (**8**) (298 mg, 0.89 mmol, 74%) as a white solid.

TLC: R_f = 0.50 (hexanes:EtOAc 60:40).

Optical rotation: $[\alpha]_D^{20}$ –55 (*c* 0.98, $CHCl_3$).

^{1}H NMR (500 MHz, $CDCl_3$) δ 3.64 (d, *J* = 11.5 Hz, 1H), 3.52 (d, *J* = 11.5 Hz, 1H), 2.67 (dd, *J* = 18.9 and 3.7 Hz, 1H), 2.17 (d, *J* = 13.4 Hz, 1H), 1.96–1.83 (m, 1H), 1.88–1.82 (m, 1H), 1.89–1.83 (m, 2H), 1.84–1.73 (m, 1H), 1.84–1.71 (m, 1H), 1.83–1.71 (m, 1H), 1.82–1.78 (m, 1H), 1.77–1.72 (m, 1H), 1.73–1.67 (m, 1H), 1.52 (td, 13.5 and 3.8 Hz, 1H), 1.49–1.42 (m, 1H), 1.42–1.33 (m, 1H), 1.33–1.29 (m, 1H), 1.32–1.25 (m, 1H), 1.29–1.25 (m, 1H), 1.29–1.23 (m, 1H), 1.25 (s, 3H), 1.17 (d, *J* = 10.5 Hz, 1H), 1.03 (td, *J* = 13.5 and 4.1 Hz, 1H), 0.93 (td, *J* = 13.3 and 4.0 Hz, 1H), 0.79 (s, 3H).

$^{13}C\{^1H\}$ NMR (125 MHz, $CDCl_3$) δ 223.5, 183.4, 65.2, 57.1, 55.5, 54.2, 49.1, 49.0, 43.8, 41.4, 39.8 (CH_2, C_0), 38.4, 37.8, 32.2, 29.1, 21.7, 20.0, 19.0, 13.5.

HRMS (ESI TOF-MS) *m/z* calcd for $C_{20}H_{29}O_4$ $[M – H]^-$: 333.2060, found: 333.2080.

(–)-2β,17-dihydroxy-16-oxo-*ent*-beyeran-19-oic acid (3):

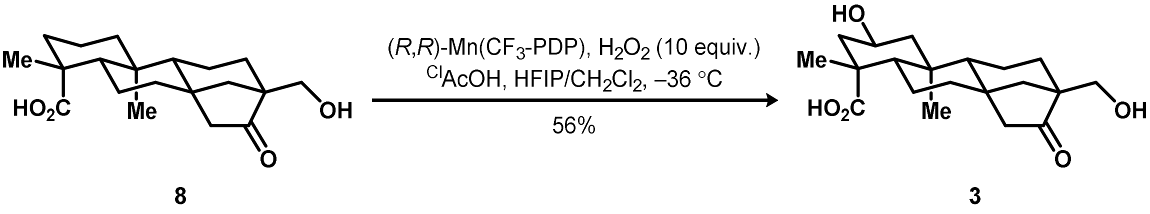

A vial was charged with compound **8** (67 mg, 0.20 mmol, 1.0 equiv.), (*R*,*R*)-Mn(CF_3-PDP) (27 mg, 0.02 mmol, 10 mol%), ^{Cl}AcOH (285 mg, 3.0 mmol, 15 equiv.), HFIP (0.48 mL), CH_2Cl_2 (0.12 mL), and a stir bar. The vial was cooled to –36 ºC using a 1,2-dichloroethane/liquid nitrogen bath. A separate solution of H_2O_2 (137 mg, 2.0 mmol, 10 equiv., 50 wt.% aqueous solution) in HFIP (2.0 mL) and CH_2Cl_2 (0.5 mL) was loaded into a 3 mL syringe fitted with a 25 G needle and added dropwise to the reaction over 3 h via a syringe pump (0.833 mL/h addition rate) at −36 ºC. After the H_2O_2 addition was complete, the reaction mixture was concentrated under reduced pressure, the residue was dissolved in CH_2Cl_2 (13 mL) and washed with H_2O (6 mL). The aqueous layer was extracted with CH_2Cl_2 (2 × 10 mL) and EtOAc (2 × 10 mL), and the combined organic layers were dried over Na_2SO_4, filtered, and concentrated under reduced pressure. The residue was purified by flash column chromatography (EtOAc:MeOH 98:2) to afford 2β,17-dihydroxy-16-oxo-*ent*-beyeran-19-oic acid (**3**) as a white solid.

Run 1: 41.7 mg, 0.12 mmol, 59% yield.

Run 2: 36.3 mg, 0.10 mmol, 52% yield.

Average overall yield: 56%.

TLC: R_f = 0.40 (EtOAc:MeOH 98:2).

Optical rotation: $[\alpha]_D^{20}$ –50 (*c* 0.095, MeOH).

^{1}H NMR (500 MHz, C_5D_5N) δ 4.85–4.76 (m, 1H), 4.19 (d, J = 10.8 Hz, 1H), 3.74 (d, J = 10.8 Hz, 1H), 3.07 (dd, J = 12.1 and 2.6 Hz, 1H), 2.75 (dd, J = 18.3 and 3.4 Hz, 1H), 2.39 (dd, J = 12.0 and 3.5 Hz, 1H), 2.30 (dd, J = 11.2 and 1.4 Hz, 1H), 2.15–2.02 (m, 2H), 1.91 (d, J = 18.3 Hz, 1H), 1.80–1.74 (m, 1H), 1.73–1.66 (m, 1H), 1.65–1.58 (m, 1H), 1.57–1.48 (m, 1H), 1.57–1.47 (m, 1H), 1.54–1.49 (m, 1H), 1.50–1.41 (m, 1H), 1.48 (s, 3H), 1.39–1.29 (m, 1H), 1.33–1.23 (m, 1H), 1.28–1.21 (m, 1H), 1.25–1.16 (m, 1H), 1.07 (s, 3H).

$^{13}C\{^1H\}$ NMR (125 MHz, C_5D_5N) δ 220.3, 180.1, 63.8, 63.6, 56.6, 55.5, 55.2, 50.0, 49.8, 49.2, 48.5, 45.3, 41.8, 39.8, 39.6, 32.8, 29.4, 22.3, 20.3, 14.9.

Control experiment: the reaction was performed following the procedure above, replacing HFIP with MeCN. Under these conditions, starting material was completely recovered.

(–)-kaurenoic acid (9):

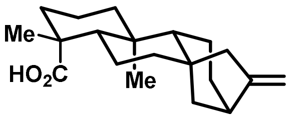

kaurenoic acid (**9**)

Kaurenoic acid (**9**) was isolated from *Mikania glomerata* leaves as described in literature.[6]

^1H NMR (500 MHz, $CDCl_3$) δ 4.79 (s, 1H), 4.74 (s, 1H), 2.64 (br s, 1H), 2.16 (br d, J = 14.5 Hz, 1H), 2.10–2.03 (m, 2H), 1.99 (dd, J = 11.3 and 1.6 Hz, 1H), 1.91–1.86 (m, 2H), 1.85–1.78 (m, 2H), 1.65–1.53 (m, 3H), 1.51 (t, J = 3.2 Hz, 1H); 1.49–1.40 (m, 3H), 1.24 (s, 3H), 1.13 (dd, J = 11.3 and 4.9 Hz, 1H), 1.09–1.04 (m, 2H), 1.01 (td, J = 13.6 and 4.3 Hz, 1H), 0.95 (s, 3H), 0.81 (td, J = 13.3 and 3.8 Hz, 1H).

$^{13}C\{^1H\}$ NMR (125 MHz, $CDCl_3$) δ 184.4, 156.1, 103.1, 57.2, 55.2, 49.1, 44.4, 44.0, 43.9, 41.4, 40.8, 39.84, 39.81, 37.9, 33.2, 29.1, 22.0, 19.2, 18.6, 15.7.

(–)-16α,17-dihydroxy-*ent*-kauran-19-oic acid (10):

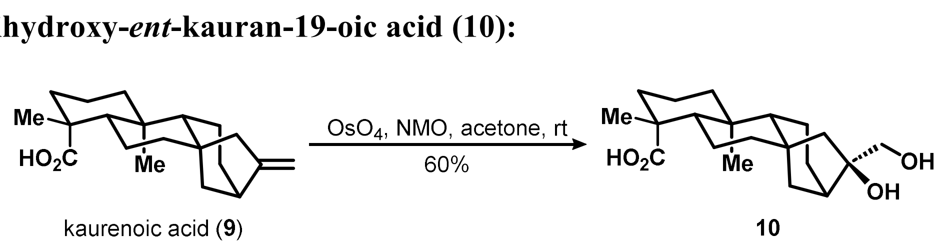

A round-bottom flask was charged with kaurenoic acid (**9**) (60 mg, 0.20 mmol, 1.0 equiv.), acetone (4 mL, 0.05 M), and a stir bar. Then, OsO_4 (44 µL, 7.2 µmol, 3.6 mol%, 4 wt.% aqueous solution), *N*-methyl-morpholine *N*-oxide (35 mg, 0.30 mmol, 1.5 equiv.) were sequentially added to the solution and the reaction was stirred for 16 h at room temperature under nitrogen atmosphere. The reaction

mixture was quenched with saturated aqueous solution of $Na_2S_2O_3$ (5 mL) and the aqueous layer was extracted with EtOAc (3 × 10 mL). The combined organic layers were washed with brine (10 mL), dried over Na_2SO_4, and concentrated under reduced pressure. The residue was purified by flash column chromatography ($CHCl_3$:MeOH 90:10) to afford diol **10** (40 mg, 0.12 mmol, 60%) as a white solid.

TLC: R_f = 0.40 ($CHCl_3$:MeOH 90:10).

Optical rotation: $[\alpha]_D^{20}$ –85 (*c* 1.2, MeOH).

^{1}H NMR (500 MHz, CD_3OD) δ 3.70 (d, *J* = 11.3 Hz, 1H), 3.60 (d, *J* = 11.3 Hz, 1H), 2.12 (br d, *J* = 12.8 Hz, 1H), 2.02 (br s, 1H), 1.95–1.89 (m, 1H), 1.95–1.89 (m, 1H), 1.90–1.79 (m, 2H), 1.89–1.83 (m, 1H), 1.67–1.61 (m, 1H), 1.66–1.59 (m, 1H), 1.65–1.59 (m, 1H), 1.64–1.56 (m, 2H), 1.53 (br d, *J* = 14.3 Hz, 1H), 1.53–1.45 (m, 1H), 1.49–1.42 (m, 1H), 1.41–1.39 (m, 1H), 1.40 (br d, *J* = 14.3 Hz, 1H), 1.18 (s, 3H), 1.11–1.04 (m, 1H), 1.05–0.97 (m, 1H), 1.04–0.99 (m, 1H), 0.98 (s, 3H), 0.83 (td, *J* = 13.1 and 4.5 Hz, 1H).

^{13}C{^{1}H} NMR (125 MHz, CD_3OD) δ 181.6, 82.9, 66.8, 58.1, 57.4, 53.7, 46.2, 45.8, 44.6, 43.3, 41.9, 40.8, 39.2, 38.2, 29.4, 27.2, 23.5, 20.3, 19.6, 16.2.

HRMS (ESI TOF-MS) *m/z* calcd for $C_{20}H_{33}O_4$ $[M + H]^+$: 337.2373, found: 337.2376.

(–)-2β,16α,17-trihydroxy-*ent*-kauran-19-oic acid (11):

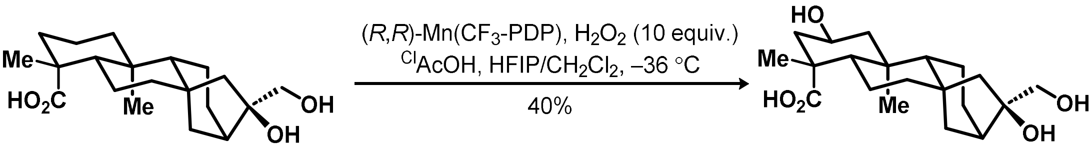

10 11

A vial was charged with diol **10** (67 mg, 0.20 mmol, 1.0 equiv.), (*R*,*R*)-Mn(CF_3-PDP) (27 mg, 0.02 mmol, 10 mol%), ^{Cl}AcOH (285 mg, 3.0 mmol, 15 equiv.), HFIP (0.48 mL), CH_2Cl_2 (0.12 mL), and a stir bar. The vial was cooled to –36 ºC with a 1,2-dichloroethane/liquid nitrogen bath. A separate solution of H_2O_2 (137 mg, 2.0 mmol, 10 equiv., 50 wt.% aqueous solution) in HFIP (2.0 mL) and CH_2Cl_2 (0.5 mL) was loaded into a 3 mL syringe fitted with a 25 G needle and added dropwise to the reaction over 3 h via a syringe pump (0.833 mL/h addition rate) at −36 ºC. After the H_2O_2 addition was complete, the reaction mixture was concentrated under reduced pressure, the residue was dissolved in CH_2Cl_2 (13 mL) and washed with H_2O (6 mL). The aqueous layer was extracted with CH_2Cl_2 (2 × 10 mL) and

EtOAc (2 × 10 mL), the combined organic layers were dried over Na_2SO_4, filtered, and concentrated under reduced pressure. The residue was purified by flash column chromatography ($CHCl_3$:MeOH 85:15) to afford 2β,16α,17-trihydroxy-*ent*-kauran-19-oic acid (**11**) as a white solid.

Run 1: 24.5 mg, 0.070 mmol, 35% yield.

Run 2: 32.7 mg; 0.093 mmol, 46% yield.

Average overall yield: 40%.

TLC: R_f = 0.50 ($CHCl_3$:MeOH 85:15).

Optical rotation: $[\alpha]_D^{20}$ –54 (*c* 1.0, MeOH).

^{1}H NMR (500 MHz, $(CD_3)_2SO$) *δ* 12.07 (br s, 1H), 4.33 (br s, 2H), 3.85 (br s, 1H), 3.86 (m, 1H), 3.50 (dd, *J* = 10.8 and 4.1 Hz, 1H), 3.40 (dd, *J* = 10.8 and 4.1 Hz, 1H), 2.20 (dd, *J* = 11.9 and 2.1 Hz, 1H), 2.00 (dd, *J* = 12.2 and 3.1 Hz 1H), 1.87 (br s, 1H), 1.69 (m, 2H), 1.69 (m, 1H), 1.54 (m, 1H), 1.53 (m, 1H), 1.51 (m, 2H), 1.50 (m, 1H), 1.39 (d, *J* = 15.4 Hz, 1H), 1.36 (m, 1H), 1.34 (m, 1H), 1.25 (d, *J* = 15.4 Hz, 1H), 1.13 (s, 3H), 0.92 (m, 2H), 0.86 (s, 3H), 0.79 (t, *J* = 11.9 Hz, 1H), 0.54 (t, *J* = 11.9 Hz, 1H).

^{13}C{^{1}H} NMR (125 MHz, $(CD_3)_2SO$) *δ* 178.5, 80.5, 65.3, 62.1, 55.4, 55.3, 52.7, 49.7, 47.0, 44.5, 43.95, 43.94, 41.8, 40.3, 36.9, 28.5, 25.8, 21.7, 18.2, 16.4.

HRMS (ESI TOF-MS) *m/z* calcd for $C_{20}H_{32}O_5Na$ $[M + Na]^+$: 375.2142, found: 375.2144.

Control experiment: the reaction was performed following the procedure above, replacing HFIP with MeCN. Under these conditions, starting material was completely recovered.

(–)-2β-hydroxy-16-oxo-*ent*-17-norkauran-19-oic acid (12):

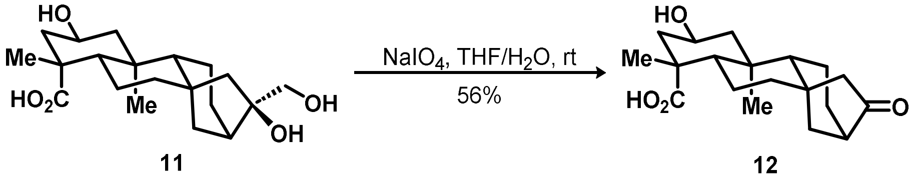

A vial was charged with triol **11** (18 mg, 0.051 mmol, 1.0 equiv.), a mixture of THF/H_2O (1:1) (1 mL, 0.05 M), $NaIO_4$ (40 mg, 0.19 mmol, 3.7 equiv.) and a stir bar. The reaction was stirred at room temperature for 18 hours. Then, the mixture was partitioned between EtOAc (6 mL) and H_2O (4 mL), and the aqueous phase was extracted with EtOAc (2 × 6 mL). The combined organic layers were dried over Na_2SO_4, filtered, and concentrated under reduced pressure. The residue was purified by flash column chromatography (hexanes:acetone 60:40) to afford 2β-hydroxy-16-oxo-*ent*-17-norkauran-19-oic acid (**12**) (9.2 mg, 0.029 mol, 56% yield) as a white solid.

TLC: R_f = 0.30 (hexanes:acetone 60:40).

Optical rotation: $[\alpha]_D^{20}$ –46 (*c* 0.75, MeOH).

^{1}H NMR (500 MHz, C_5D_5N) δ 4.84 (tt, *J* = 11.1 and 4.5 Hz, 1H), 3.10 (dd, *J* = 12.4 and 4.1 Hz, 1H), 2.55 (dd, *J* = 10.8 and 2.7 Hz, 1H), 2.32 (br s, 1H), 2.23 (m, 1H), 2.14 (dd, *J* = 12.0 and 3.0 Hz, 1H), 2.08 (m, 1H), 2.00 (m, 2H), 1.76 (m, 1H), 1.71 (m, 1H), 1.59 (m, 1H), 1.57 (m, 1H), 1.52 (m, 1H), 1.44 (m, 1H), 1.47 (s, 3H), 1.30 (m, 1H), 1.28 (m, 1H), 1.23 (m, 1H), 1.23 (s, 3H), 1.18 (br d, *J* = 12.2 Hz, 1H), 1.12 (t, *J* = 11.6 Hz, 1H).

^{13}C{^{1}H} NMR (125 MHz, C_5D_5N) δ 221.1, 180.4, 64.0, 56.7, 55.3, 54.3, 51.0, 48.8, 48.3, 45.6, 42.8, 41.7, 41.5, 37.7, 29.9, 29.7, 21.6, 19.4, 18.0.

HRMS (ESI TOF-MS) *m/z* calcd for $C_{19}H_{27}O_4$ $[M – H]^–$: 319.1915, found: 319.1906.

(–)-Cafestol (13):

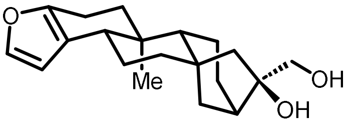

cafestol (**13**)

~0.8 kilograms of spent coffee grounds was continuously extracted using a Soxhlet extractor for 16h with refluxing hexanes. The combined hexanes extracts were concentrated under reduced pressure, providing ~70g coffee oil that was treated with MeOH (42 mL) and KOH (2.2 g). This reactional mixture was stirred for 2.5h at room temperature under a nitrogen atmosphere and was thereafter partitioned between heptane (150 mL) and MeOH (150 mL) containing 10% H_2O. The organic layer was extracted with MeOH/H_2O (10:1) (3 × 150 mL) and the combined methanolic extracts were concentrated under reduced pressure. The resulting oil was treated with KOH (4.3 g) at 40 °C and stirred for 0.5 h. The crude mixture was partitioned between H_2O (150 mL) and CH_2Cl_2/MeOH (10:1) (150 mL) and the aqueous layer was extracted with CH_2Cl_2/MeOH (10:1) (5 × 150 mL). The combined organic layers were concentrated under reduced pressure and the crude residue was diluted in EtOAc (33 mL) and treated with activated carbon (2.0 g) at room temperature for 1 h, with further filtration on filter paper and concentrated under reduced pressure. The residue was purified by flash column chromatography (EtOAc:hexanes 50:50 → 100:0) to obtain a mixture of cafestol and kahweol (3.2 g, approximately 1:1 ratio, ~10 mmol). The cafestol/kahweol mixture was directly treated with EtOH (100 mL, 0.1 M), quinoline (22 µL, 0.19 mmol, 2 mol%) and Pd/C (5 wt.%) (220 mg, 0.10 mmol, 1 mol%) under a atmosphere of H_2 (balloon) for 1 h.[7] Then, the mixture was filtered through a pad of Celite® with EtOAc, and concentrated under reduced pressure. The residue was purified by flash column chromatography (100% EtOAc) to obtain cafestol (**13**) as a light-yellow solid (3.0 g, 9.48 mmol).

TLC: R_f = 0.50 (EtOAc 100%).

Optical rotation: $[\alpha]_D^{20}$ –92 (c 0.90, $CHCl_3$).

^{1}H NMR (600 MHz, CD_3OD) δ 7.24 (s, 1H), 6.21 (s, 1H), 3.74 (d, J = 11.3 Hz, 1H), 3.64 (d, J = 11.3 Hz, 1H), 2.58 (d, J = 7.1 Hz, 1H), 2.26 (d, J = 12.7 Hz, 1H), 2.12–2.00 (m, 3H), 1.81 (d, J = 13.0 Hz, 1H), 1.77–1.61 (m, 7H), 1.58–1.49 (m, 3H), 1.47 (d, J = 14.6 Hz, 1H), 1.31–1.17 (m, 2H), 0.84 (s, 3H).

^{13}C{^{1}H} NMR (150 MHz, CD_3OD) δ 149.7, 141.8, 121.4, 109.1, 82.9, 66.9, 54.1, 53.7, 46.4, 45.7, 45.6, 42.2, 39.8, 39.2, 37.0, 27.1, 24.2, 21.5, 20.0, 13.7.

HRMS (ESI TOF-MS) *m/z* calcd for $C_{20}H_{29}O_3$ $[M + H]^+$: 317.2111, found: 317.2109.

(–)-Tricalysiolide B (14):

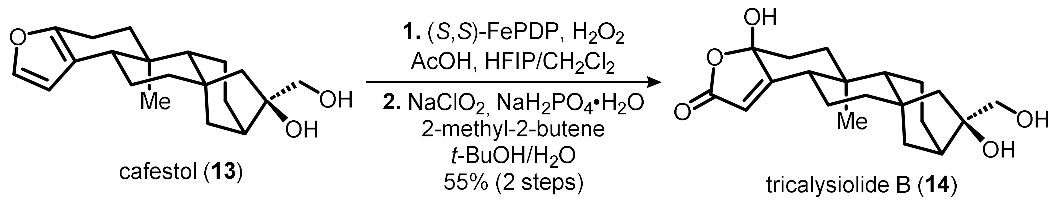

A vial was charged with cafestol (**13**) (63 mg, 0.20 mmol, 1.0 equiv.), (*S*,*S*)-Fe(PDP) (1.8 mg, 2.0 μmol, 1 mol%), AcOH (13 μL, 2.0 μmol, 1 mol%, 0.15 M solution of AcOH in HFIP/CH_2Cl_2 (4:1)), HFIP (0.24 mL), CH_2Cl_2 (0.06 mL), and a stir bar. A separate solution of H_2O_2 (11 μL, 0.20 mmol, 1.0 equiv., 50% wt. aqueous solution) in HFIP (1.44 mL) and CH_2Cl_2 (0.36 mL) was loaded into a 3 mL syringe fitted with a 25 G needle, and added over 1 min via a syringe pump (1.80 mL/min addition rate). The reaction was stirred at room temperature for 5 min, then filtered through a short silica plug (100% EtOAc + 1% Et_3N) and concentrated under reduced pressure. The crude mixture was treated directly with *t*-BuOH (2.0 mL), H_2O (0.8 mL), 2-methyl-2-butene (210 μL, 2.0 mmol, 10 equiv.), $NaH_2PO_4 \cdot H_2O$ (207 mg, 1.5 mmol, 7.5 equiv.), $NaClO_2$ (134 mg, 1.2 mmol, 5.9 equiv., 80% purity), stirred at room temperature for 2 h, and then concentrated under reduced pressure. The residue was purified by flash column chromatography (100% EtOAc) to yield tricalysiolide B (**14**) as a white solid.

Run 1: 36.0 mg, 0.10 mmol, 52% yield.

Run 2: 41.0 mg, 0.12 mmol, 59% yield.

Average overall yield: 55%.

TLC: R_f = 0.30 (EtOAc 100%).

Optical rotation: $[\alpha]_D^{20}$ –156 (*c* 1.5, MeOH).

^{1}H NMR (500 MHz, C_5D_5N) δ 9.52 (br s, 1H), 5.82 (s, 1H), 4.15 (d, *J* = 10.9 Hz, 1H), 4.07 (d, *J* = 10.9 Hz, 1H), 2.58 (d, *J* = 9.2 Hz, 1H), 2.53 (d, *J* = 13.8 Hz, 1H), 2.48 (br s, 1H), 2.06 (m, 1H), 1.99 (m, 1H), 1.97 (m, 1H), 1.90 (m, 1H), 1.85 (d, *J* = 14.2 Hz, 1H), 1.76 (m, 1H), 1.75 (d, *J* = 14.2 Hz,

1H), 1.74 (m, 1H), 1.63 (m, 1H), 1.57 (m, 1H), 1.56 (m, 1H), 1.54 (m, 1H), 1.51 (m, 1H), 1.49 (m, 1H), 1.39 (m, 1H), 1.30 (d, J = 8.5 Hz, 1H), 0.80 (s, 3H).

$^{13}C\{^1H\}$ NMR (125 MHz, C_5D_5N) δ 174.2, 171.9, 112.8, 106.0, 81.9, 66.7, 54.1, 54.0, 47.7, 46.2, 44.9, 44.1, 40.6, 38.4, 36.4, 35.3, 26.7, 22.4, 19.8, 14.8.

HRMS (ESI TOF-MS) *m/z* calcd for $C_{20}H_{29}O_5$ $[M + H]^+$: 349.2009, found: 349.2005.

Note: at 380 mg scale of cafestol (**13**) (1.2 mmol), the reaction afforded tricalysiolide B (**14**) in 55% yield (231 mg, 0.66 mmol).

Control experiment: the reaction was performed following the procedure above, replacing HFIP with MeCN. Under these conditions, the reaction afforded tricalysiolide B (**14**) in 54% yield (duplicate experiments).

III. Natural Product Comparison

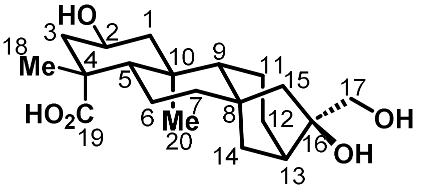

11

Table S2. ^{1}H and ^{13}C NMR chemical shifts for natural and synthetic 2β,16α,17-trihydroxy-*ent*-kauran-19-oic acid (**11**).

**Natural Product (500 MHz, $(CD_3)_2SO$)**[8]				**Synthetic Product (500 MHz, $(CD_3)_2SO$)**			
Position	δ^{13}C	δ^{1}H	multiplicity (*J* in Hz)	Position	δ^{13}C	δ^{1}H	multiplicity (*J* in Hz)
1a<br>1b	49.7	1.99<br>0.54	dd (11.5, 3.7)<br>t (11.5)	1a<br>1b	49.7	2.00<br>0.54	dd (12.2, 3.1)<br>t (11.9)
2a<br>2b	62.2	3.83	m	2	62.1	3.86	m
3a<br>3b	46.9	2.20<br>0.79	dd (11.7, 3.4)<br>t (11.7)	3a<br>3b	47.0	2.20<br>0.79	dd (11.9, 2.1)<br>t (11.9)
4	43.9	-	-	4	43.95	-	-
5	55.4	1.23	m	5	55.4	0.92	m
6a<br>6b	21.7	1.72<br>1.65	m<br>m	6	21.7	1.69	m
7a<br>7b	41.8	1.50<br>1.34	m<br>m	7a<br>7b	41.8	1.50<br>1.36	m<br>m
8	43.9	-	-	8	43.94	-	-
9	55.2	0.92	d (8.3)	9	55.3	0.92	m
10	40.3	-	-	10	40.3	-	-
11a<br>11b	18.2	1.54<br>1.48	m<br>m	11	18.2	1.51	m
12a<br>12b	25.7	1.52<br>1.32	m<br>m	12a<br>12b	25.8	1.54<br>1.34	m<br>m
13	44.5	1.87	br s	13	44.5	1.87	br s
14a<br>14b	36.8	1.69<br>1.53	m<br>m	14a<br>14b	36.9	1.69<br>1.53	m<br>m
15a<br>15b	52.7	1.39<br>1.25	d (13.9)<br>d (13.9)	15a<br>15b	52.7	1.39<br>1.25	d (15.4)<br>d (15.4)
16	80.4	-	-	16	80.5	-	-
17a<br>17b	65.3	3.50<br>3.40	d (11.0)<br>d (11.0)	17a<br>17b	65.3	3.50<br>3.40	dd (10.8, 4.1)<br>dd (10.8, 4.1)
18	28.5	1.12	s	18	28.5	1.13	s
19	178.5	-	-	19	178.5	-	-
20	16.4	0.85	s	20	16.4	0.86	s
CO_2H	-	-	-	CO_2H	-	12.07	br s
OH	-	-	-	OH	-	3.85	br s
OH	-	-	-	OH	-	4.33	br s
OH	-	-	-	OH	-	4.33	br s

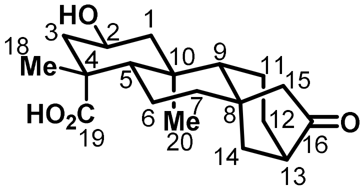

12

Table S3. ^{1}H and ^{13}C NMR chemical shifts for natural and synthetic 2β-hydroxy-16-oxo-*ent*-17-norkauran-19-oic acid (**12**).

Natural Product (400 MHz, C_5D_5N)[9]				Synthetic Product (500 MHz, C_5D_5N)			
Position	δ^{13}C	δ^{1}H	multiplicity (*J* in Hz)	Position	δ^{13}C	δ^{1}H	multiplicity (*J* in Hz)
1a	50.9	2.52	dd (12.3, 2.7)	1a	51.0	2.55	dd (10.8, 2.7)
1b		1.10	s	1b		1.12	t (11.6)
2	64.0	4.81	m	2	64.0	4.84	tt (11.1, 4.5)
3a	48.7	3.07	dd (12.3, 2.7)	3a	48.8	3.10	dd (12.4, 4.1)
3b		1.39	s	3b		1.44	m
4	45.5	-	-	4	45.6	-	-
5	56.7	1.13	s	5	56.7	1.18	br d (12.2)
6a	21.6	2.22	m	6a	21.6	2.23	m
6b		2.04	m	6b		2.08	m
7a	41.6	1.71	m	7a	41.5	1.59	m
7b		1.42	m	7b		1.52	m
8	42.8	-	-	8	42.8	-	-
9	56.7	1.17	-	9	54.3	1.23	m
10	41.4	-	-	10	41.7	-	-
11a	19.4	1.68	m	11a	19.4	1.76	m
11b		1.57	m	11b		1.57	m
12a	29.9	1.93	m	12a	29.9	1.71	m
12b		1.59	m	12b		1.30	m
13	48.3	2.31	m	13	48.3	2.32	br s
14a	37.7	2.10	m	14a	37.7	2.14	dd (12.0, 3.0)
14b		1.27	dd (12.0, 3.8)	14b		1.28	m
15a	55.3	2.00	d (3.8)	15	55.3	2.00	m
15b		1.96	m				
16	221.3	-	-	16	221.1	-	-
18	29.7	1.44	s	18	29.7	1.47	s
19	180.4	-	-	19	180.4	-	-
20	17.9	1.19	s	20	18.0	1.23	s

The chemical shift reported for carbon C9 (red) was probably the result of a typographical error, having been assigned with the same value as carbon C5 (blue) in the original paper. We believe that the correct chemical shift for C9 should be 54.3 ppm.

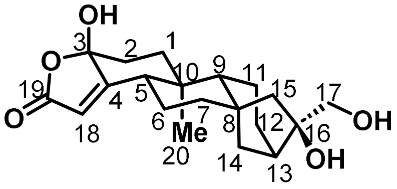

14

Table S4. ^{1}H and ^{13}C NMR chemical shifts for natural and synthetic tricalysiolide B (**14**).

**Natural Product (500 MHz, C_5D_5N)**[10]				**Synthetic Product (500 MHz, C_5D_5N)**			
Position	$\delta^{13}C$	$\delta^{1}H$	multiplicity (*J* in Hz)	Position	$\delta^{13}C$	$\delta^{1}H$	multiplicity (*J* in Hz)
1a	36.1	1.78	m	1a	36.4	1.76	m
1b		1.52	m	1b		1.51	m
2a	35.0	2.52	d (13.7)	2a	35.3	2.53	d (13.8)
2b		2.00	m	2b		1.99	m
3	105.7	-	-	3	106.0	-	-
4	173.7	-	-	4	174.2	-	-
5	47.4	2.57	d (9.7)	5	47.7	2.58	d (9.2)
6a	22.1	1.59	m	6a	22.4	1.56	m
6b		1.38	m	6b		1.39	m
7a	40.3	1.65	m	7a	40.6	1.63	m
7b		1.56	m	7b		1.57	m
8	44.6	-	-	8	44.9	-	-
9	53.7	1.29	d (8.6)	9	54.0	1.30	d (8.5)
10	43.8	-	-	10	44.1	-	-
11a	19.5	1.76	m	11a	19.8	1.74	m
11b		1.52	m	11b		1.54	m
12a	26.4	1.91	m	12a	26.7	1.90	m
12b		1.50	m	12b		1.49	m
13	45.9	2.47	s	13	46.2	2.48	br s
14a	38.1	2.06	dd (11.1, 4.7)	14a	38.4	2.06	m
14b		1.97	d (11.1)	14b		1.97	m
15a	53.8	1.86	d (14.3)	15a	54.1	1.85	d (14.2)
15b		1.77	d (14.3)	15b		1.75	d (14.2)
16	81.5	-	-	16	81.9	-	-
17a	66.4	4.14	dd (10.8, 5.0)	17a	66.7	4.15	d (10.9)
17b		4.06	dd (10.8, 5.0)	17b		4.07	d (10.9)
18	112.6	5.81	s	18	112.8	5.82	s
19	171.5	-	-	19	171.9	-	-
20	14.4	0.80	s	20	14.8	0.80	s
OH-3	-	9.49	s	OH-3	-	-	-
OH-16	-	5.22	s	OH-16	-	-	-
OH-17	-	6.16	br s	OH-17	-	-	-

IV. Crystallographic Data

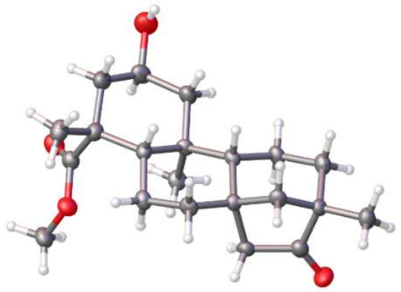

Figure S1: Molecular structure of compound **5** with displacement ellipsoids drawn at the 50% probability level.

Table S5. Crystallographic data for compound **5**.

**Crystal data**	
Chemical formula	$C_{21}H_{32}O_4$
M_r	348.46
Crystal system, space group	Orthorhombic, $P2_12_12_1$
Temperature (K)	120
a, b, c (Å)	7.7074 (11); 8.7014 (13); 27.650 (4)
V (Å^3)	1854.3 (5)
$Z = 4$	Plate, colourless crystal
Radiation type	Mo *K*a radiation, l = 0.71073 Å
m (mm^{-1})	0.08
Crystal size (mm)	0.31 × 0.17 × 0.03

**Data collection**	
Diffractometer	Bruker APEX II Duo, CCD detector diffractometer
Absorption correction	Multi-scan method (SADABS). Bruker (2010). *APEX2*, *SAINT* and *SADABS*. Bruker AXS Inc., Madison, Wisconsin, USA
T_{min}, T_{max}	0.630, 0.745
Number of measured independent reflections, reflections with I > 2s(I), measured reflections	3395, 2662, 17214
R_{int}	0.047
θ_{max}	25.350º

**Refinement**	
$R[F^2 > 2s(F^2)]$, $wR(F^2)$, S	0.063, 0.158, 1.05
Number of reflections	3395
Number of parameters	231
Number of restraints	0
H-atom treatment	H-atom parameters constrained
$D\rho_{max}$, $D\rho_{min}$ (e $Å^{-3}$)	0.49, -0.30

Computing details

Data collection strategy consisted of sets of *phi* and *omega* scans (*APEX2)*.[11] Data reduction and cell refinement were performed by *SAINT*, while *SADABS* was used for absorption correction by multi-scan method.[11] Crystal structure was solved by direct methods with *SHELXS97*[12] and refined by free-matrix least square on F^2 by SHELXL2014/7.[13]

V. References

[1] Williams, D. B. G.; Lawton, M. Drying of Organic Solvents: Quantitative Evaluation of the Efficiency of Several Desiccants. *J. Org. Chem.* **2010**, *75* (24), 8351–8354. [Link]

[2] Still, W. C.; Kahn, M.; Mitra, A. Rapid Chromatographic Technique for Preparative Separations with Moderate Resolution. *J. Org. Chem.* **1978**, *43* (14), 2923–2925. [Link]

[3] Chang, S.-F.; Yang, L.-M.; Hsu, F.-L.; Hsu, J.-Y.; Liaw, J.-H.; Lin, S.-J. Transformation of Steviol-16α,17-Epoxide by *Streptomyces griseus* and *Cunninghamella bainieri*. *J. Nat. Prod.* **2006**, *69* (10), 1450–1455. [Link]

[4] Chen, P.; Zhang, D.; Li, M.; Wu, Q.; Lam, Y. P. Y.; Guo, Y.; Chen, C.; Bai, N.; Malhotra, S.; Li, W.; O'Connor, P. B.; Fu, H. Discovery of Novel, Potent, Isosteviol-Based Antithrombotic Agents. *Eur. J. Med. Chem.* **2019**, *183*, 111722. [Link]

[5] Hutt, O. E.; Doan, T. L.; Georg, G. I. Synthesis of Skeletally Diverse and Stereochemically Complex Library Templates Derived from Isosteviol and Steviol. *Org. Lett.* **2013**, *15* (7), 1602–1605. [Link]

[6] Ferreira, N.; Ribeiro, A.; Morais, M.; Peixoto, A.; Bernardino, M.; Rodrigues, M.; Soares, A. C.; Heleno, V.; Veneziani, R.; Tavares, D. Cytotoxic and Genotoxic Effects of the *ent*-Kaurenoic Acid and *ent*-Kaurenoic Acid Enriched *Mikania glomerata* Extract in V79. *J. Nat. Prod. Biochem*. 2020, 18 (1), 1–4. [Link]

[7] Lima, F. A.; Bezerra, M. A. M.; Souza, R.; Itabaiana, I.; Haynes, T.; Hermans, S.; Wojcieszak, R.; Novaes, F. J. M.; Rezende, C. M. Fast and Highly Selective Continuous-Flow Catalytic Hydrogenation of a Cafestol–Kahweol Mixture Obtained from Green Coffee Beans. *ACS Omega* **2020**, *5* (40), 25712–25722. [Link]

[8] Ohkoshi, E.; Kamo, S.; Makino, M.; Fujimoto, Y. *ent*-Kaurenoic Acids from *Mikania hirsutissima* (Compositae). *Phytochemistry* **2004**, *65* (7), 885–890. [Link]

[9] Rocha, A. D.; Santos, G. C. D.; Fernandes, N. G.; Pfenning, L. H.; Takahashi, J. A.; Boaventura, M. A. D. Hydroxylation at Carbon-2 of *ent*-16-Oxo-17-Norkauran-19-Oic Acid by *Fusarium proliferatum*. *J. Nat. Prod.* **2010**, *73* (8), 1431–1433. [Link]

[10] Nishimura, K.; Hitotsuyanagi, Y.; Sugeta, N.; Sakakura, K.; Fujita, K.; Fukaya, H.; Aoyagi, Y.; Hasuda, T.; Kinoshita, T.; He, D.-H.; Otsuka, H.; Takeda, Y.; Takeya, K. Tricalysiolides A–F, New Rearranged *ent*-Kaurane Diterpenes from *Tricalysia dubia*. *Tetrahedron* **2006**, *62* (7), 1512–1519. [Link]

[11] Bruker; *APEX2*, *SAINT* and *SADABS*. Bruker AXS Inc., Madison, Wisconsin, USA, 2010.

[12] (a) Sheldrick, G. M.; *SHELXS-97, Program for Crystal Structure Resolution*, University of Göttingen, Göttingen, Germany, 1997. (b) Sheldrick, G. M. A Short History of *SHELX*. *Acta Crystallogr. A Found. Crystallogr.* **2008**, *64* (1), 112–122. [Link]

[13] (a) Sheldrick, G. M.; *SHELXL-2014, Program for Structure Refinement*, University of Göttingen, Göttingen, Germany, 2014. (b) (1) Sheldrick, G. M. Crystal Structure Refinement with *SHELXL*. *Acta Crystallogr. C Struct. Chem.* **2015**, *71* (1), 3–8. [Link]

VI. Spectral Data

^{1}H NMR of compound 4:

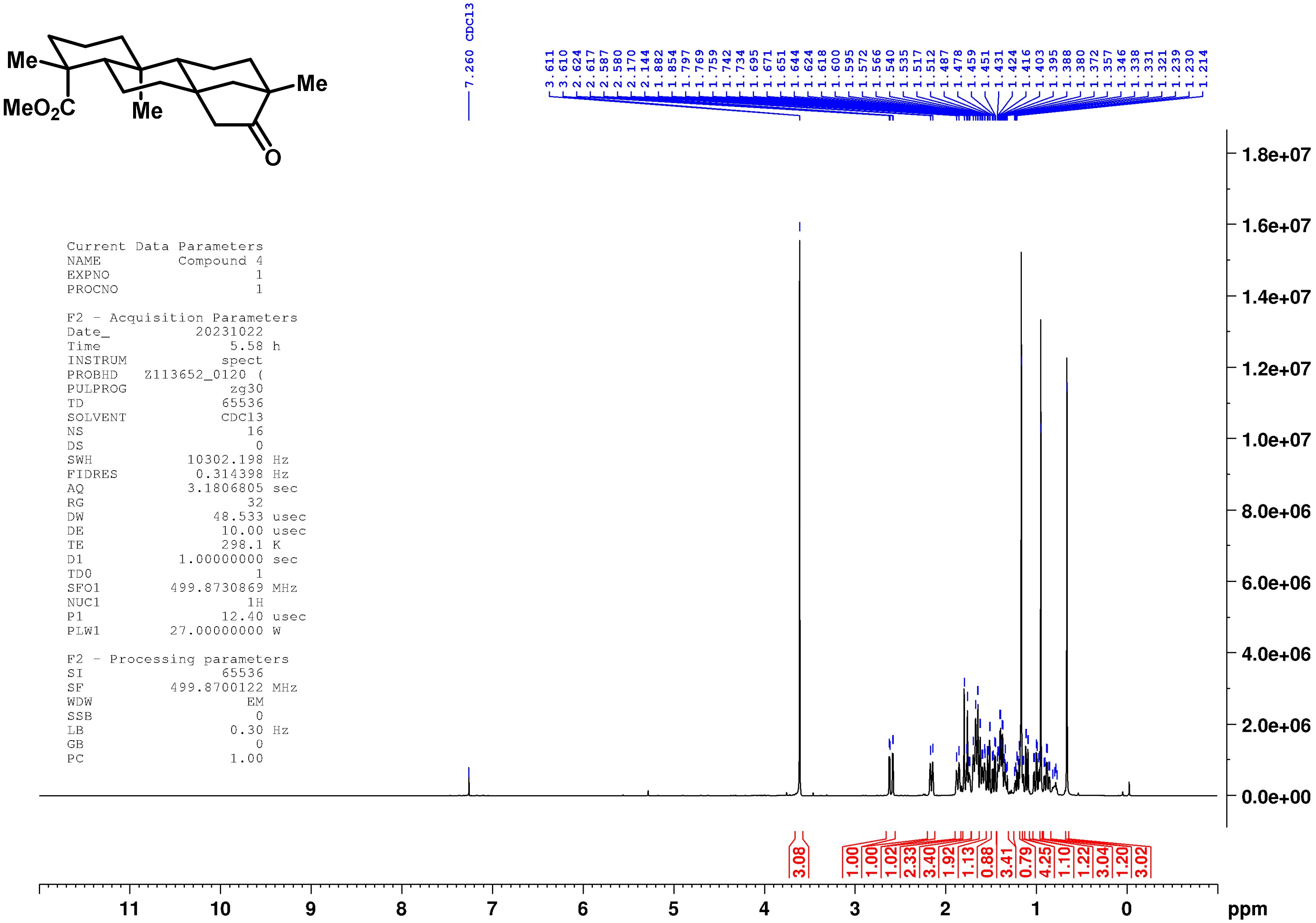

^{13}C NMR of compound 4:

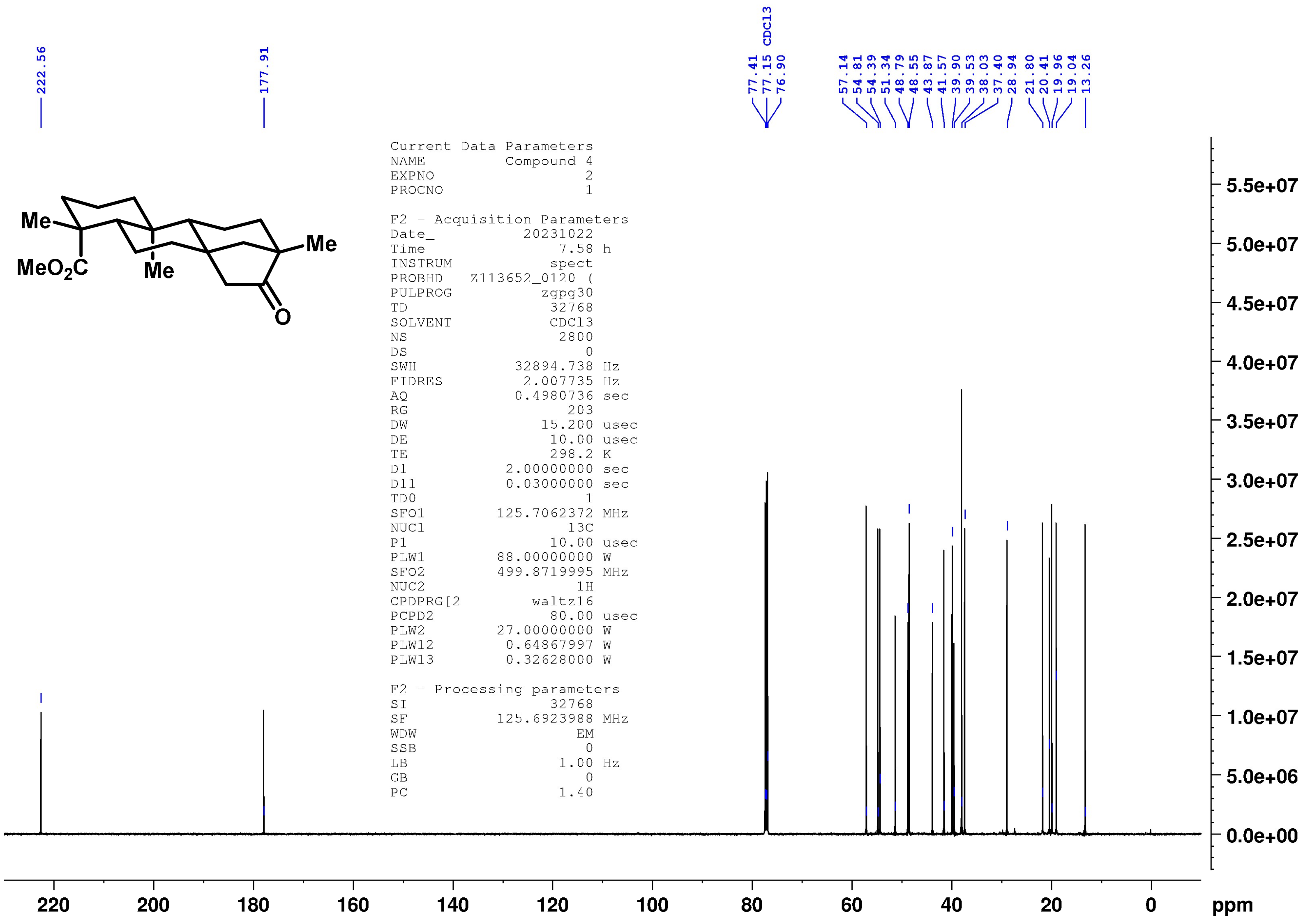

^{1}H NMR of compound 5:

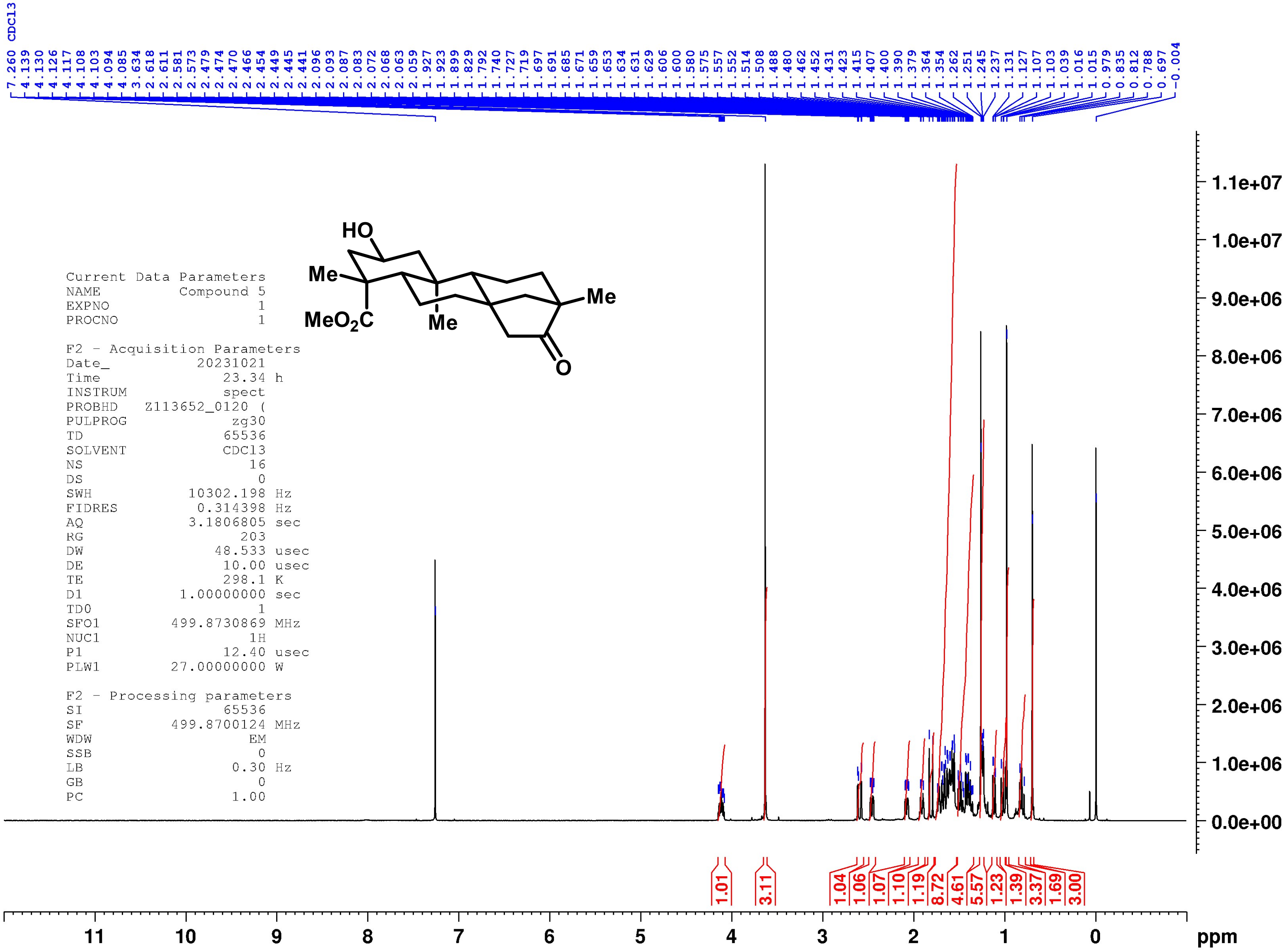

^{13}C NMR of compound 5:

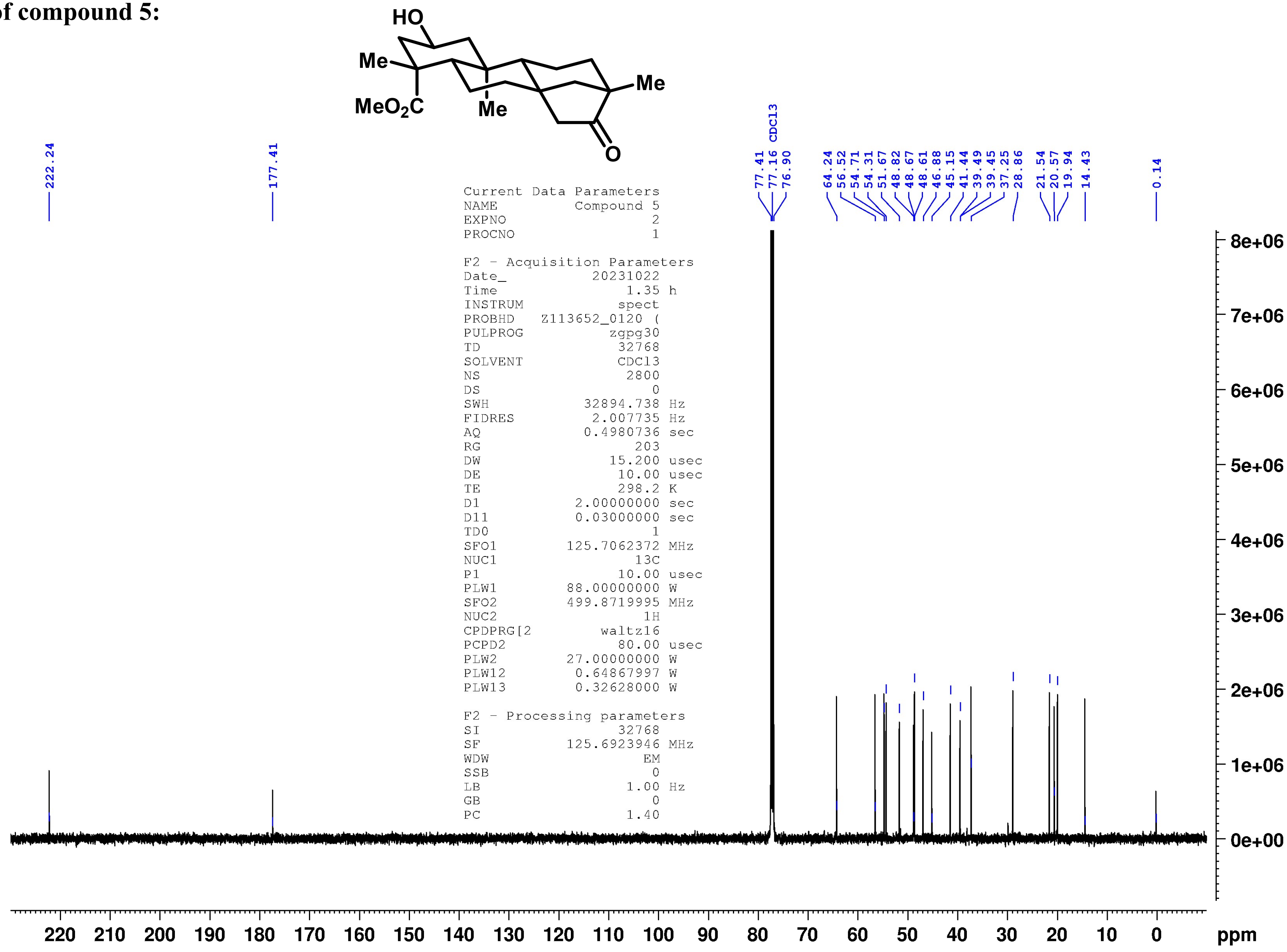

^{1}H NMR of compound 6:

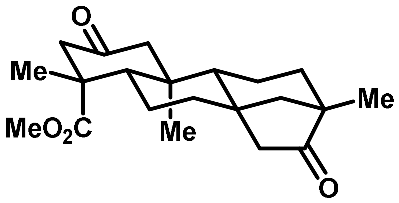

Current Data Parameters
NAME Compound 6
EXPNO 1
PROCNO 1

F2 - Acquisition Parameters
Date_ 20231022
Time 3.50 h
INSTRUM spect
PROBHD Z113652_0120 (
PULPROG zg30
TD 65536
SOLVENT CDCl3
NS 16
DS 0
SWH 10302.198 Hz
FIDRES 0.314398 Hz
AQ 3.1806805 sec
RG 128
DW 48.533 usec
DE 10.00 usec
TE 298.1 K
D1 1.00000000 sec
TD0 1
SFO1 499.8730869 MHz
NUC1 1H
P1 12.40 usec
PLW1 27.00000000 W

F2 - Processing parameters
SI 65536
SF 499.8700123 MHz
WDW EM
SSB 0
LB 0.30 Hz
GB 0
PC 1.00

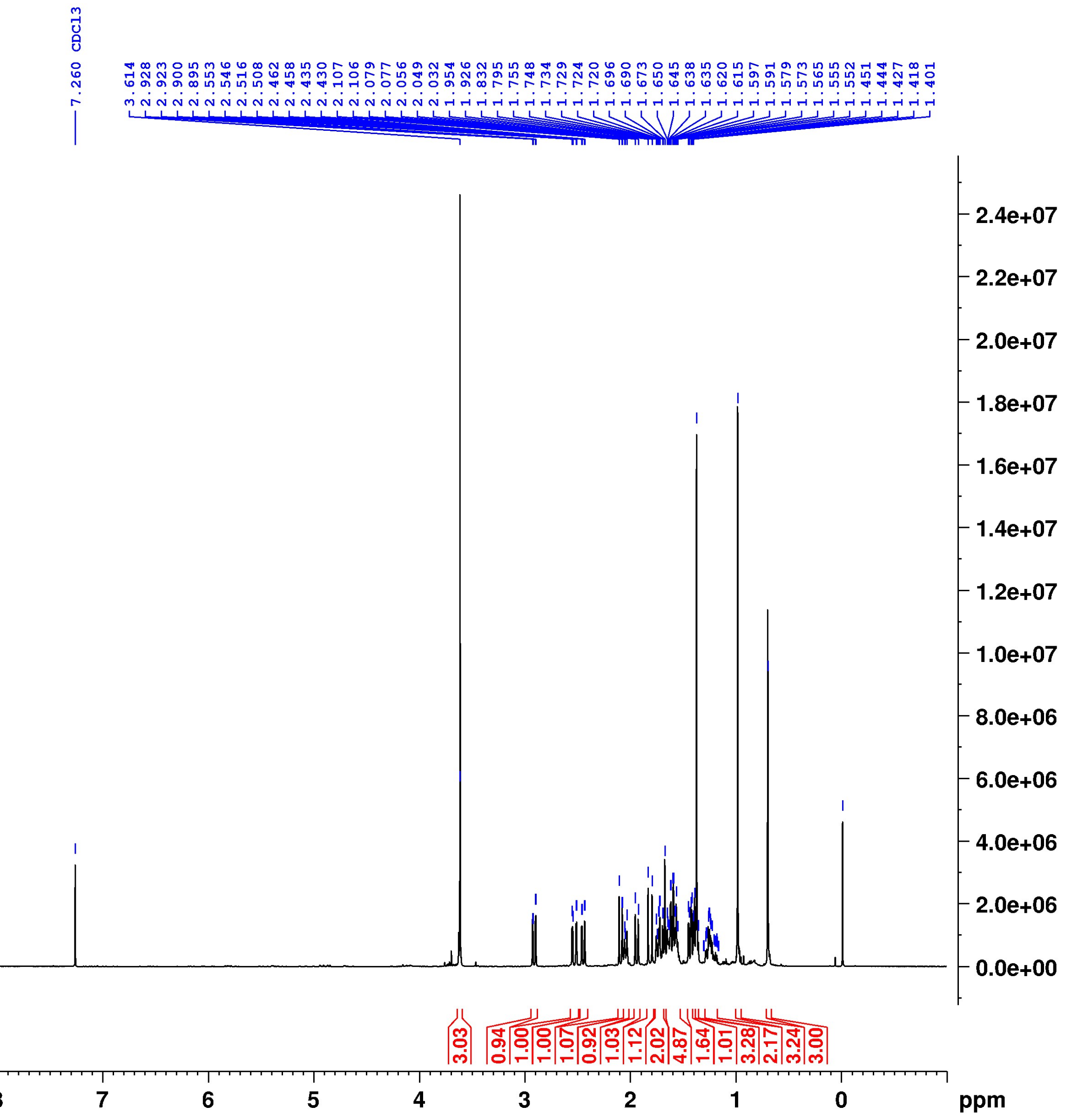

^{13}C NMR of compound 6:

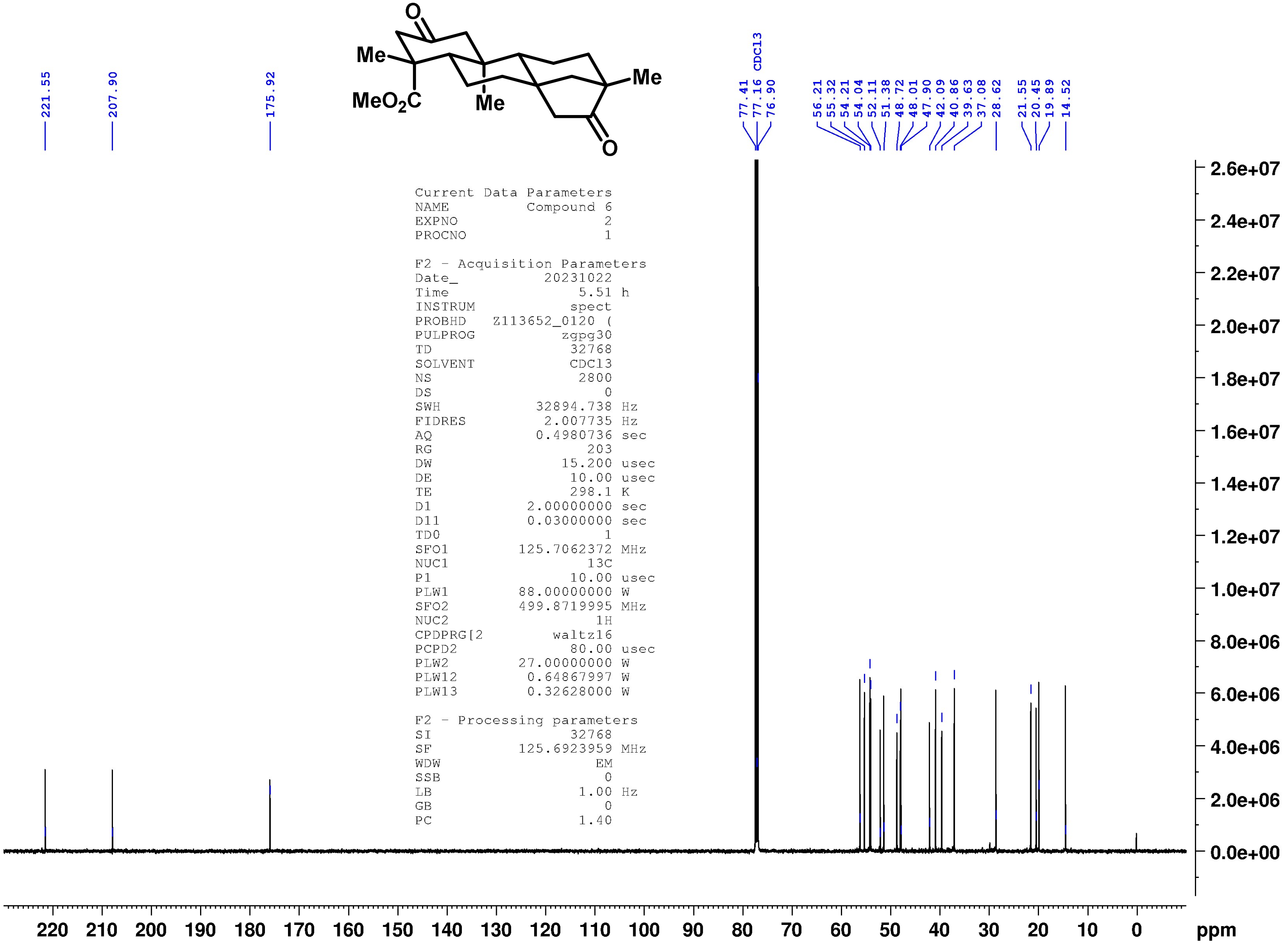


```
Current Data Parameters
NAME           Compound 6
EXPNO                   2
PROCNO                  1

F2 - Acquisition Parameters
Date_            20231022
Time                 5.51 h
INSTRUM             spect
PROBHD   Z113652_0120 (
PULPROG           zgpg30
TD                 32768
SOLVENT            CDCl3
NS                  2800
DS                     0
SWH            32894.738 Hz
FIDRES          2.007735 Hz
AQ             0.4980736 sec
RG                   203
DW                15.200 usec
DE                 10.00 usec
TE                 298.1 K
D1            2.00000000 sec
D11           0.03000000 sec
TD0                    1
SFO1        125.7062372 MHz
NUC1                 13C
P1                 10.00 usec
PLW1         88.00000000 W
SFO2         499.8719995 MHz
NUC2                  1H
CPDPRG[2         waltz16
PCPD2              80.00 usec
PLW2         27.00000000 W
PLW12         0.64867997 W
PLW13         0.32628000 W

F2 - Processing parameters
SI                 32768
SF           125.6923959 MHz
WDW                   EM
SSB                    0
LB                  1.00 Hz
GB                     0
PC                  1.40
```

^{1}H NMR of compound 7:

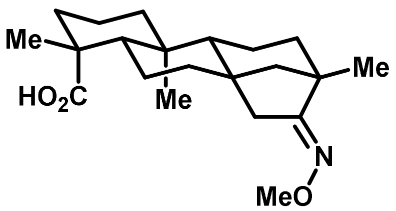


```
Current Data Parameters
NAME          Compound 7
EXPNO                  1
PROCNO                 1

F2 - Acquisition Parameters
Date_           20250411
Time               17.44 h
INSTRUM            spect
PROBHD   Z113652_0120 (
PULPROG             zg30
TD                 65536
SOLVENT            CDCl3
NS                    16
DS                     0
SWH            10302.198 Hz
FIDRES          0.314398 Hz
AQ             3.1806805 sec
RG                   128
DW                48.533 usec
DE                 10.00 usec
TE                 298.2 K
D1            1.00000000 sec
TD0                    1
SFO1         499.8730869 MHz
NUC1                  1H
P1                 12.40 usec
PLW1         27.00000000 W

F2 - Processing parameters
SI                 65536
SF           499.8700124 MHz
WDW                   EM
SSB                    0
LB                  0.30 Hz
GB                     0
PC                  1.00
```

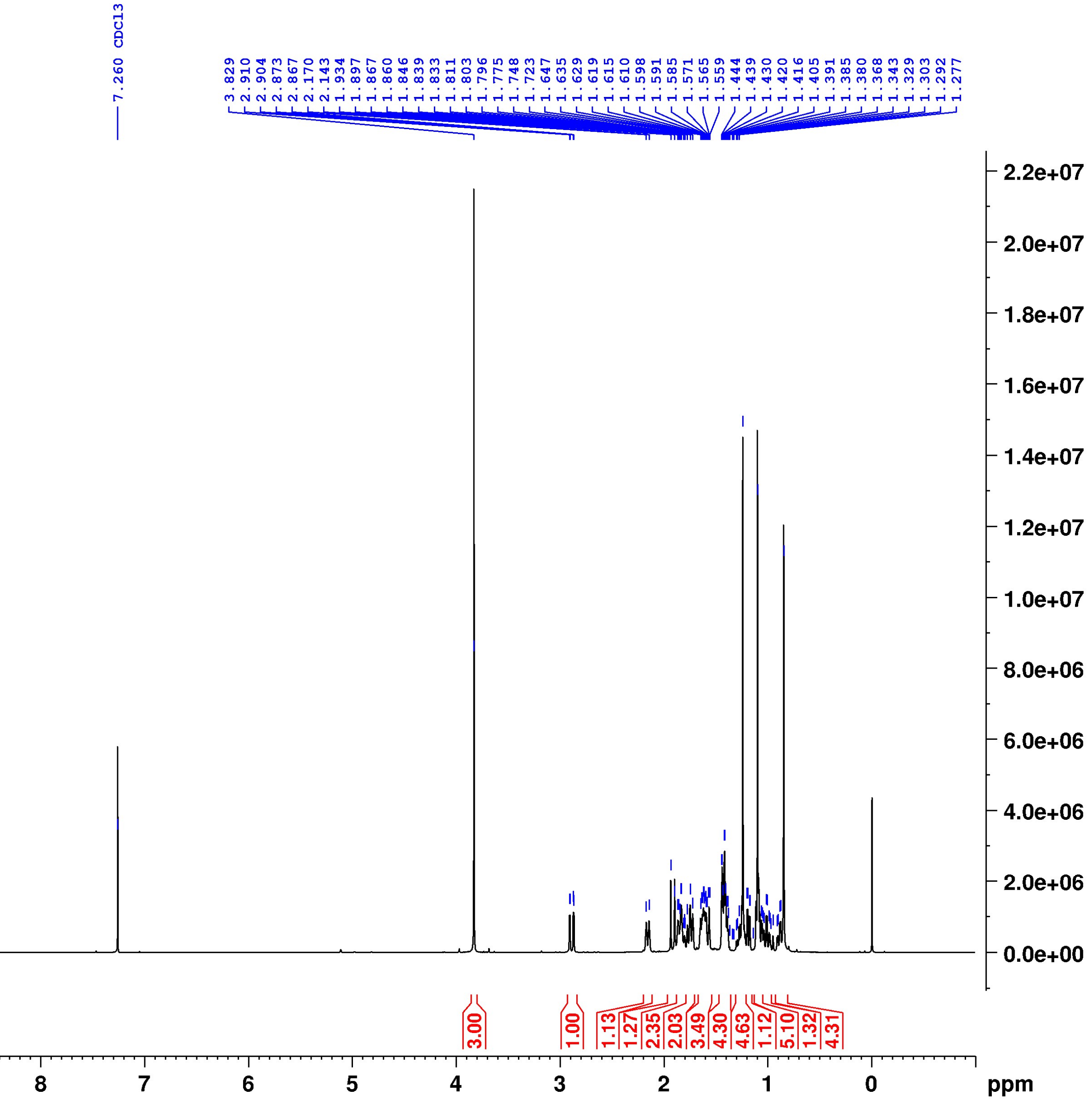

^{13}C NMR of compound 7:

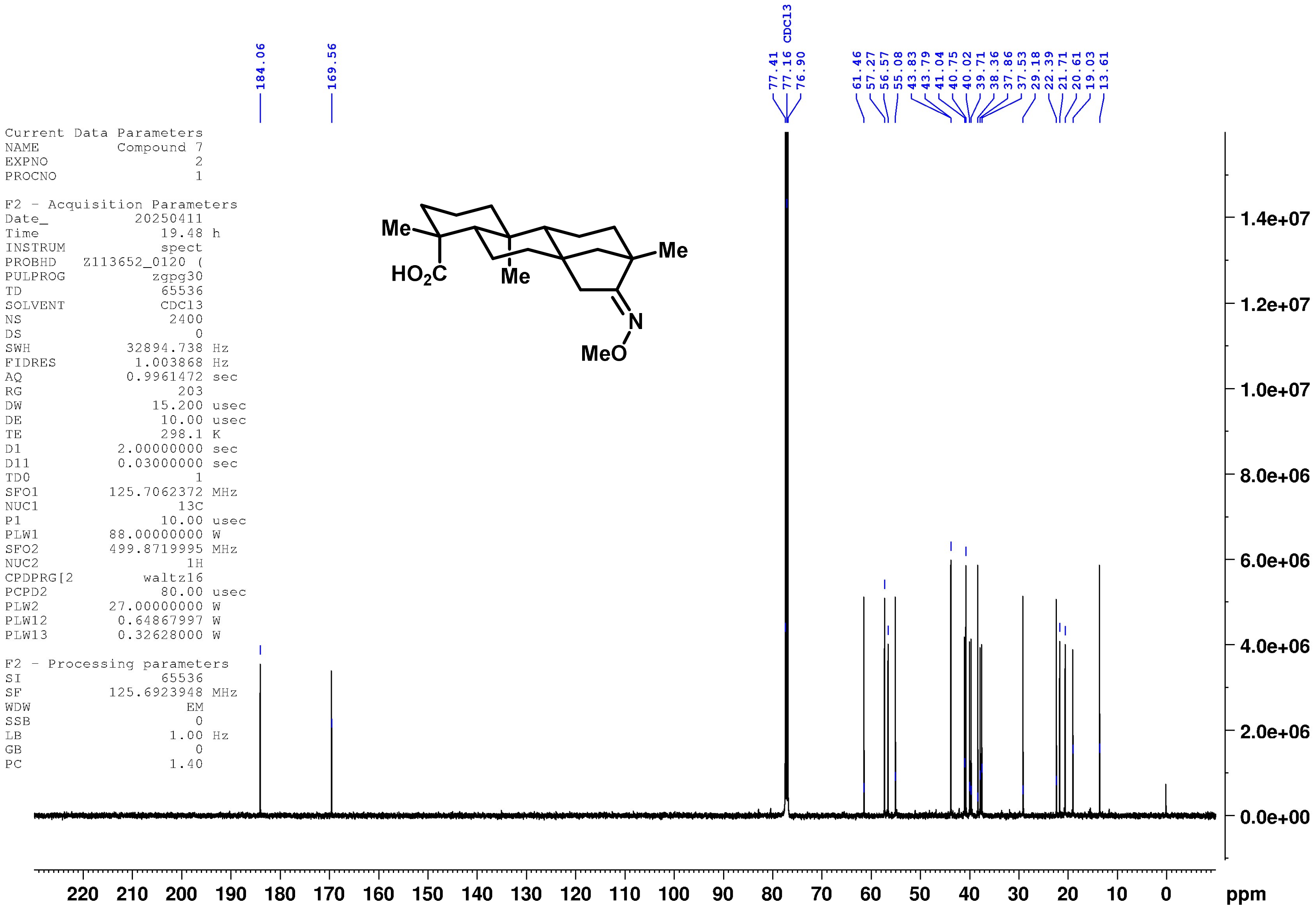

^{1}H NMR of compound 8:

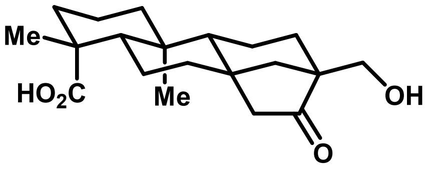

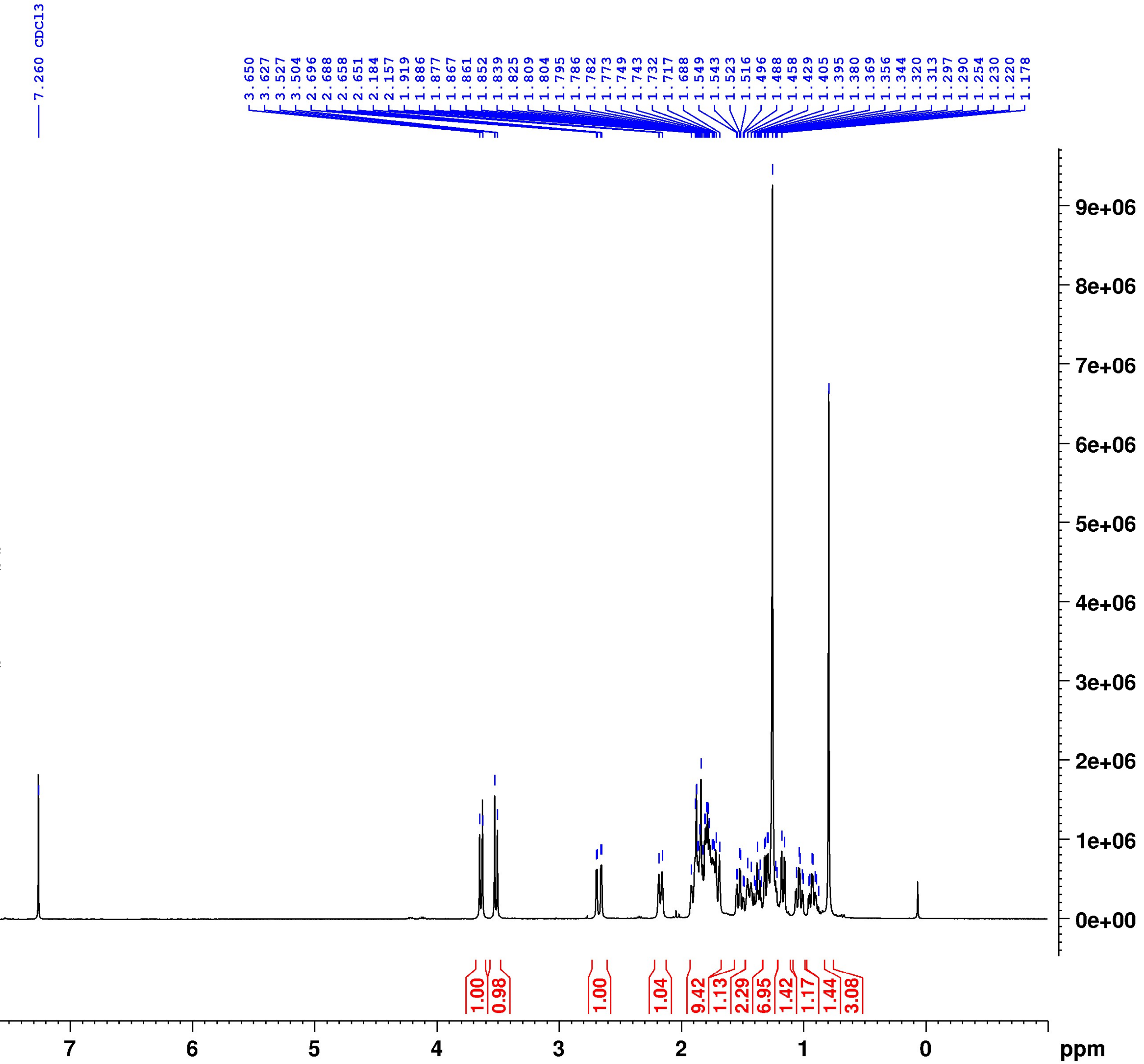

^{13}C NMR of compound 8:

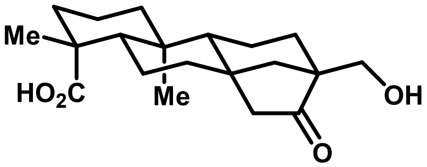

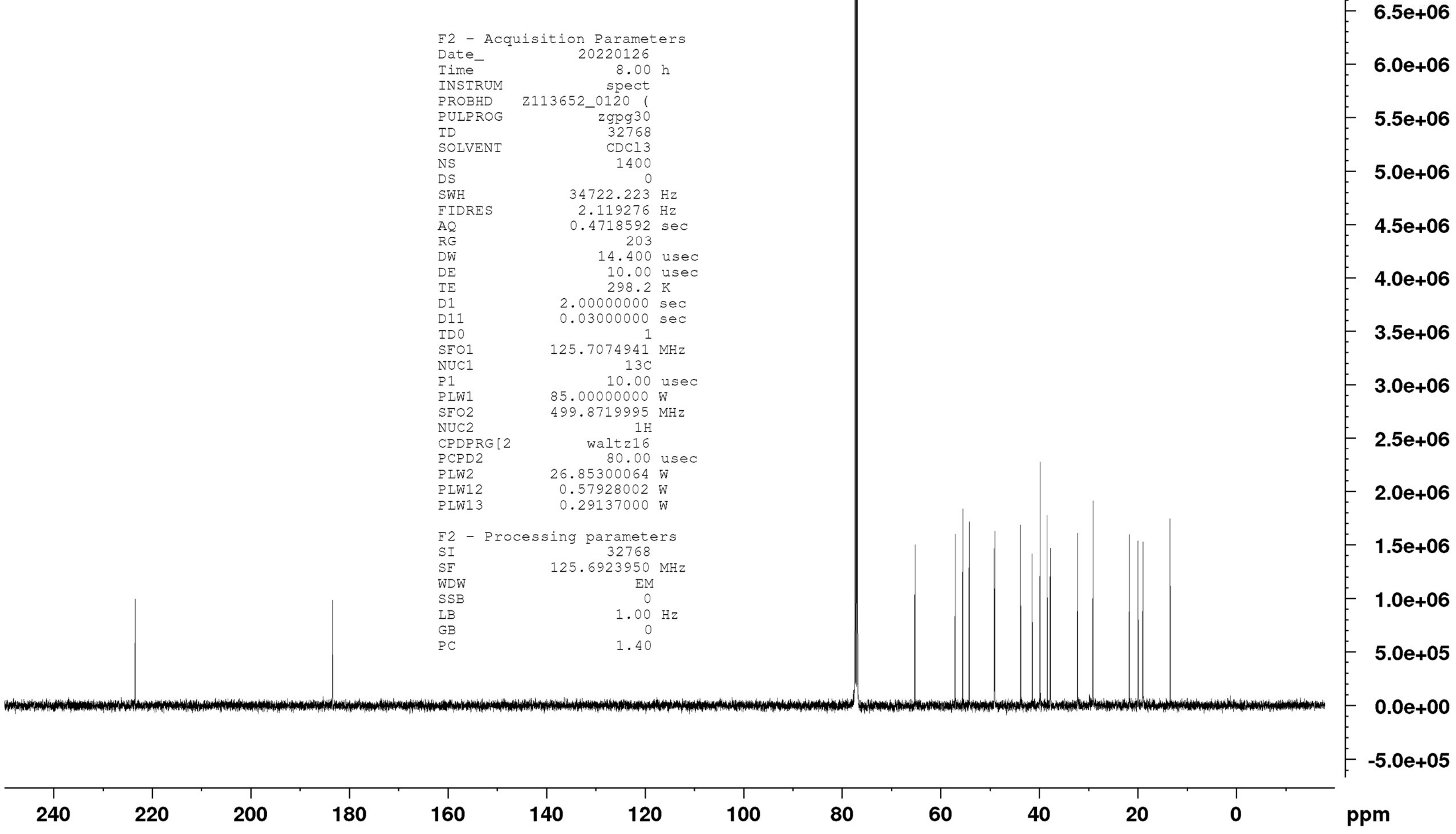

^{1}H NMR of compound 3:

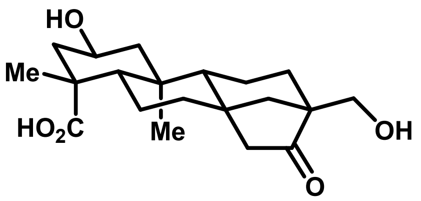

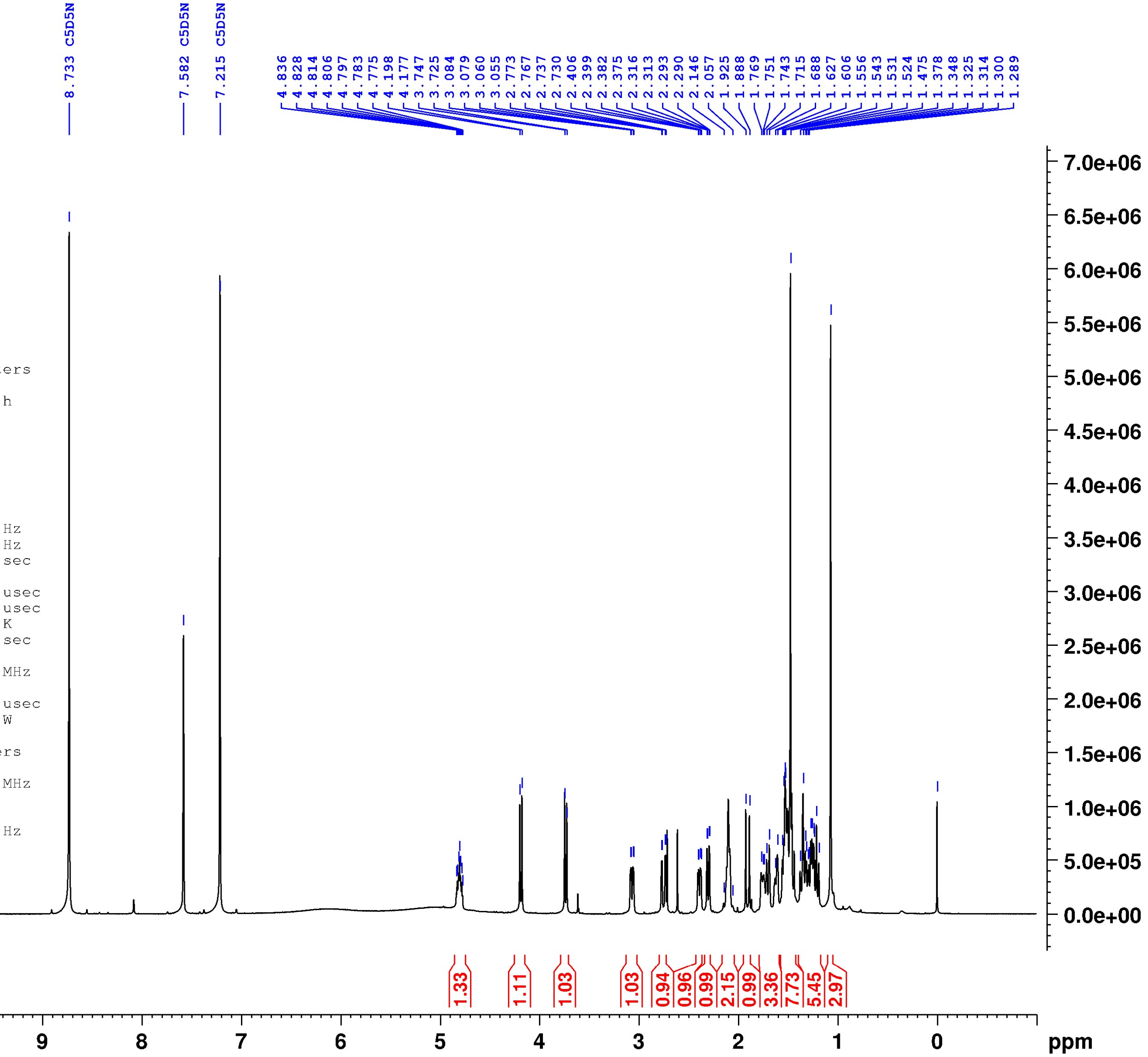

^{13}C NMR of compound 3:

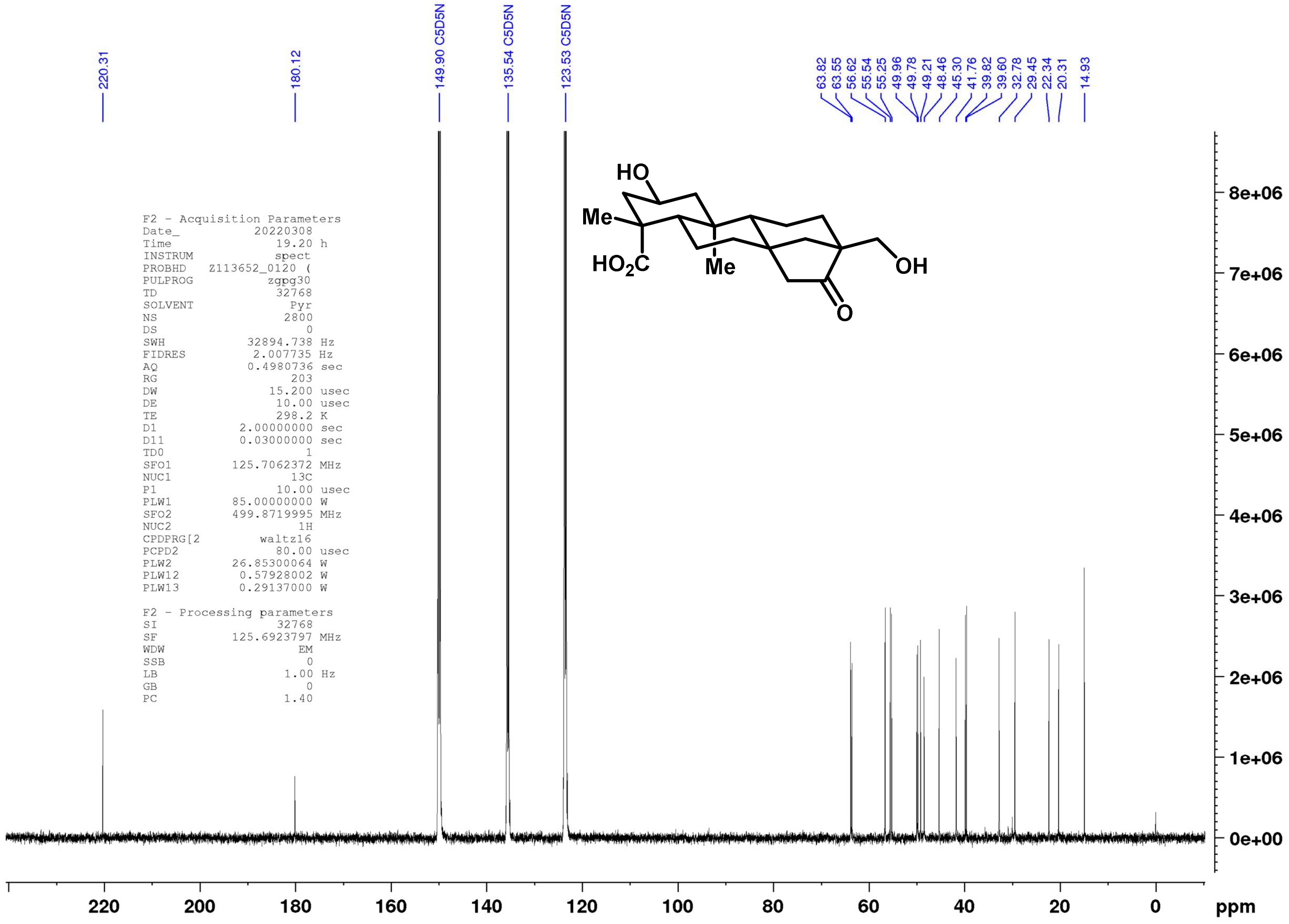

^{1}H NMR of compound 9:

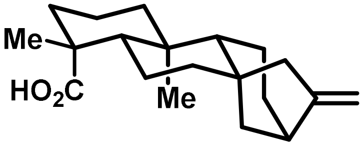

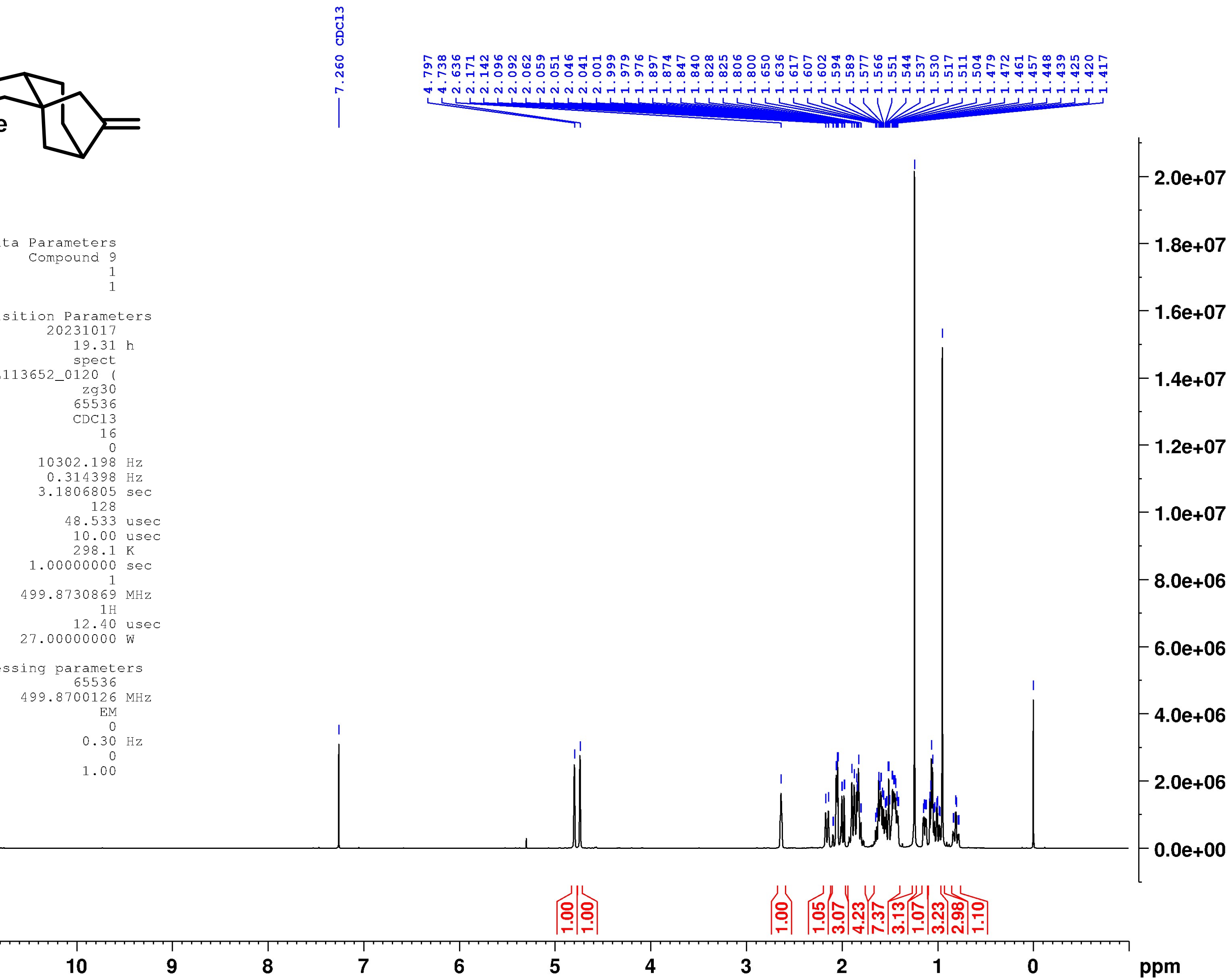

^{13}C NMR of compound 9:

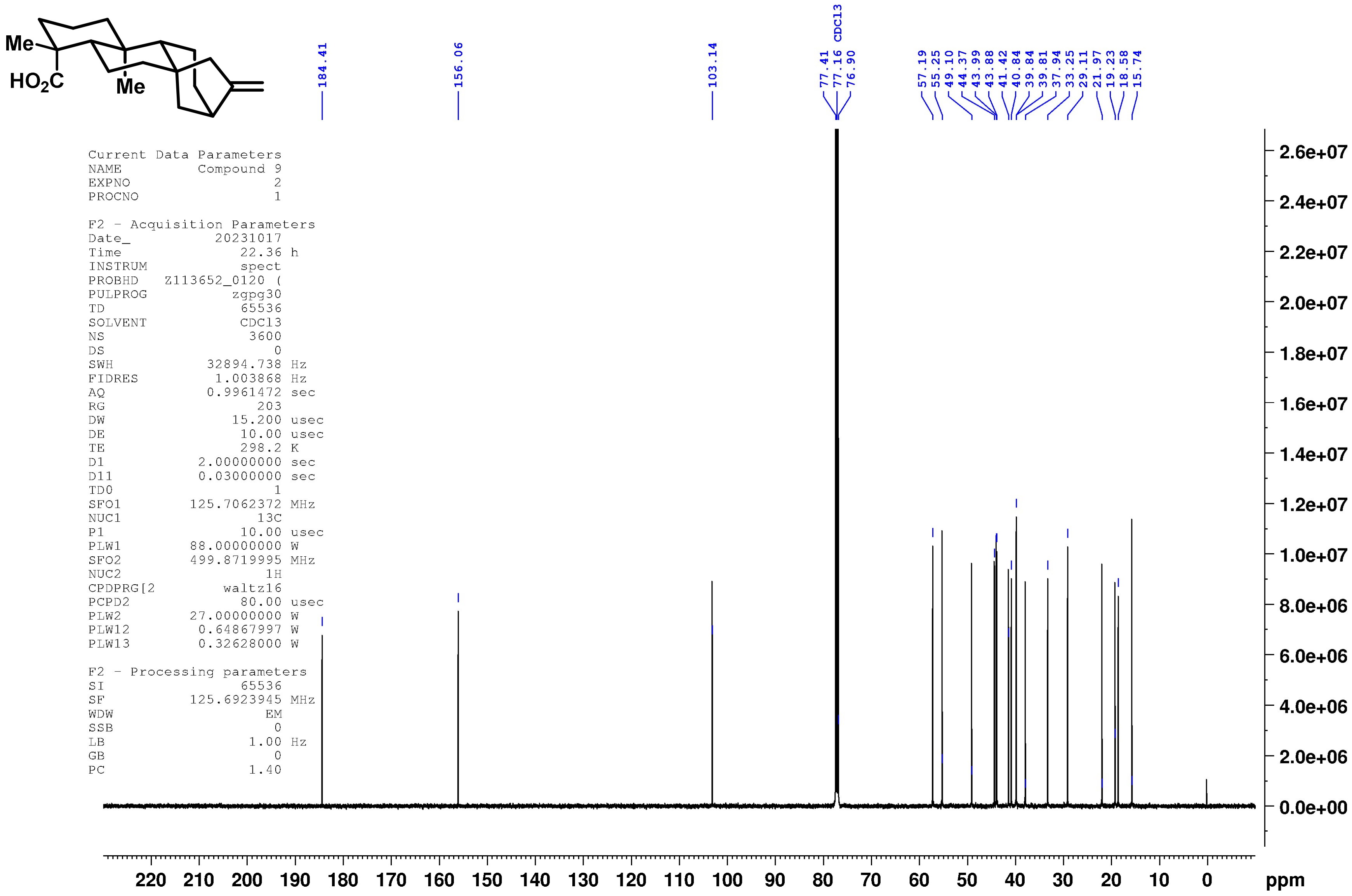

^{1}H NMR of compound 10:

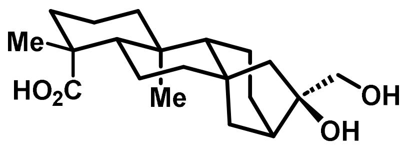

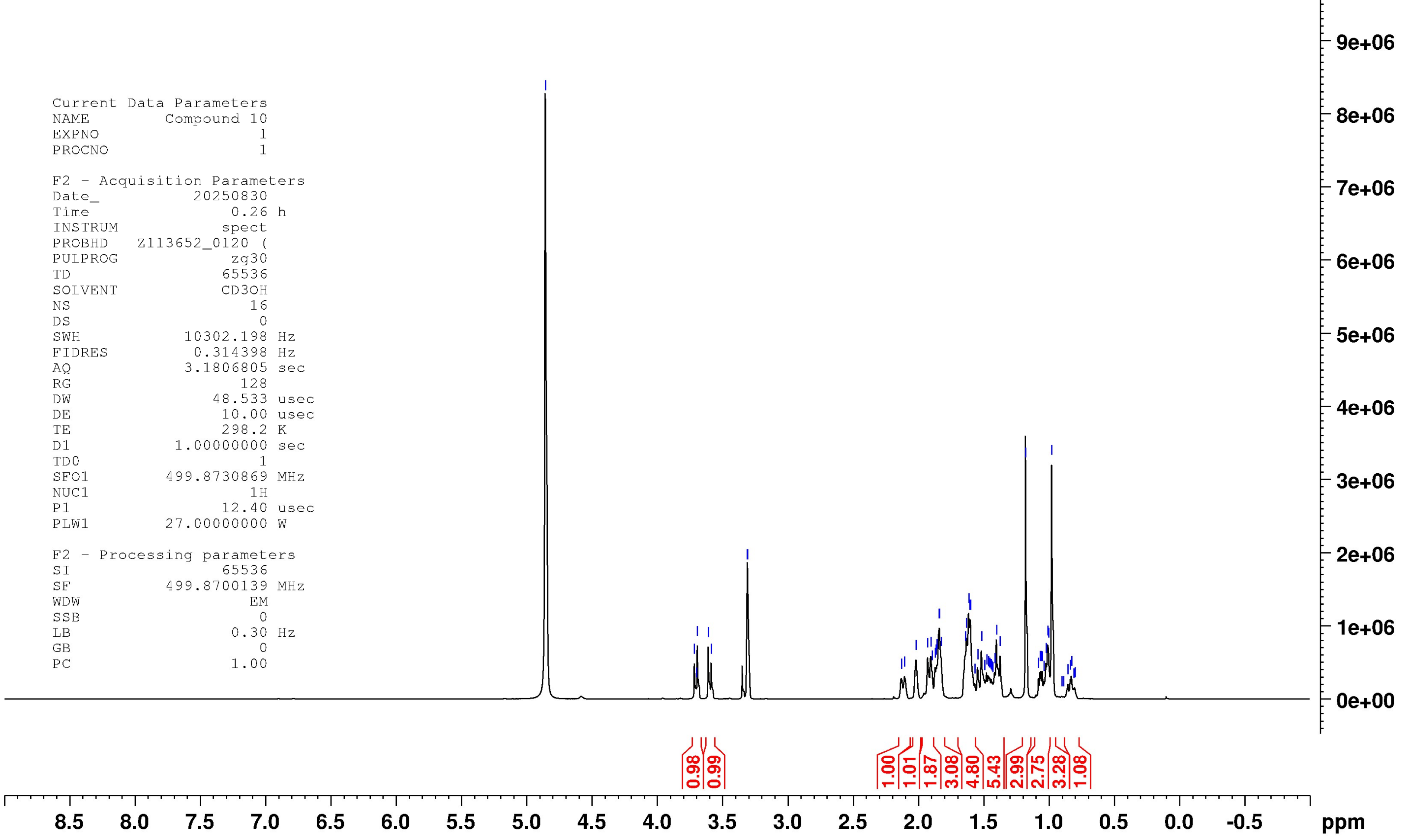

^{13}C NMR of compound 10:

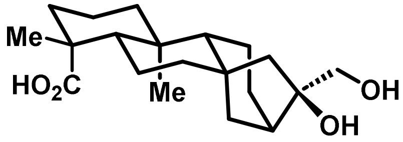

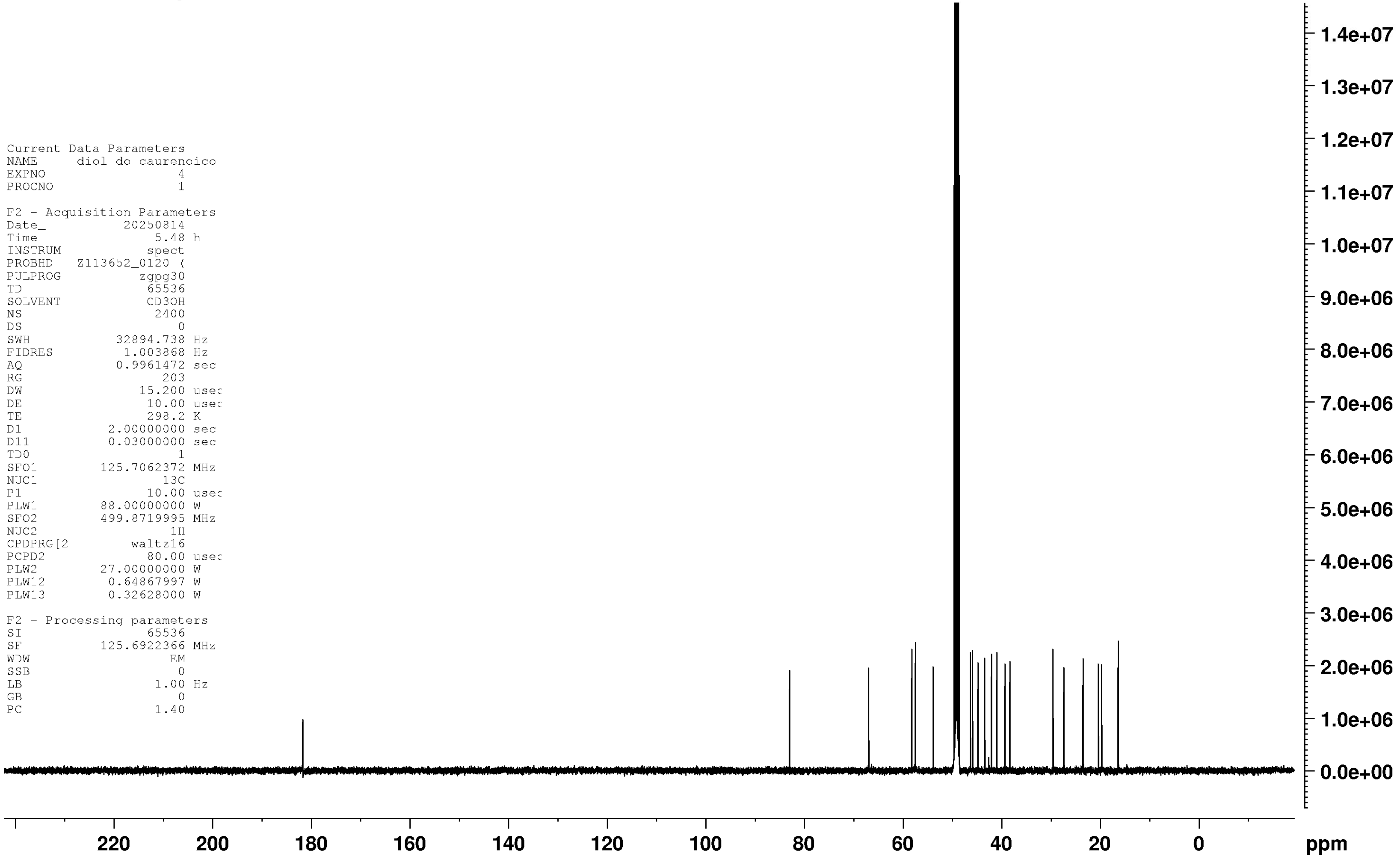

COSY of compound 10:

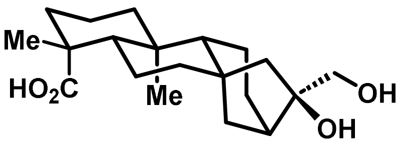

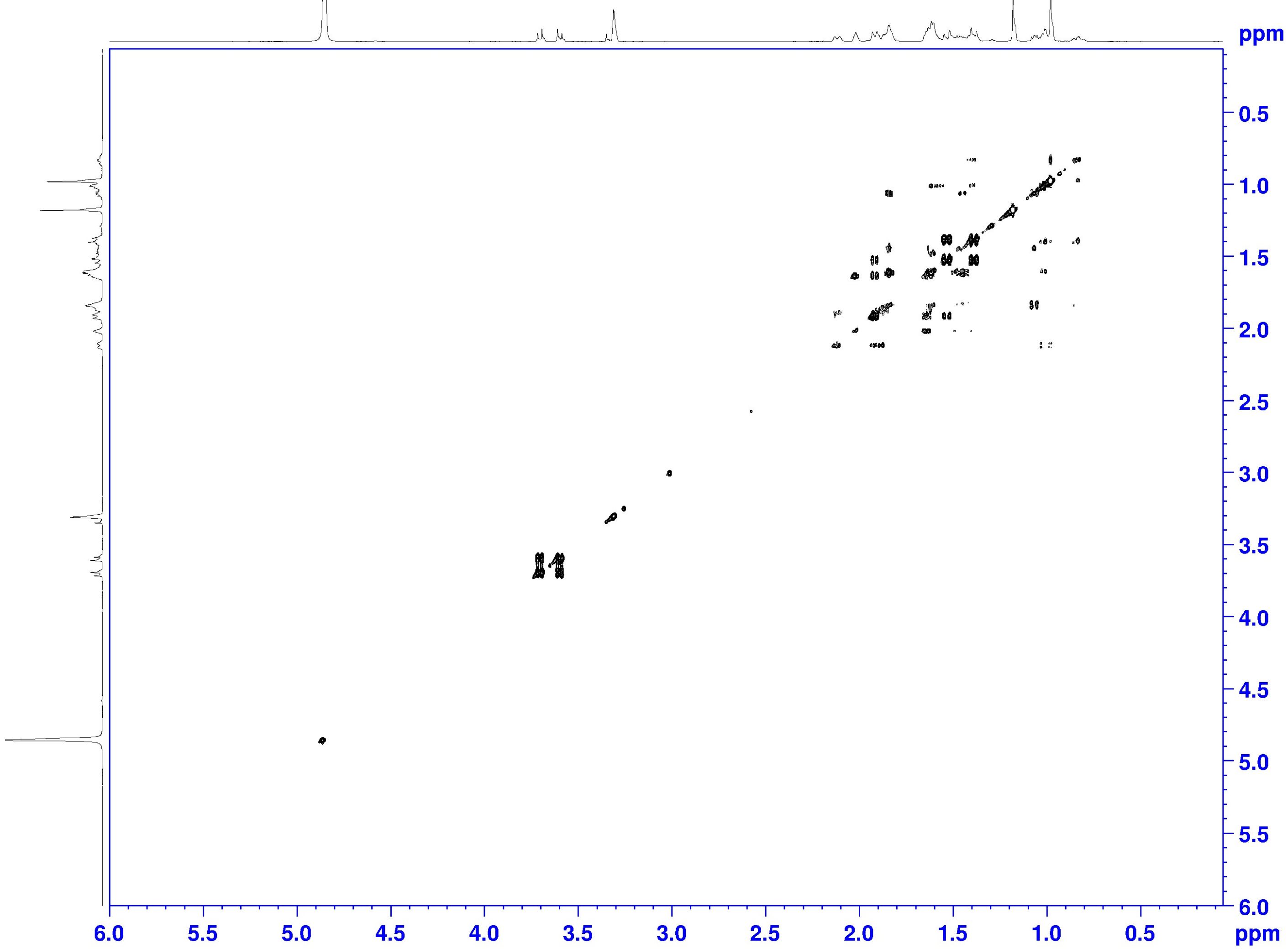


```
Current Data Parameters
NAME      diol do caurenoico
EXPNO                    2
PROCNO                   1

F2 - Acquisition Parameters
Date_           20250814
Time                1.49 h
INSTRUM            spect
PROBHD   Z113652_0120 (
PULPROG       cosygpppqf
TD                  2048
SOLVENT            CD3OH
NS                     4
DS                    16
SWH             2762.431 Hz
FIDRES          2.697686 Hz
AQ             0.3706880 sec
RG                    64
DW               181.000 usec
DE                 10.00 usec
TE                 298.1 K
D0            0.00000300 sec
D1            1.78700805 sec
D11           0.03000000 sec
D12           0.00002000 sec
D13           0.00000400 sec
D16           0.00020000 sec
IN0           0.00036200 sec
TDav                   1
SFO1         499.8714267 MHz
NUC1                  1H
P0                 12.40 usec
P1                 12.40 usec
P17              2500.00 usec
PLW1         27.00000000 W
PLW10         4.61280012 W
GPNAM[1]      SMSQ10.100
GPZ1               10.00 %
P16              1000.00 usec

F1 - Acquisition parameters
TD                   256
SFO1            499.8714 MHz
FIDRES         21.581491 Hz
SW                 5.526 ppm
FnMODE                QF

F2 - Processing parameters
SI                  4096
SF           499.8700148 MHz
WDW                QSINE
SSB                    0
LB                     0 Hz
GB                     0
PC                  1.40

F1 - Processing parameters
SI                  1024
MC2                   QF
SF           499.8700152 MHz
WDW                QSINE
SSB                    0
LB                     0 Hz
GB                     0
```

NOESY of compound 10:

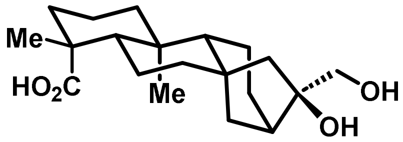

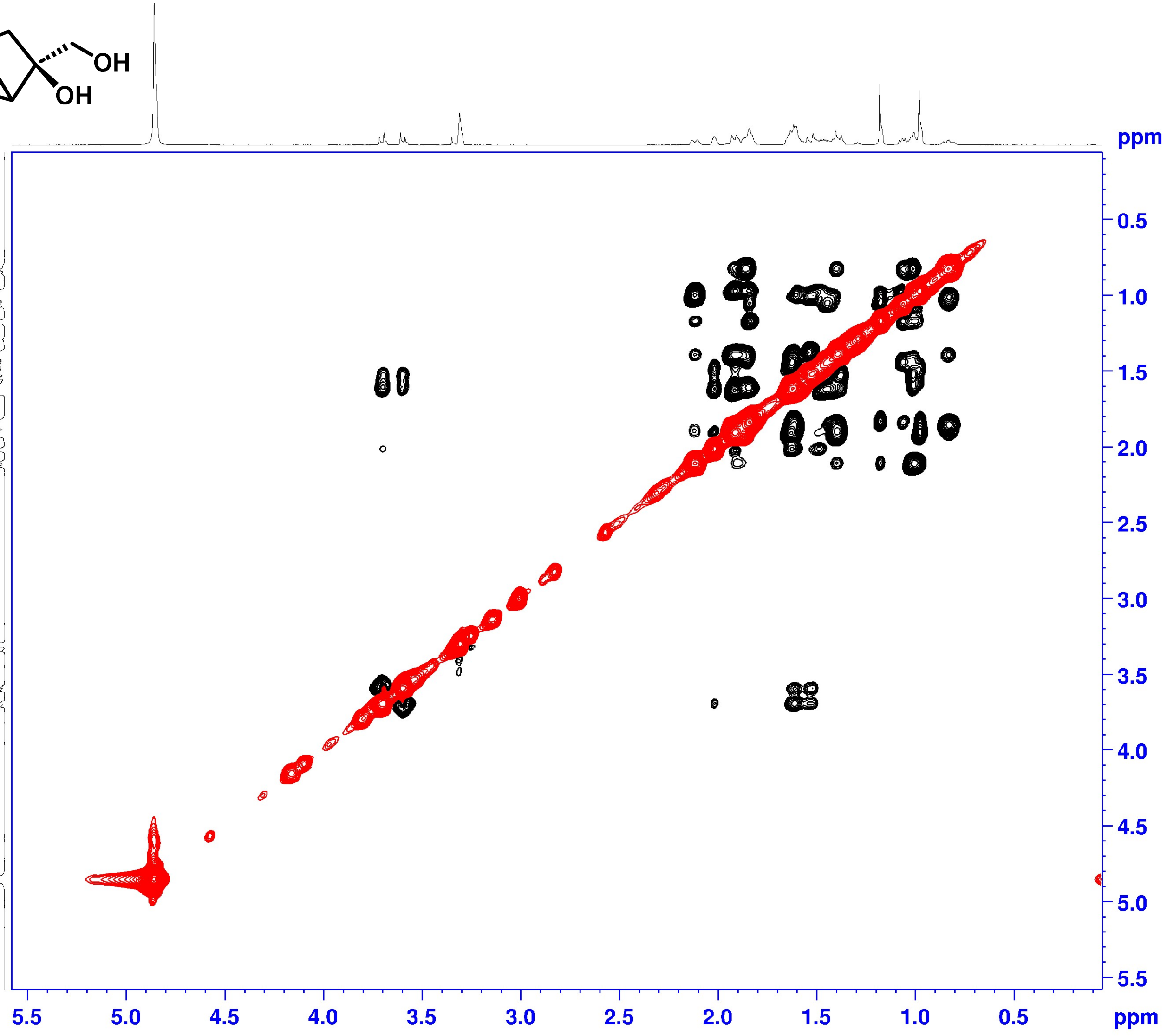

Current Data Parameters
NAME diol do caurenoico
EXPNO 3
PROCNO 1

F2 - Acquisition Parameters
Date_ 20250814
Time 2.29 h
INSTRUM spect
PROBHD Z113652_0120 (
PULPROG noesygpph
TD 2048
SOLVENT CD3OH
NS 6
DS 16
SWH 2762.431 Hz
FIDRES 2.697686 Hz
AQ 0.3706880 sec
RG 57
DW 181.000 usec
DE 10.00 usec
TE 298.2 K
D0 0.00016521 sec
D1 1.78700805 sec
D8 0.80000001 sec
D16 0.00020000 sec
IN0 0.00036200 sec
TDav 1
SFO1 499.8714267 MHz
NUC1 1H
P1 12.40 usec
P2 24.80 usec
PLW1 27.00000000 W
GPNAM[1] SMSQ10.100
GPZ1 40.00 %
P16 1000.00 usec

F1 - Acquisition parameters
TD 256
SFO1 499.8714 MHz
FIDRES 21.581491 Hz
SW 5.526 ppm
FnMODE TPPI

F2 - Processing parameters
SI 4096
SF 499.8700182 MHz
WDW GM
SSB 0
LB -1.00 Hz
GB 0.005
PC 1.00

F1 - Processing parameters
SI 1024
MC2 TPPI
SF 499.8700183 MHz
WDW GM
SSB 0
LB -1.00 Hz
GB 0.01

HSQC of compound 10:

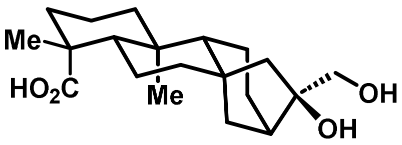

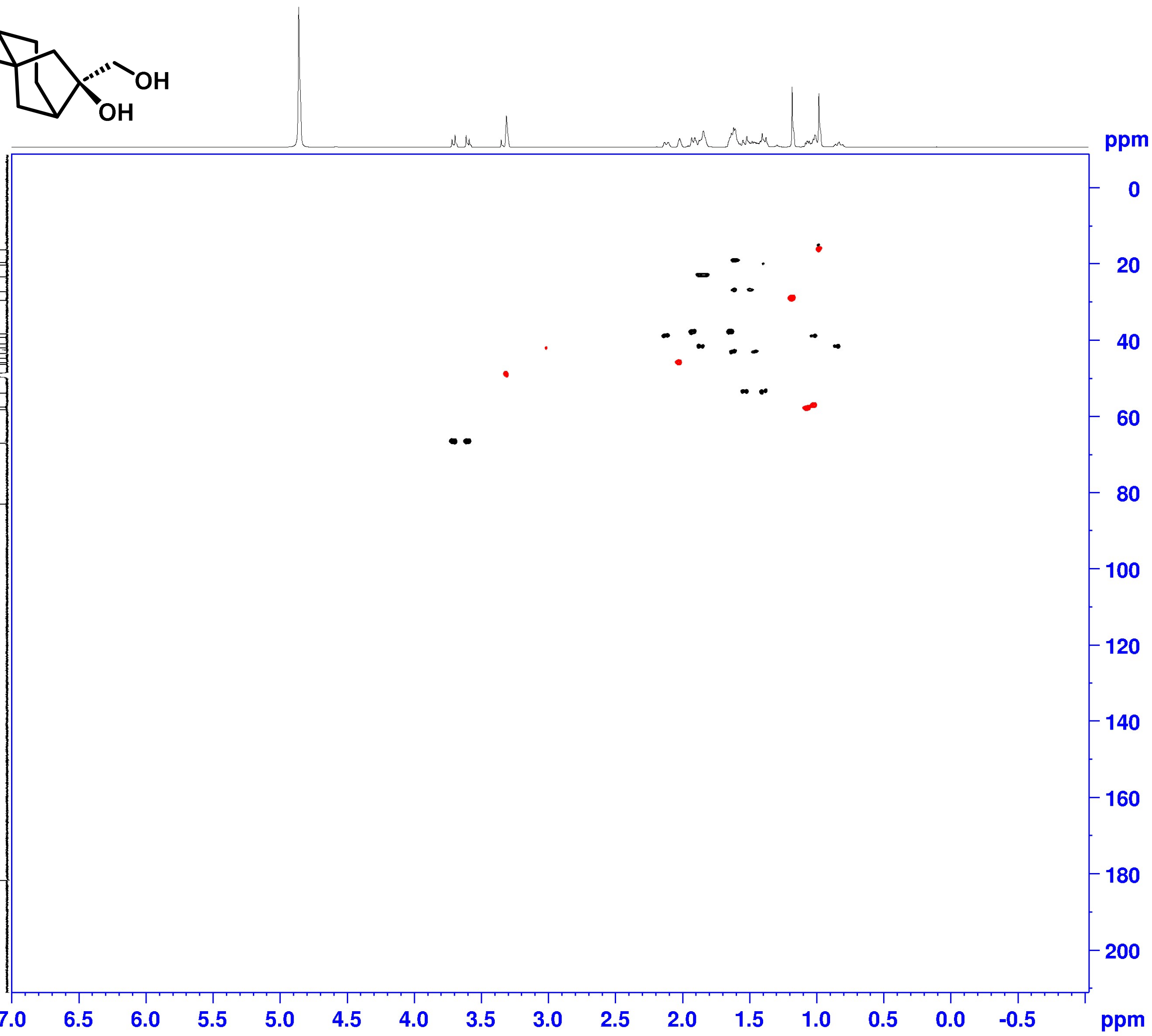


```
Current Data Parameters
NAME      diol do caurenoico
EXPNO                      5
PROCNO                     1

F2 - Acquisition Parameters
Date_           20250814
Time                5.50 h
INSTRUM            spect
PROBHD    Z113652_0120 (
PULPROG       hsqcedetgp
TD                  2048
SOLVENT            CD3OH
NS                     6
DS                    16
SWH             6996.269 Hz
FIDRES          6.832294 Hz
AQ             0.1463637 sec
RG                   203
DW                71.467 usec
DE                 10.00 usec
TE                 298.2 K
CNST2        145.0000000
D0            0.00000300 sec
D1            2.00000000 sec
D4            0.00172414 sec
D11           0.03000000 sec
D13           0.00000400 sec
D16           0.00020000 sec
D21           0.00344828 sec
IN0           0.00001810 sec
TDav                   1
ZGOPTNS
SFO1         499.8729992 MHz
NUC1                  1H
P1                 12.40 usec
P2                 24.80 usec
P28                    0 usec
PLW1         27.00000000 W
SFO2         125.7049802 MHz
NUC2                 13C
CPDPRG[2            garp
P3                 10.00 usec
P4                 20.00 usec
PCPD2              70.00 usec
PLW2         88.00000000 W
PLW12         1.79589999 W
GPNAM[1]      SMSQ10.100
GPZ1               80.00 %
GPNAM[2]      SMSQ10.100
GPZ2               20.10 %
P16              1000.00 usec

F1 - Acquisition parameters
TD                   256
SFO1             125.705 MHz
FIDRES        215.814911 Hz
SW               219.755 ppm
FnMODE     Echo-Antiecho

F2 - Processing parameters
SI                  4096
SF           499.8700146 MHz
WDW                QSINE
SSB                    2
LB                     0 Hz
GB                     0
PC                  1.40

F1 - Processing parameters
SI                  1024
MC2       echo-antiecho
SF           125.6922621 MHz
WDW                QSINE
SSB                    2
LB                     0 Hz
GB                     0
```

HMBC of compound 10:

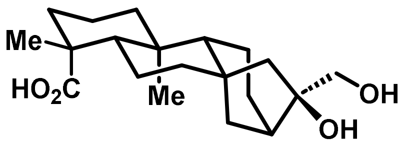

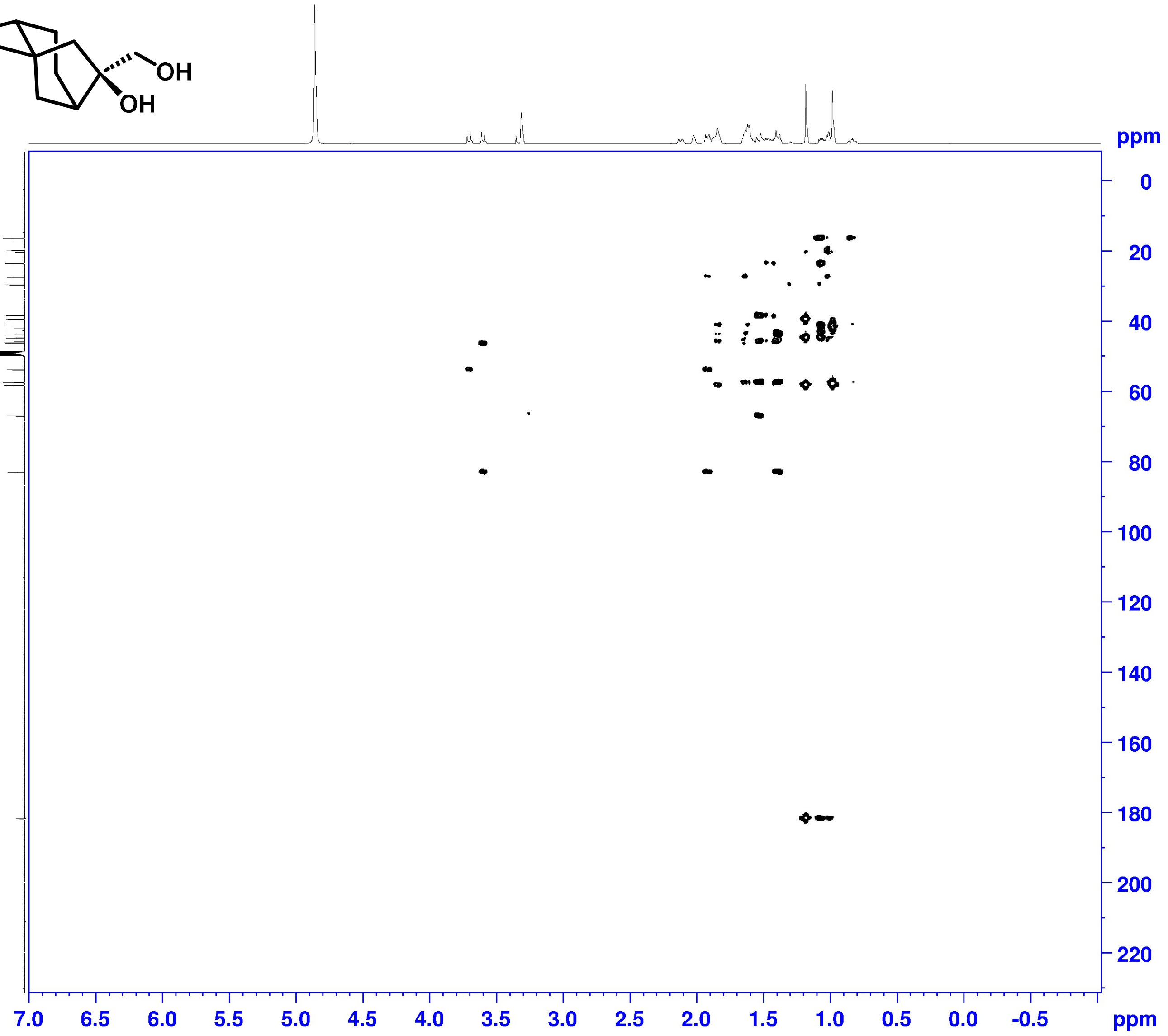


```
Current Data Parameters
NAME      diol do caurenoico
EXPNO                  6
PROCNO                 1

F2 - Acquisition Parameters
Date_           20250814
Time                6.47 h
INSTRUM            spect
PROBHD   Z113652_0120 (
PULPROG     hmbcgplpndqf
TD                  2048
SOLVENT            CD3OH
NS                     8
DS                    16
SWH             6996.269 Hz
FIDRES          6.832294 Hz
AQ             0.1463637 sec
RG                   203
DW                71.467 usec
DE                 10.00 usec
TE                 298.2 K
CNST2        145.0000000
CNST13         8.0000000
D0            0.00000300 sec
D1            1.42012799 sec
D2            0.00344828 sec
D6            0.06250000 sec
D16           0.00020000 sec
IN0           0.00001660 sec
TDav                   1
SFO1         499.8729992 MHz
NUC1                  1H
P1                 12.40 usec
P2                 24.80 usec
PLW1         27.00000000 W
SFO2         125.7062372 MHz
NUC2                 13C
P3                 10.00 usec
PLW2         88.00000000 W
GPNAM[1]      SMSQ10.100
GPZ1               50.00 %
GPNAM[2]      SMSQ10.100
GPZ2               30.00 %
GPNAM[3]      SMSQ10.100
GPZ3               40.10 %
P16              1000.00 usec

F1 - Acquisition parameters
TD                   256
SFO1            125.7062 MHz
FIDRES        235.316269 Hz
SW               239.610 ppm
FnMODE                QF

F2 - Processing parameters
SI                  4096
SF           499.8700155 MHz
WDW                 SINE
SSB                    0
LB                     0 Hz
GB                     0
PC                  1.40

F1 - Processing parameters
SI                  1024
MC2                   QF
SF           125.6922303 MHz
WDW                 SINE
SSB                    0
LB                     0 Hz
GB                     0
```

^{1}H NMR of compound 11:

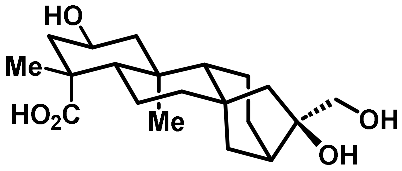

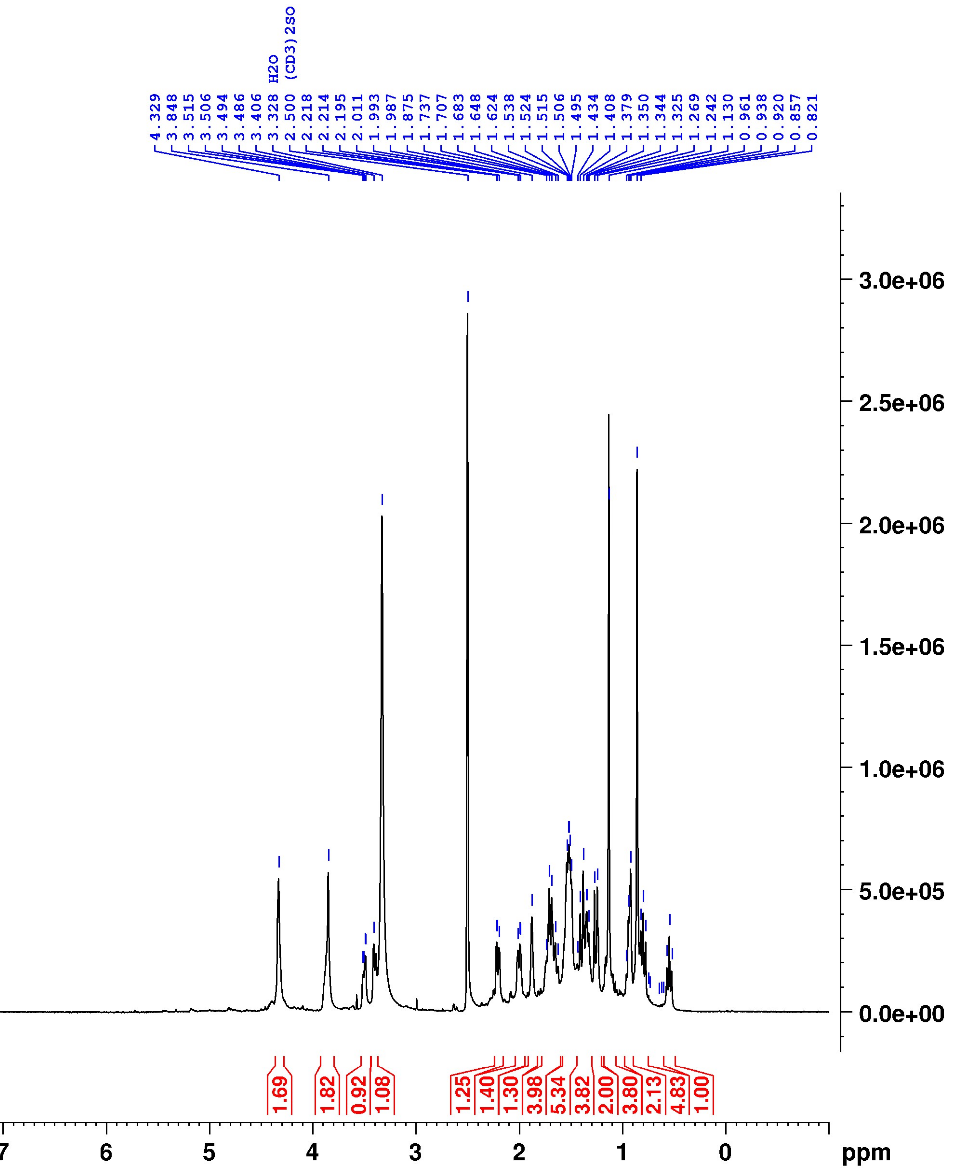


```
Current Data Parameters
NAME        Compound 11
EXPNO                 1
PROCNO                1

F2 - Acquisition Parameters
Date_          20250825
Time              18.31 h
INSTRUM           spect
PROBHD   Z113652_0120 (
PULPROG            zg30
TD                65536
SOLVENT            DMSO
NS                   16
DS                    0
SWH           10302.198 Hz
FIDRES         0.314398 Hz
AQ            3.1806805 sec
RG                  128
DW               48.533 usec
DE                10.00 usec
TE                298.2 K
D1           1.00000000 sec
TD0                   1
SFO1        499.8730869 MHz
NUC1                 1H
P1                12.40 usec
PLW1        27.00000000 W

F2 - Processing parameters
SI                65536
SF          499.8700041 MHz
WDW                  EM
SSB                   0
LB                 0.30 Hz
GB                    0
PC                 1.00
```

^{13}C NMR of compound 11:

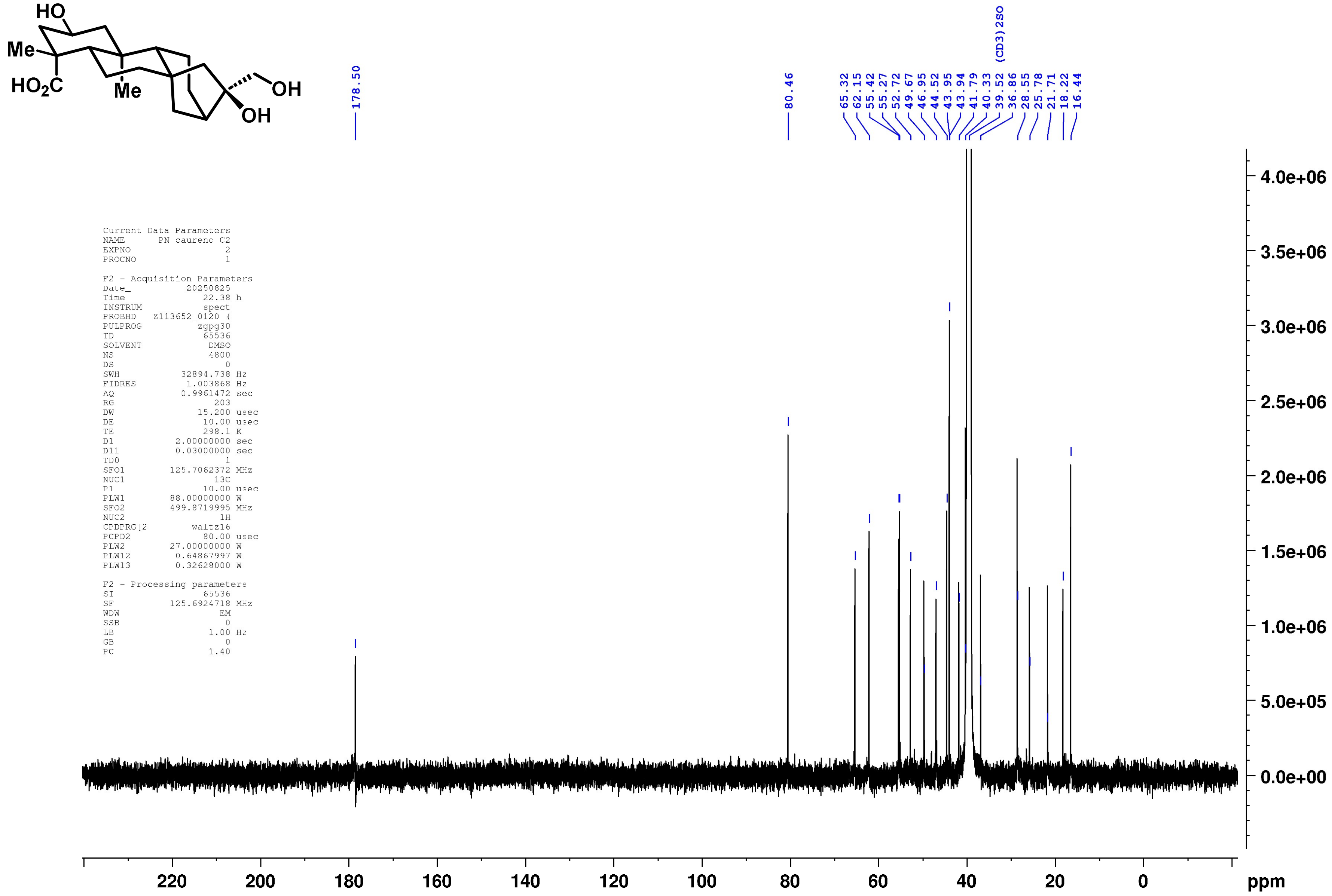

COSY of compound 11:

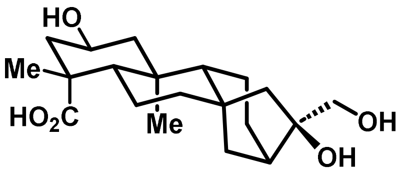

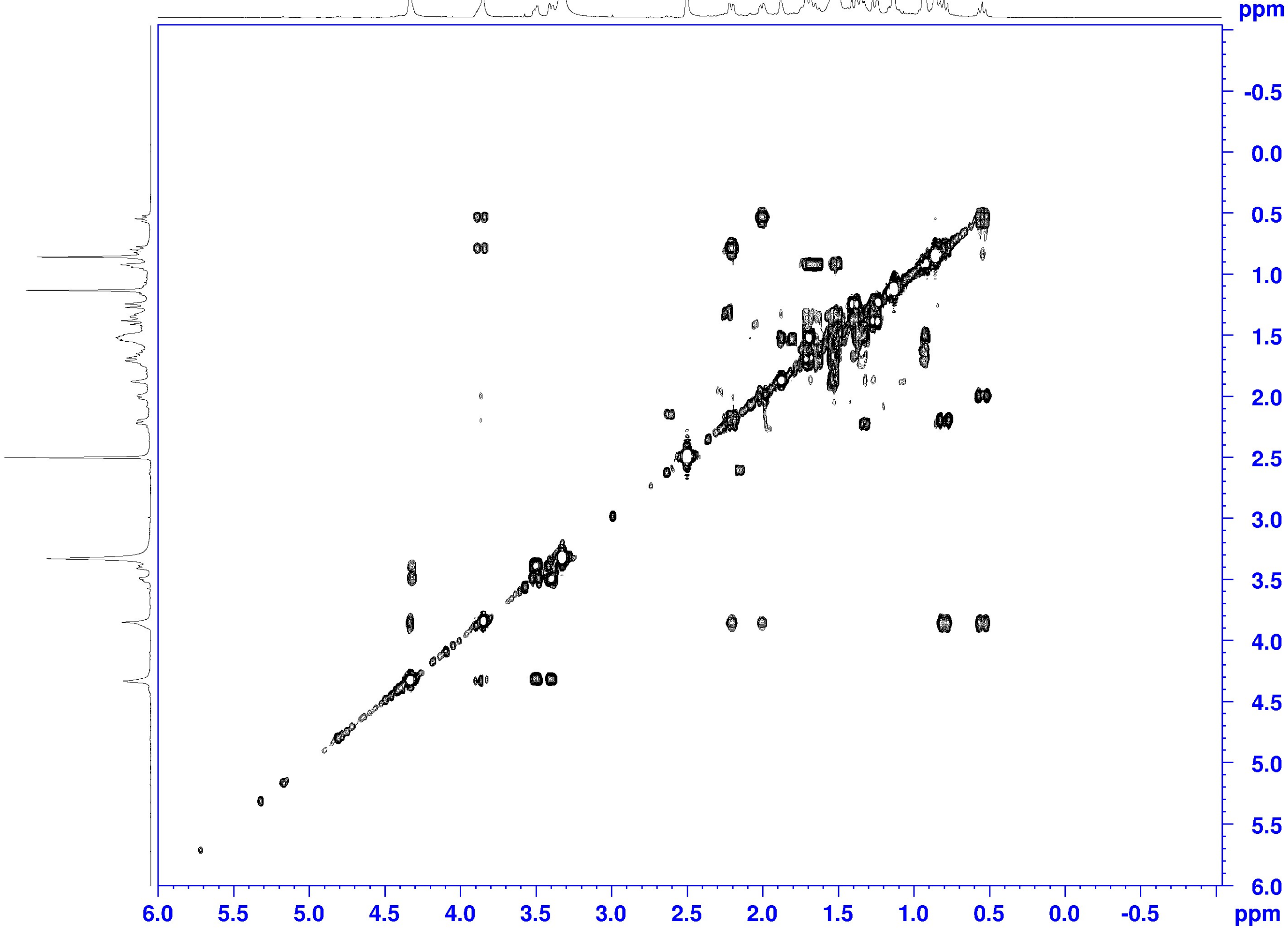


```
Current Data Parameters
NAME        PN caureno C2
EXPNO                   4
PROCNO                  1

F2 - Acquisition Parameters
Date_            20250828
Time                 5.19 h
INSTRUM             spect
PROBHD   Z113652_0120 (
PULPROG        cosygpppqf
TD                   2048
SOLVENT              DMSO
NS                      4
DS                     16
SWH              7352.941 Hz
FIDRES           7.180607 Hz
AQ              0.1392640 sec
RG                     64
DW                 68.000 usec
DE                  10.00 usec
TE                  298.2 K
D0             0.00000300 sec
D1             2.01843190 sec
D11            0.03000000 sec
D12            0.00002000 sec
D13            0.00000400 sec
D16            0.00020000 sec
IN0            0.00013600 sec
TDav                    1
SFO1          499.8731626 MHz
NUC1                   1H
P0                  12.40 usec
P1                  12.40 usec
P17               2500.00 usec
PLW1          27.00000000 W
PLW10          4.61280012 W
GPNAM[1]       SMSQ10.100
GPZ1                10.00 %
P16               1000.00 usec

F1 - Acquisition parameters
TD                    256
SFO1             499.8732 MHz
FIDRES          57.444855 Hz
SW                 14.710 ppm
FnMODE                 QF

F2 - Processing parameters
SI                   4096
SF            499.8700049 MHz
WDW                 QSINE
SSB                     0
LB                      0 Hz
GB                      0
PC                   1.40

F1 - Processing parameters
SI                   1024
MC2                    QF
SF            499.8700045 MHz
WDW                 QSINE
SSB                     0
LB                      0 Hz
GB                      0
```

NOESY of compound 11:

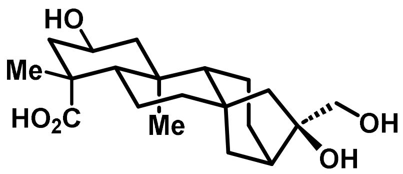

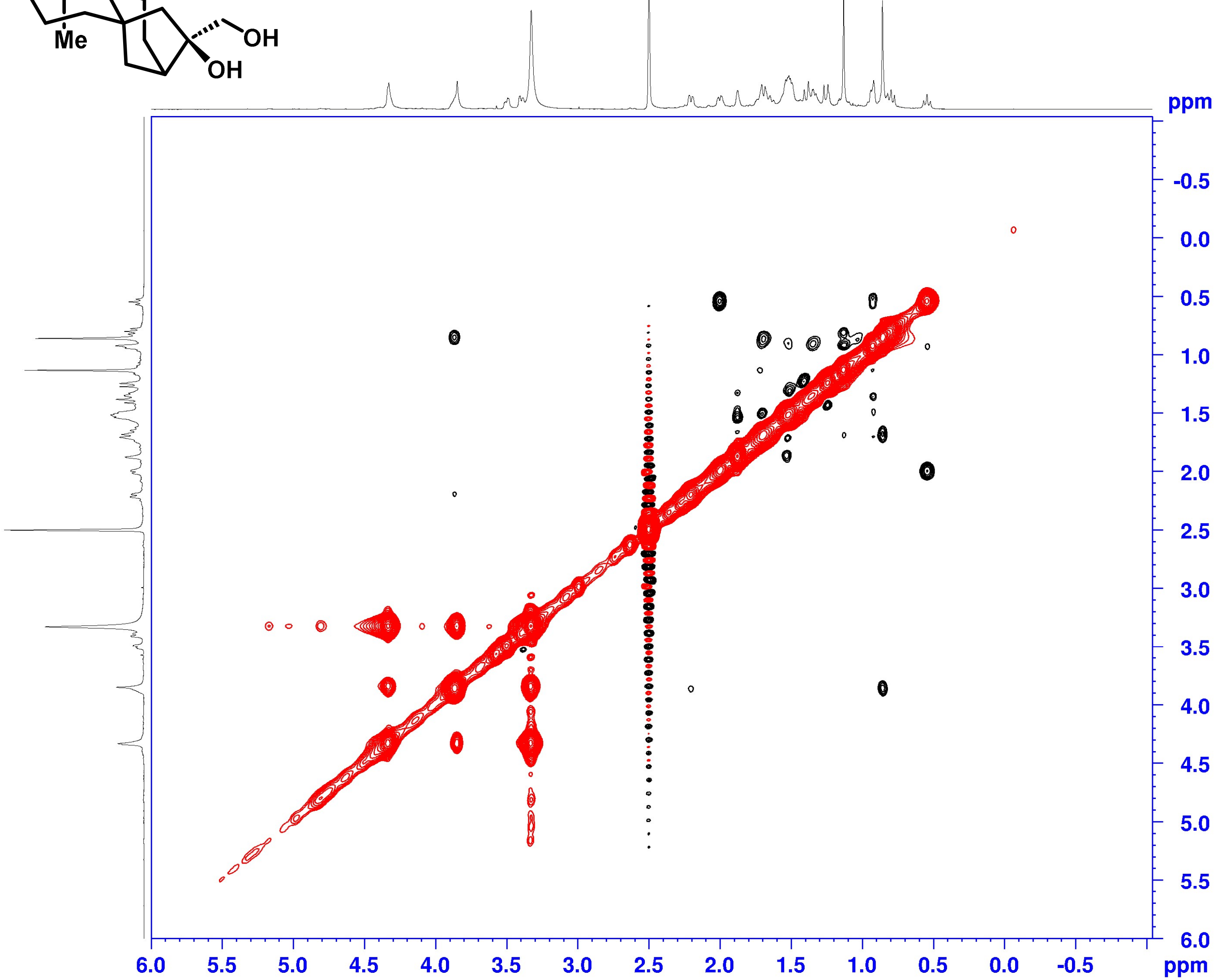


```
Current Data Parameters
NAME        PN caureno C2
EXPNO                   5
PROCNO                  1

F2 - Acquisition Parameters
Date_            20250828
Time                5.58 h
INSTRUM             spect
PROBHD    Z113652_0120 (
PULPROG          noesygpph
TD                   2048
SOLVENT              DMSO
NS                      6
DS                     16
SWH              7352.941 Hz
FIDRES           7.180607 Hz
AQ              0.1392640 sec
RG                     57
DW                 68.000 usec
DE                  10.00 usec
TE                  298.1 K
D0             0.00005221 sec
D1             2.01843190 sec
D8             0.80000001 sec
D16            0.00020000 sec
IN0            0.00013600 sec
TDav                    1
SFO1          499.8731626 MHz
NUC1                   1H
P1                  12.40 usec
P2                  24.80 usec
PLW1          27.00000000 W
GPNAM[1]       SMSQ10.100
GPZ1                40.00 %
P16               1000.00 usec

F1 - Acquisition parameters
TD                    256
SFO1             499.8732 MHz
FIDRES          57.444855 Hz
SW                 14.710 ppm
FnMODE               TPPI

F2 - Processing parameters
SI                   4096
SF            499.8700041 MHz
WDW                    GM
SSB                     0
LB                  -1.00 Hz
GB                  0.005
PC                   1.00

F1 - Processing parameters
SI                   1024
MC2                  TPPI
SF            499.8700046 MHz
WDW                    GM
SSB                     0
LB                  -1.00 Hz
GB                   0.01
```

HSQC of compound 11:

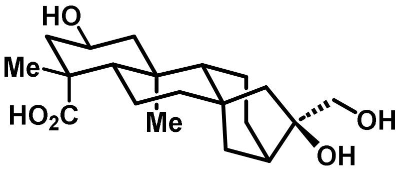

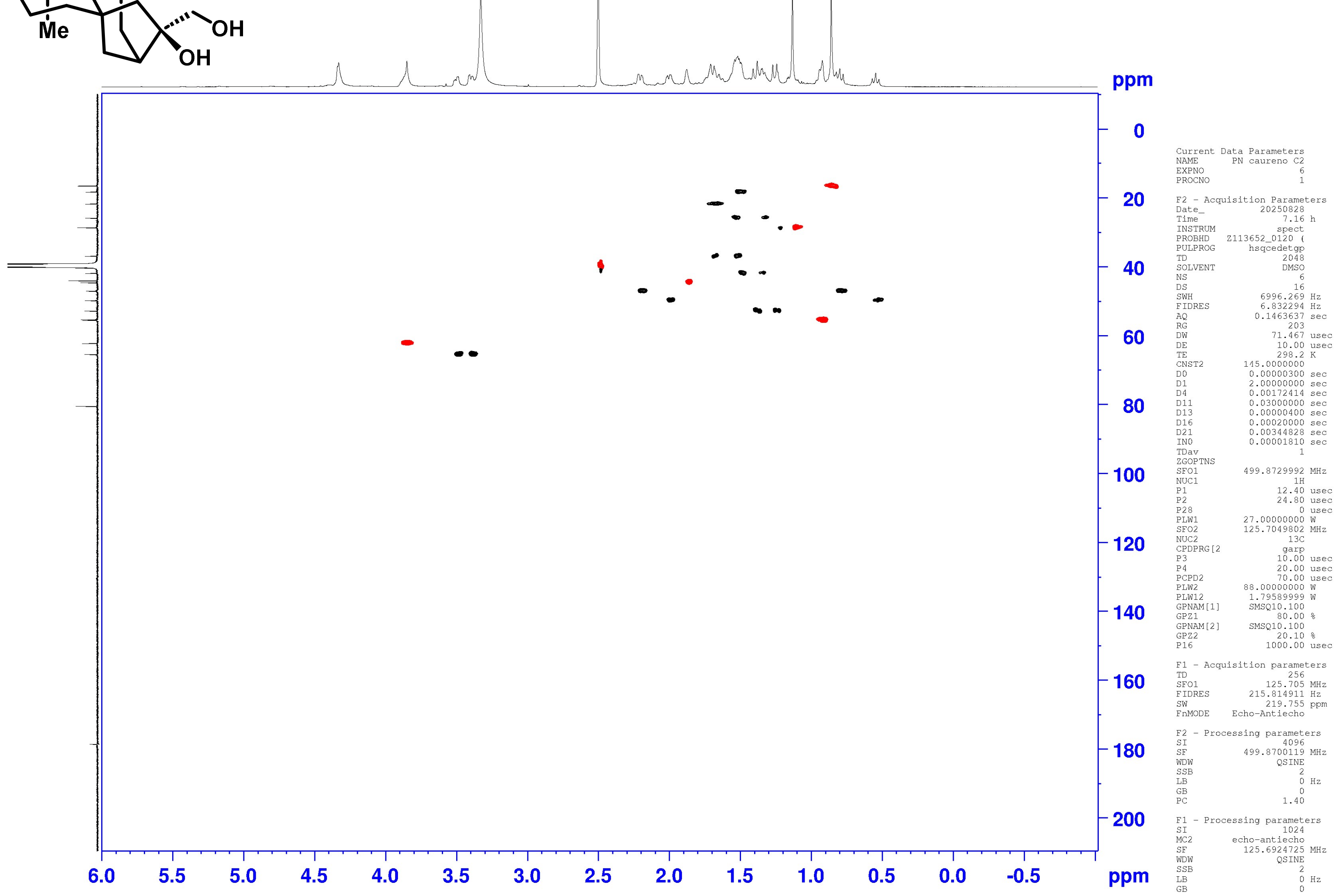

HMBC of compound 11:

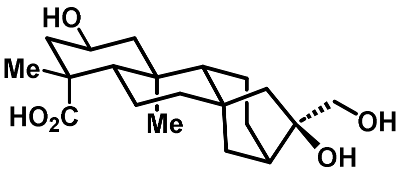

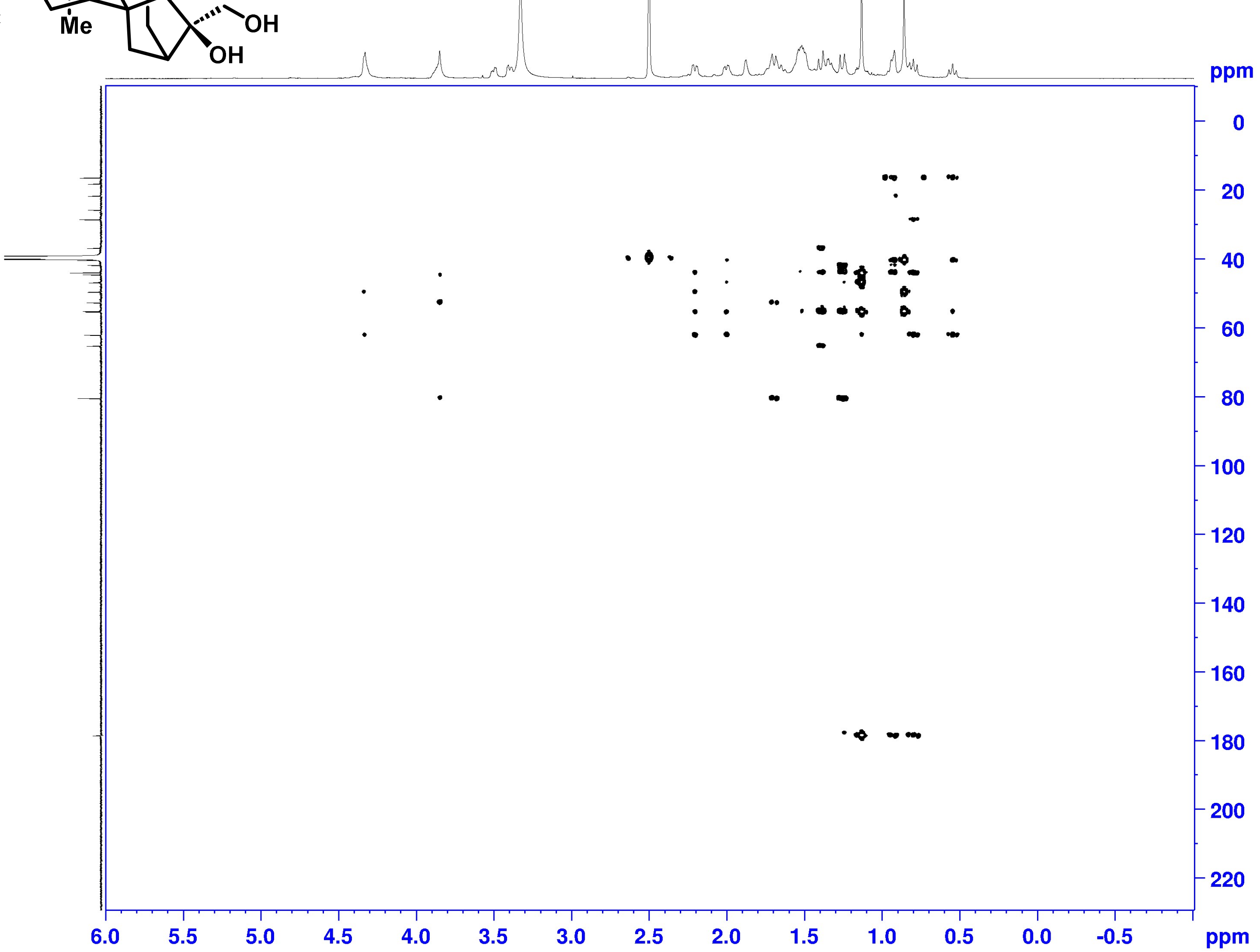


```
Current Data Parameters
NAME        PN caureno C2
EXPNO                   7
PROCNO                  1

F2 - Acquisition Parameters
Date_            20250828
Time                 8.13 h
INSTRUM             spect
PROBHD   Z113652_0120 (
PULPROG     hmbcgplpndqf
TD                   2048
SOLVENT              DMSO
NS                      8
DS                     16
SWH              6996.269 Hz
FIDRES           6.832294 Hz
AQ              0.1463637 sec
RG                    203
DW                 71.467 usec
DE                  10.00 usec
TE                  298.2 K
CNST2         145.0000000
CNST13          8.0000000
D0             0.00000300 sec
D1             1.42012799 sec
D2             0.00344828 sec
D6             0.06250000 sec
D16            0.00020000 sec
IN0            0.00001660 sec
TDav                    1
SFO1          499.8729992 MHz
NUC1                   1H
P1                  12.40 usec
P2                  24.80 usec
PLW1          27.00000000 W
SFO2          125.7062372 MHz
NUC2                  13C
P3                  10.00 usec
PLW2          88.00000000 W
GPNAM[1]       SMSQ10.100
GPZ1                50.00 %
GPNAM[2]       SMSQ10.100
GPZ2                30.00 %
GPNAM[3]       SMSQ10.100
GPZ3                40.10 %
P16               1000.00 usec

F1 - Acquisition parameters
TD                    256
SFO1             125.7062 MHz
FIDRES         235.316269 Hz
SW                239.610 ppm
FnMODE                 QF

F2 - Processing parameters
SI                   4096
SF            499.8700055 MHz
WDW                  SINE
SSB                     0
LB                      0 Hz
GB                      0
PC                   1.40

F1 - Processing parameters
SI                   1024
MC2                    QF
SF            125.6924712 MHz
WDW                  SINE
SSB                     0
LB                      0 Hz
GB                      0
```

^{1}H NMR of compound 12:

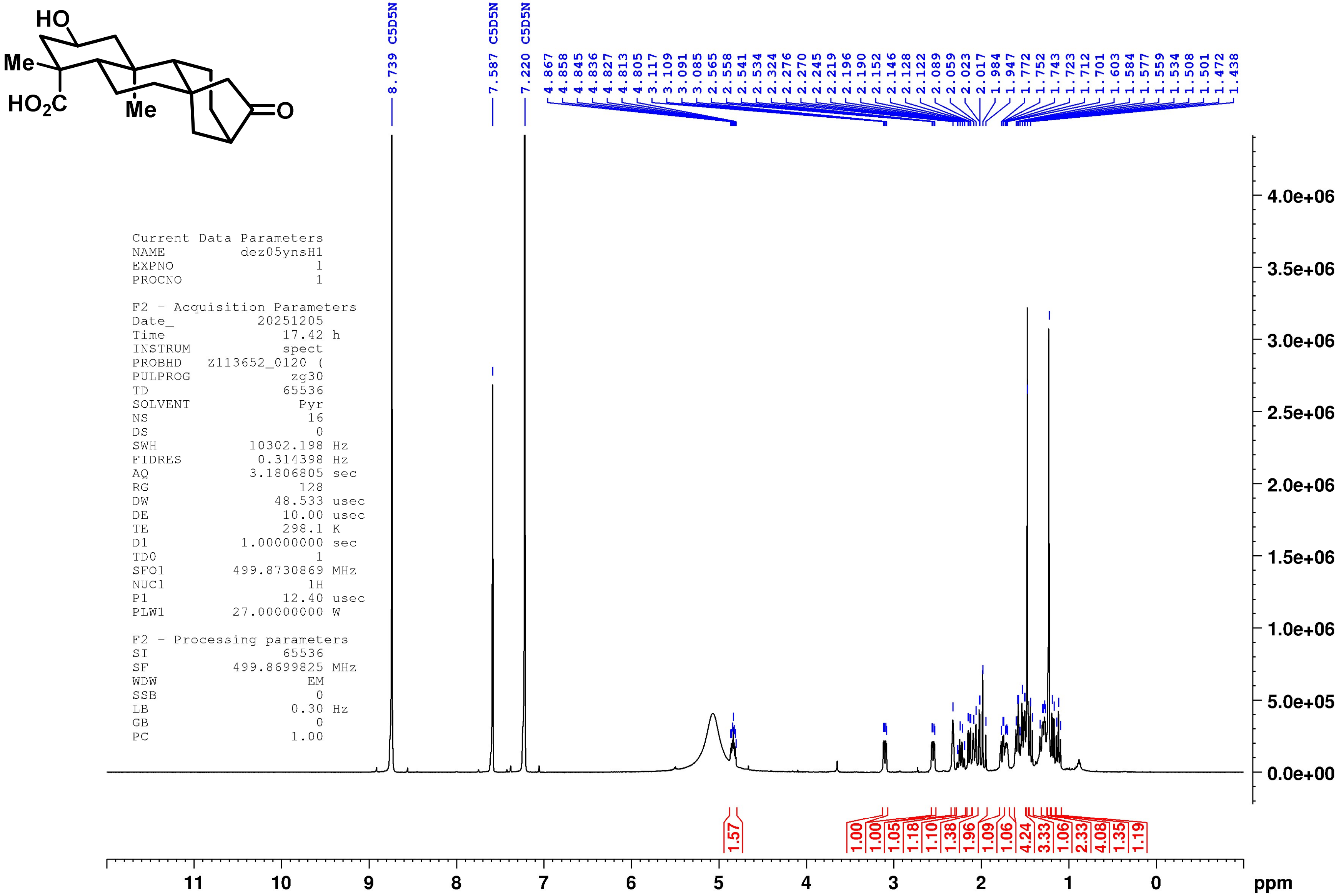

^{13}C NMR of compound 12:

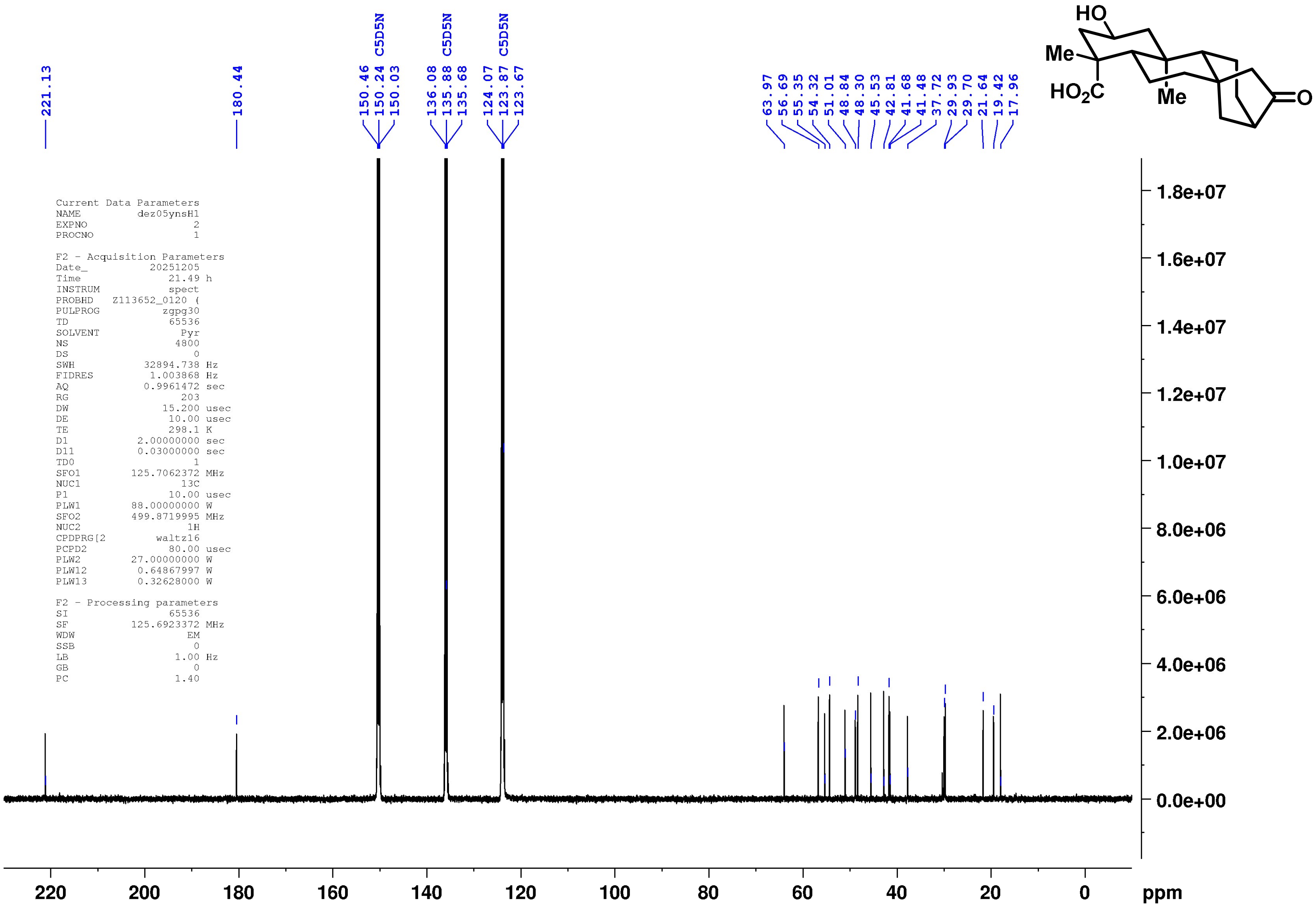

COSY of compound 12:

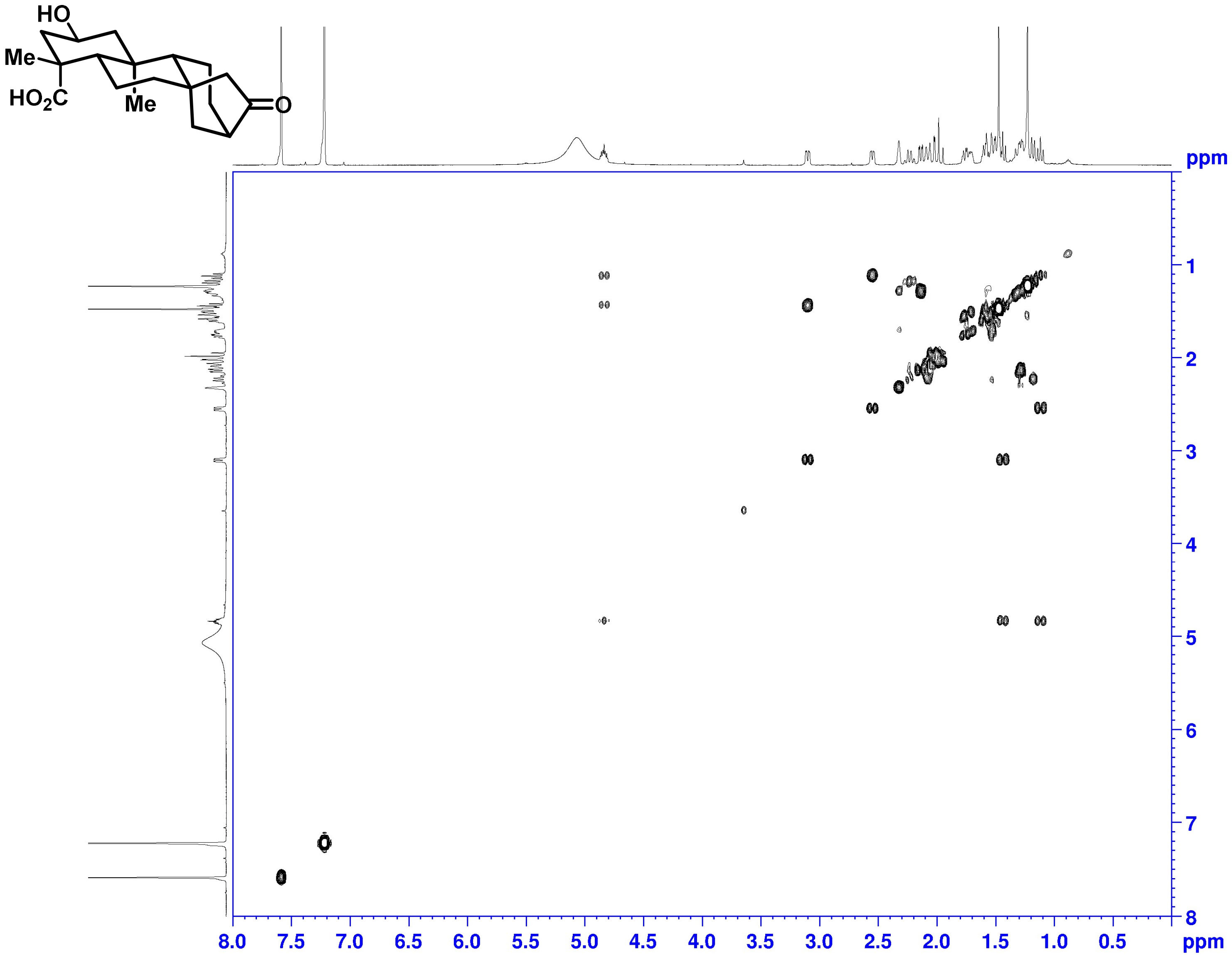


```
Current Data Parameters
NAME          dez05ynsH1
EXPNO                  3
PROCNO                 1

F2 - Acquisition Parameters
Date_           20251205
Time               21.50 h
INSTRUM            spect
PROBHD   Z113652_0120 (
PULPROG       cosygpppqf
TD                  2048
SOLVENT              Pyr
NS                     4
DS                    16
SWH            10302.198 Hz
FIDRES         10.060740 Hz
AQ             0.0993963 sec
RG                   203
DW                48.533 usec
DE                 10.00 usec
TE                 298.1 K
D0            0.00000300 sec
D1            2.00000000 sec
D11           0.03000000 sec
D12           0.00002000 sec
D13           0.00000400 sec
D16           0.00020000 sec
IN0           0.00009700 sec
TDav                   1
SFO1         499.8730867 MHz
NUC1                  1H
P0                 12.40 usec
P1                 12.40 usec
P17              2500.00 usec
PLW1         27.00000000 W
PLW10         4.61280012 W
GPNAM[1]      SMSQ10.100
GPZ1               10.00 %
P16              1000.00 usec

F1 - Acquisition parameters
TD                   256
SFO1            499.8731 MHz
FIDRES         80.541237 Hz
SW                20.624 ppm
FnMODE                QF

F2 - Processing parameters
SI                  4096
SF           499.8699834 MHz
WDW                QSINE
SSB                    0
LB                     0 Hz
GB                     0
PC                  1.40

F1 - Processing parameters
SI                  1024
MC2                   QF
SF           499.8699831 MHz
WDW                QSINE
SSB                    0
LB                     0 Hz
GB                     0
```

NOESY of compound 12:

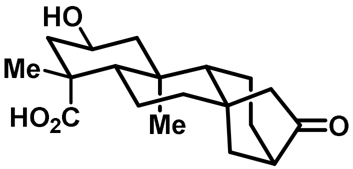

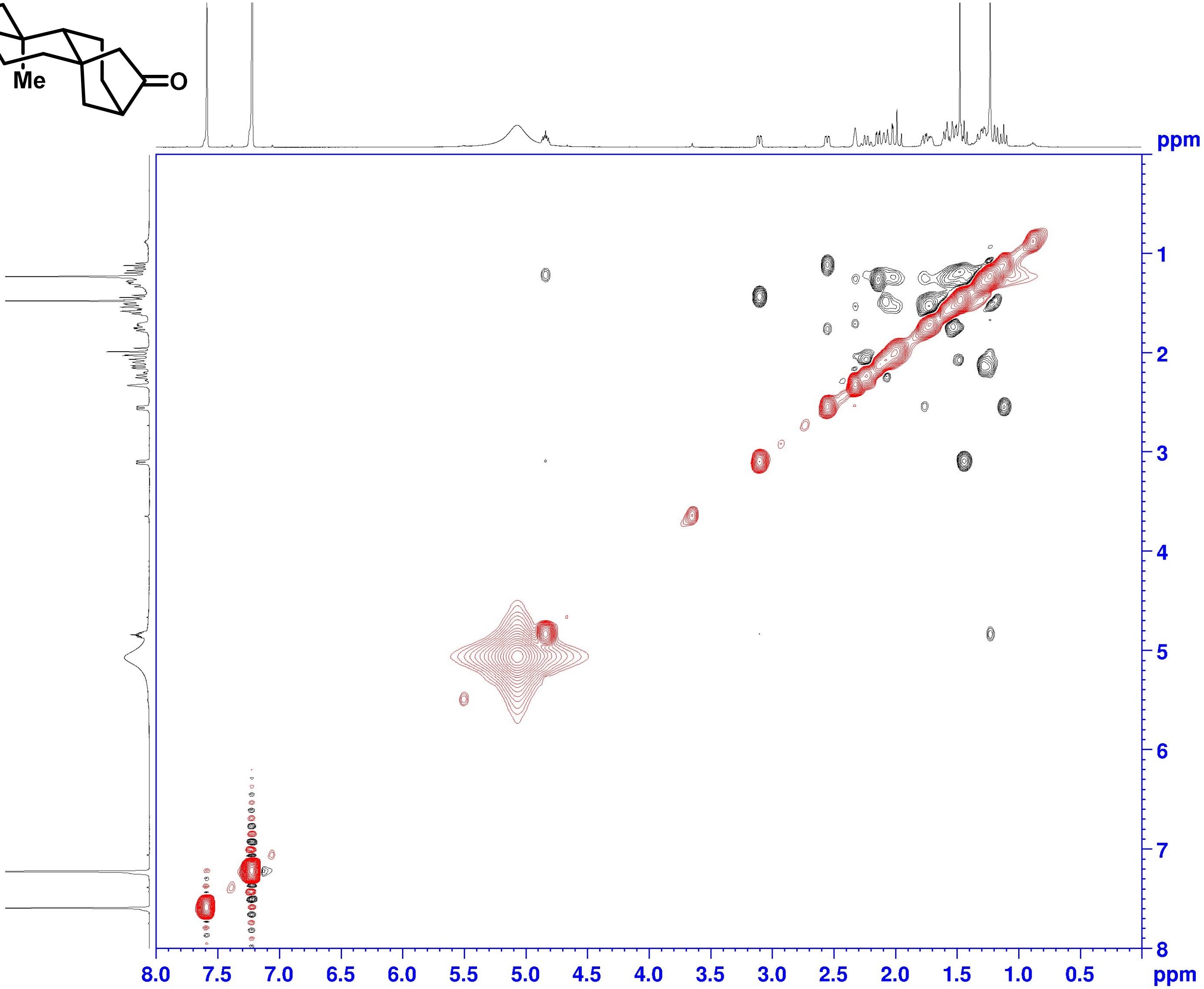


```
Current Data Parameters
NAME            dez05ynsH1
EXPNO                    4
PROCNO                   1

F2 - Acquisition Parameters
Date_             20251205
Time                 22.29 h
INSTRUM              spect
PROBHD    Z113652_0120 (
PULPROG          noesygpph
TD                    2048
SOLVENT                Pyr
NS                       6
DS                      16
SWH              10302.198 Hz
FIDRES           10.060740 Hz
AQ               0.0993963 sec
RG                     203
DW                  48.533 usec
DE                   10.00 usec
TE                   298.2 K
D0              0.00003271 sec
D1              2.01433611 sec
D8              0.80000001 sec
D16             0.00020000 sec
IN0             0.00009700 sec
TDav                     1
SFO1           499.8730867 MHz
NUC1                    1H
P1                   12.40 usec
P2                   24.80 usec
PLW1           27.00000000 W
GPNAM[1]        SMSQ10.100
GPZ1                 40.00 %
P16                1000.00 usec

F1 - Acquisition parameters
TD                     256
SFO1              499.8731 MHz
FIDRES           80.541237 Hz
SW                  20.624 ppm
FnMODE                TPPI

F2 - Processing parameters
SI                    4096
SF             499.8699838 MHz
WDW                     GM
SSB                      0
LB                   -1.00 Hz
GB                   0.005
PC                    1.00

F1 - Processing parameters
SI                    1024
MC2                   TPPI
SF             499.8699851 MHz
WDW                     GM
SSB                      0
LB                   -1.00 Hz
GB                    0.01
```

HSQC of compound 12:

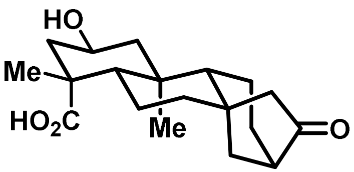

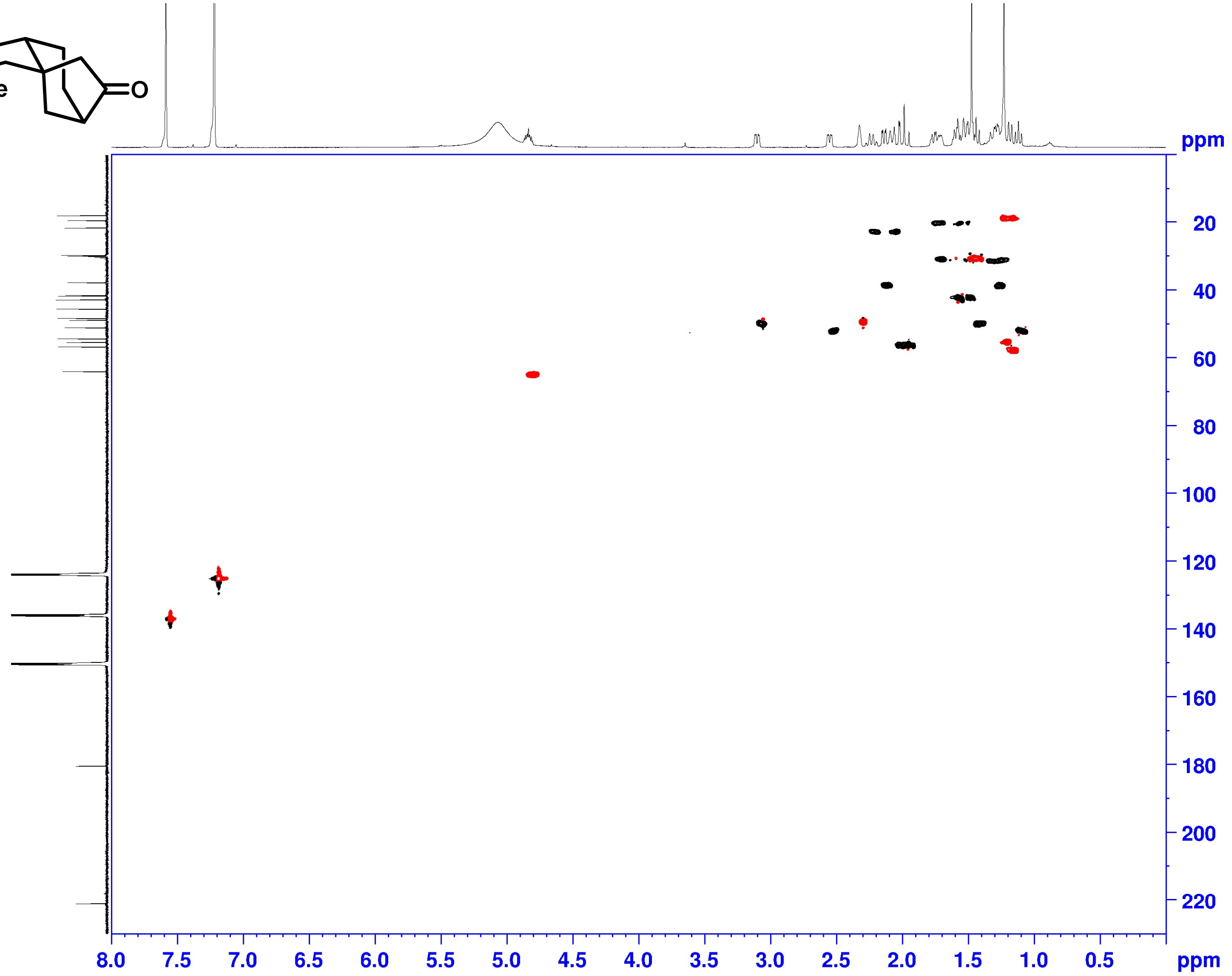


```
Current Data Parameters
NAME          dez05ynsH1
EXPNO                  5
PROCNO                 1

F2 - Acquisition Parameters
Date_           20251205
Time               23.45 h
INSTRUM            spect
PROBHD   Z113652_0120 (
PULPROG       hsqcedetgp
TD                  2048
SOLVENT              Pyr
NS                     6
DS                    16
SWH            10302.198 Hz
FIDRES         10.060740 Hz
AQ             0.0993963 sec
RG                   203
DW                48.533 usec
DE                 10.00 usec
TE                 298.2 K
CNST2        145.0000000
D0            0.00000300 sec
D1            2.00000000 sec
D4            0.00172414 sec
D11           0.03000000 sec
D13           0.00000400 sec
D16           0.00020000 sec
D21           0.00344828 sec
IN0           0.00001520 sec
TDav                   1
ZGOPTNS
SFO1         499.8730867 MHz
NUC1                  1H
P1                 12.40 usec
P2                 24.80 usec
P28                    0 usec
PLW1         27.00000000 W
SFO2         125.7062372 MHz
NUC2                 13C
CPDPRG[2            garp
P3                 10.00 usec
P4                 20.00 usec
PCPD2              70.00 usec
PLW2         88.00000000 W
PLW12         1.79589999 W
GPNAM[1]      SMSQ10.100
GPZ1               80.00 %
GPNAM[2]      SMSQ10.100
GPZ2               20.10 %
P16              1000.00 usec

F1 - Acquisition parameters
TD                   256
SFO1            125.7062 MHz
FIDRES        256.990143 Hz
SW               261.679 ppm
FnMODE     Echo-Antiecho

F2 - Processing parameters
SI                  4096
SF           499.8699972 MHz
WDW                QSINE
SSB                    2
LB                     0 Hz
GB                     0
PC                  1.40

F1 - Processing parameters
SI                  1024
MC2        echo-antiecho
SF           125.6921974 MHz
WDW                QSINE
SSB                    2
LB                     0 Hz
GB                     0
```

HMBC of compound 12:

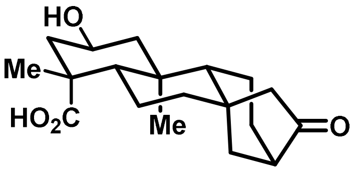

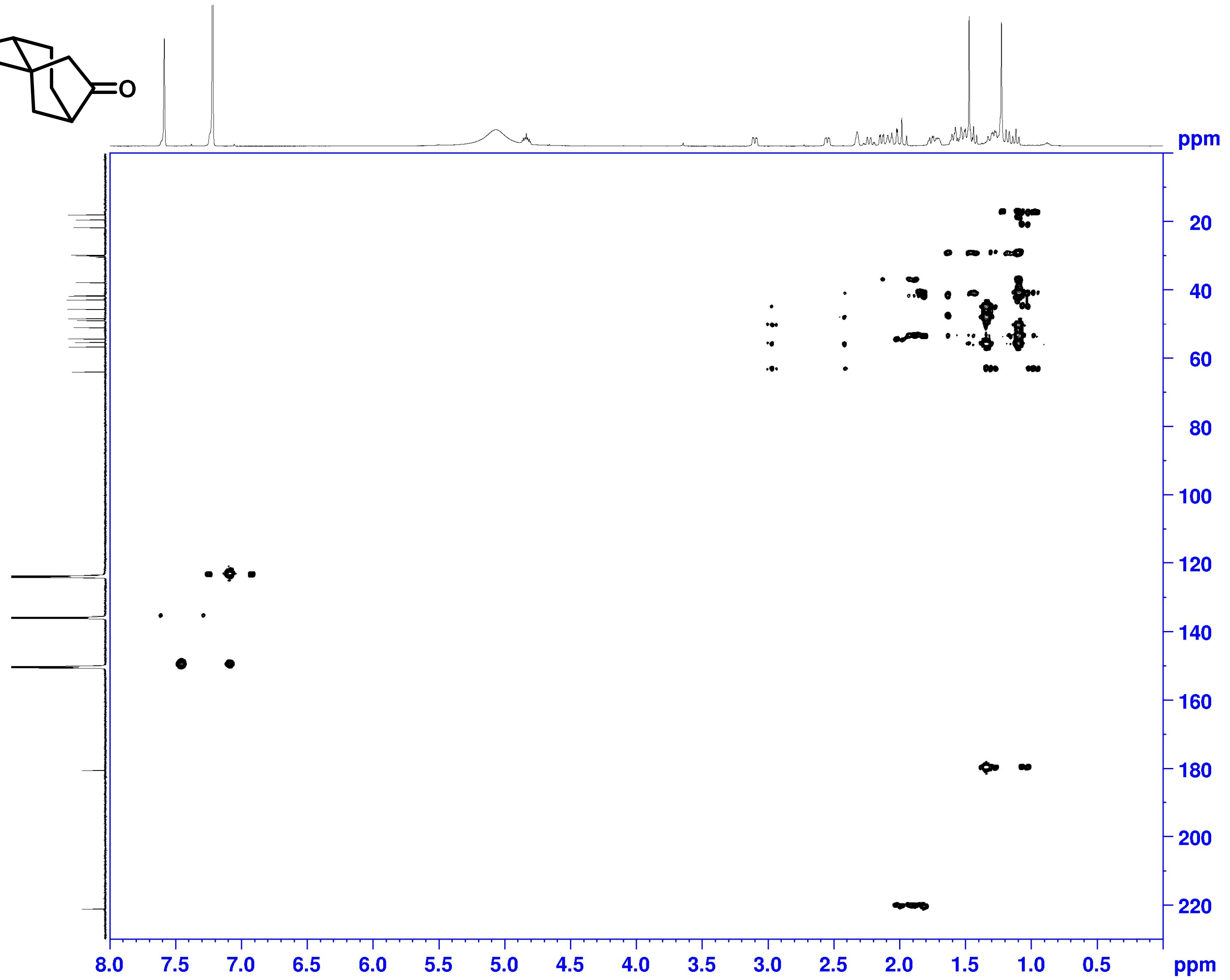


```
Current Data Parameters
NAME          dez05ynsH1
EXPNO                  6
PROCNO                 1

F2 - Acquisition Parameters
Date_           20251206
Time              0.41 h
INSTRUM            spect
PROBHD   Z113652_0120 (
PULPROG    hmbcgplpndqf
TD                  2048
SOLVENT              Pyr
NS                     8
DS                    16
SWH          10302.198 Hz
FIDRES       10.060740 Hz
AQ           0.0993963 sec
RG                   203
DW              48.533 usec
DE               10.00 usec
TE               298.1 K
CNST2      145.0000000
CNST13       8.0000000
D0          0.00000300 sec
D1          1.42012799 sec
D2          0.00344828 sec
D6          0.06250000 sec
D16         0.00020000 sec
IN0         0.00001520 sec
TDav                 1
SFO1       499.8730867 MHz
NUC1                1H
P1               12.40 usec
P2               24.80 usec
PLW1       27.00000000 W
SFO2       125.7062372 MHz
NUC2               13C
P3               10.00 usec
PLW2       88.00000000 W
GPNAM[1]    SMSQ10.100
GPZ1             50.00 %
GPNAM[2]    SMSQ10.100
GPZ2             30.00 %
GPNAM[3]    SMSQ10.100
GPZ3             40.10 %
P16            1000.00 usec

F1 - Acquisition parameters
TD                   256
SFO1          125.7062 MHz
FIDRES      256.990143 Hz
SW             261.679 ppm
FnMODE              QF

F2 - Processing parameters
SI                  4096
SF         499.8700486 MHz
WDW                SINE
SSB                    0
LB                     0 Hz
GB                     0
PC                  1.40

F1 - Processing parameters
SI                  1024
MC2                   QF
SF         125.6924110 MHz
WDW                SINE
SSB                    0
LB                     0 Hz
GB                     0
```

^{1}H NMR of compound 13:

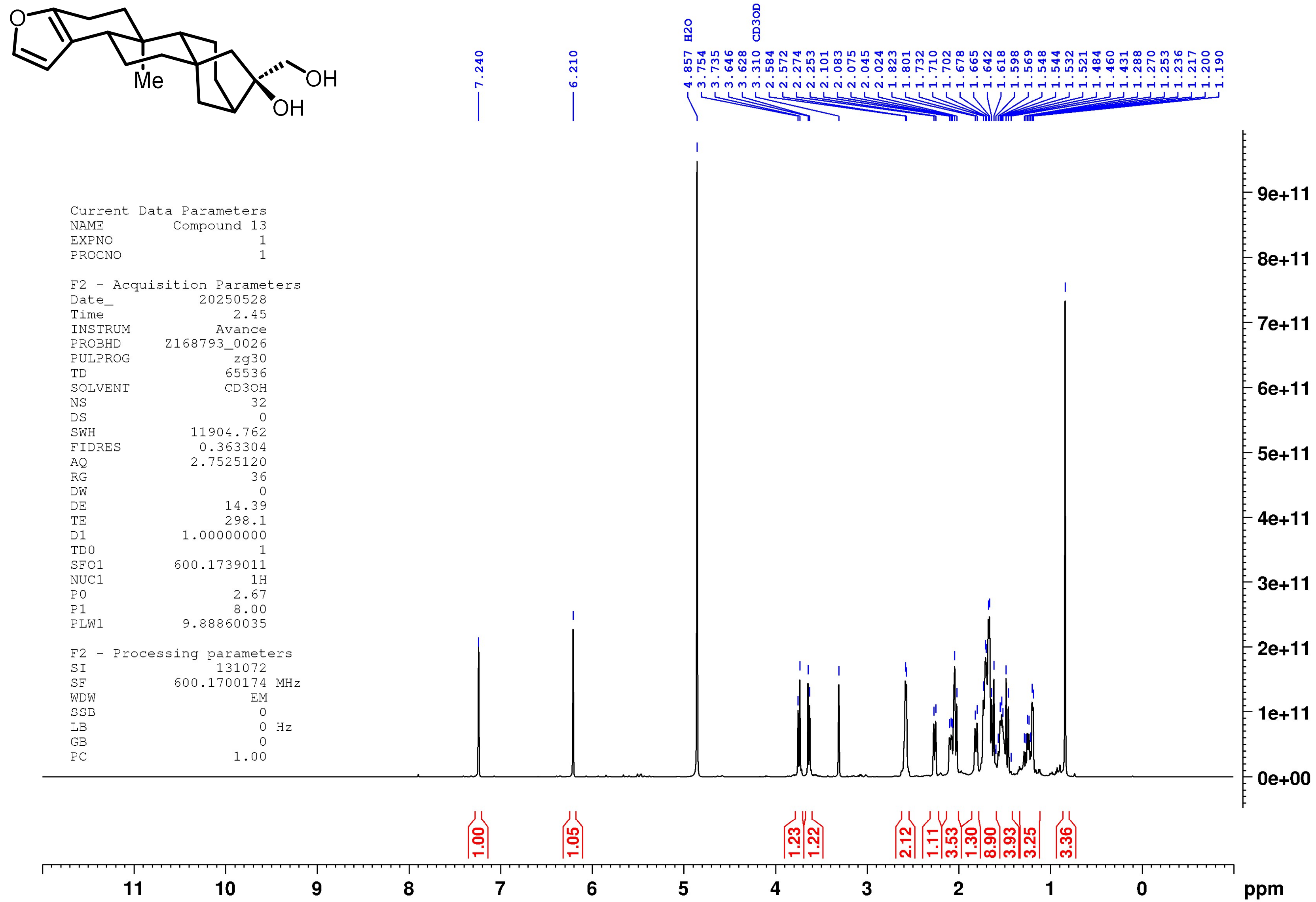

^{13}C NMR of compound 13:

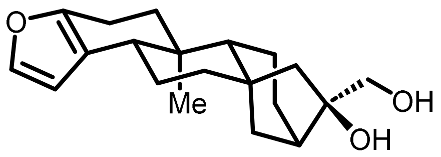

Current Data Parameters
NAME Compound 13
EXPNO 2
PROCNO 1

F2 - Acquisition Parameters
Date_ 20250528
Time 4.45
INSTRUM Avance
PROBHD Z168793_0026
PULPROG zgpg30
TD 65536
SOLVENT CD3OH
NS 2400
DS 4
SWH 35714.286
FIDRES 1.089913
AQ 0.9175040
RG 101
DW 0
DE 18.00
TE 298.1
D1 2.00000000
D11 0.03000000
TD0 1
SFO1 150.9294669
NUC1 13C
P0 4.00
P1 12.00
PLW1 172.83999634
SFO2 600.1724007
NUC2 1H
CPDPRG2 A000
PCPD2 80.00
PLW2 9.88860035
PLW12 0.09685679
PLW13 0.04854462

F2 - Processing parameters
SI 65536
SF 150.9126584 MHz
WDW EM
SSB 0
LB 2.00 Hz
GB 0
PC 1.40

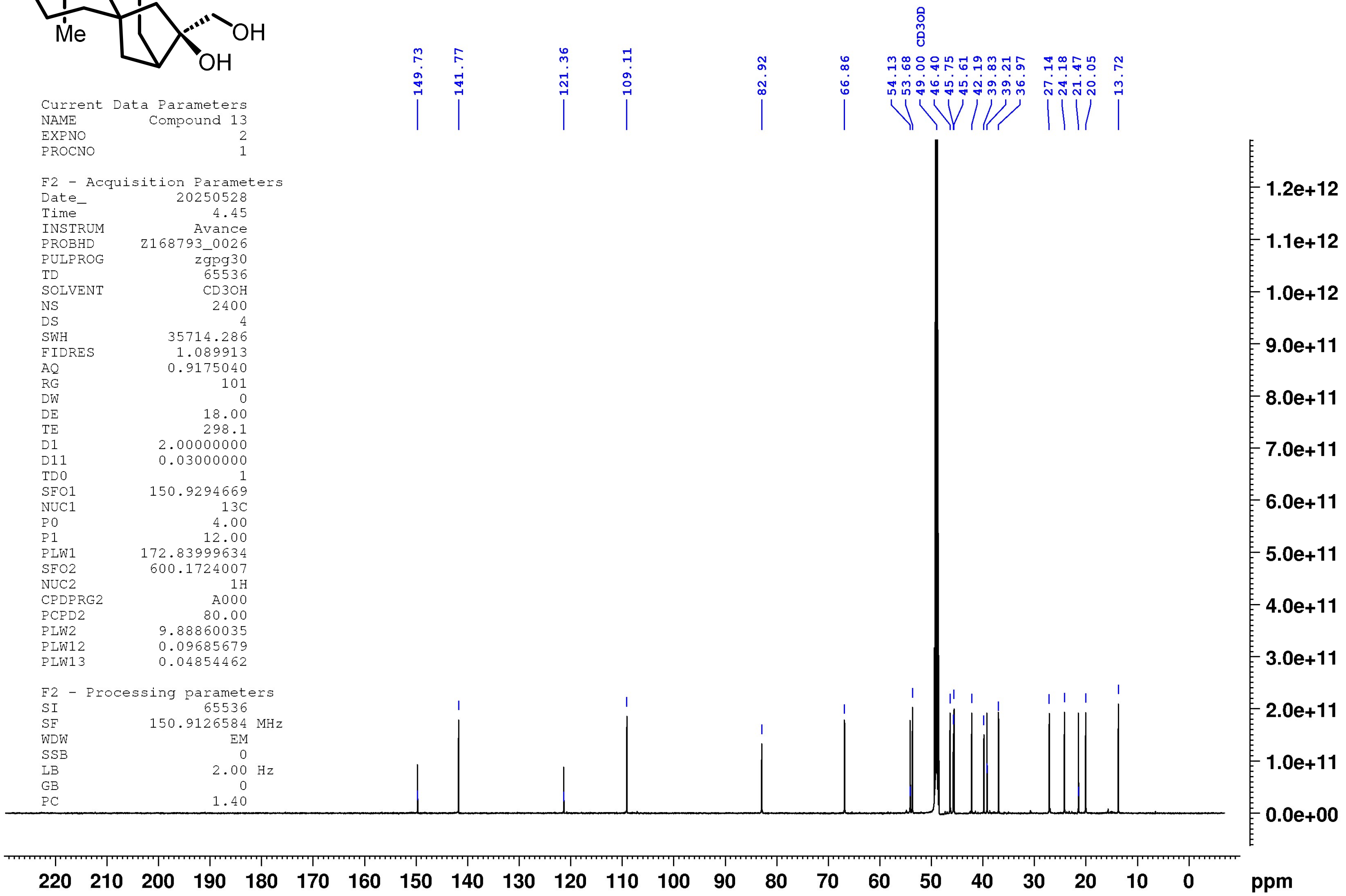

^{1}H NMR of compound 14:

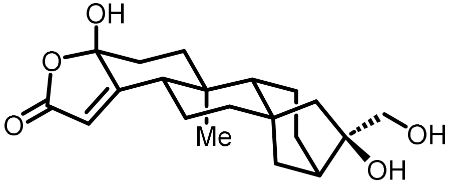

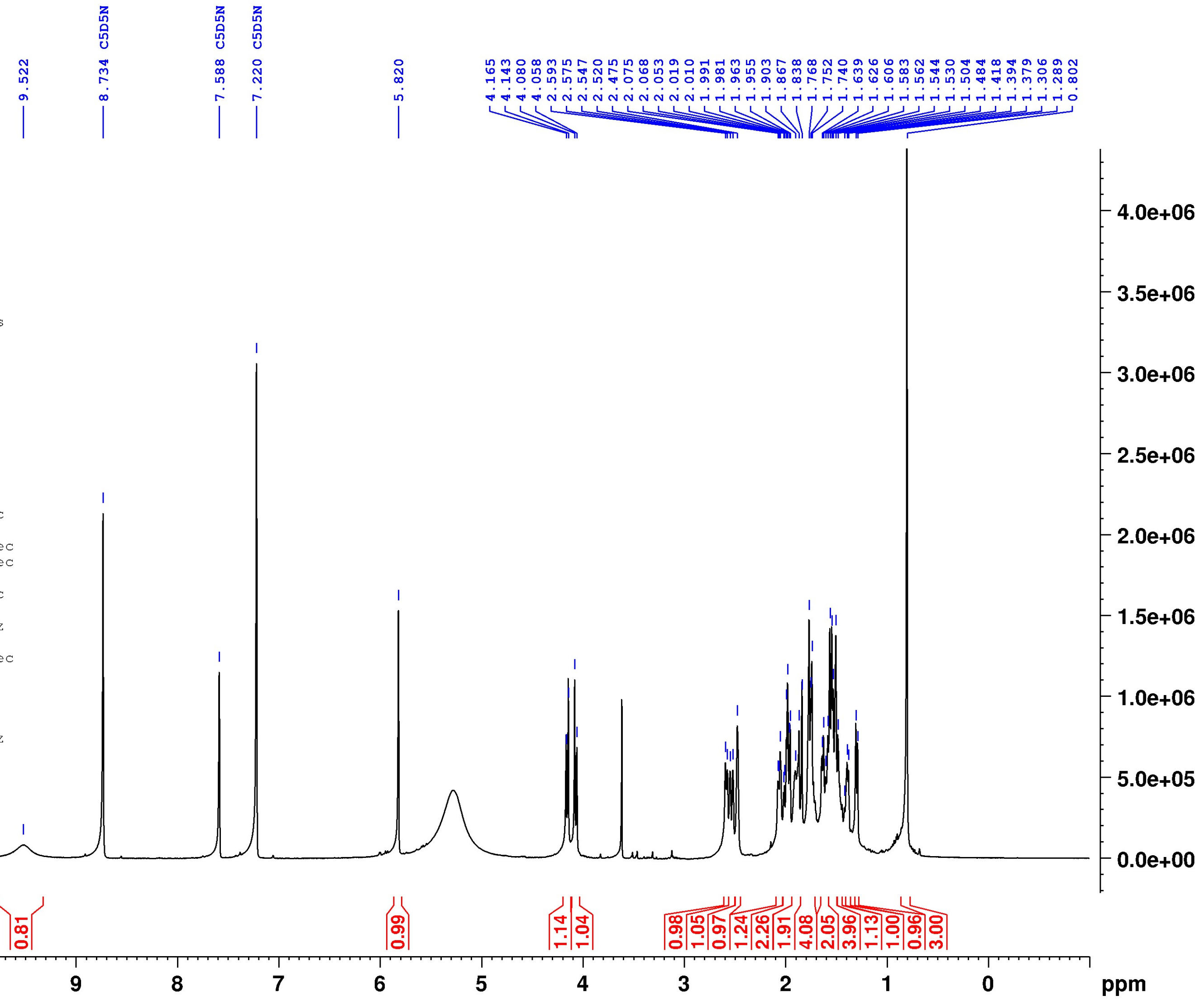


```
Current Data Parameters
NAME          jan12ynsH1
EXPNO                  1
PROCNO                 1

F2 - Acquisition Parameters
Date_           20260115
Time              1.16 h
INSTRUM            spect
PROBHD   Z113652_0120 (
PULPROG             zg30
TD                 65536
SOLVENT              Pyr
NS                    16
DS                     0
SWH            10302.198 Hz
FIDRES          0.314398 Hz
AQ             3.1806805 sec
RG                  90.5
DW                48.533 usec
DE                 10.00 usec
TE                 298.2 K
D1            1.00000000 sec
TD0                    1
SFO1         499.8730869 MHz
NUC1                  1H
P1                 12.40 usec
PLW1         27.00000000 W

F2 - Processing parameters
SI                 65536
SF           499.8699851 MHz
WDW                   EM
SSB                    0
LB                  0.30 Hz
GB                     0
PC                  1.00
```

^{13}C NMR of compound 14:

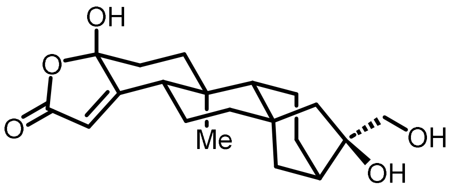

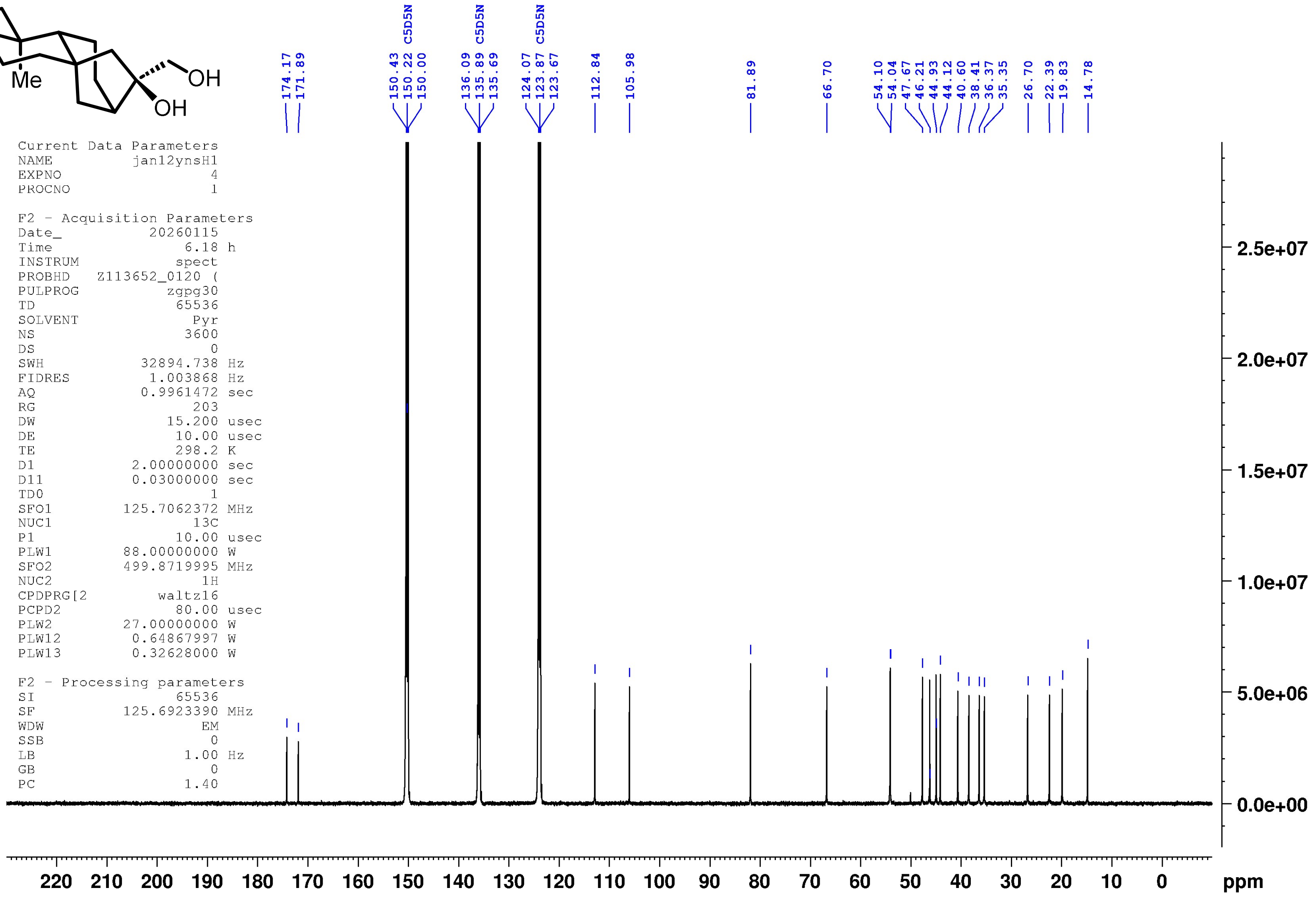


```
Current Data Parameters
NAME          jan12ynsH1
EXPNO                  4
PROCNO                 1

F2 - Acquisition Parameters
Date_           20260115
Time                6.18 h
INSTRUM            spect
PROBHD   Z113652_0120 (
PULPROG          zgpg30
TD                65536
SOLVENT              Pyr
NS                 3600
DS                    0
SWH           32894.738 Hz
FIDRES         1.003868 Hz
AQ            0.9961472 sec
RG                  203
DW               15.200 usec
DE                10.00 usec
TE                298.2 K
D1           2.00000000 sec
D11          0.03000000 sec
TD0                   1
SFO1        125.7062372 MHz
NUC1                13C
P1                10.00 usec
PLW1        88.00000000 W
SFO2        499.8719995 MHz
NUC2                 1H
CPDPRG[2        waltz16
PCPD2             80.00 usec
PLW2        27.00000000 W
PLW12        0.64867997 W
PLW13        0.32628000 W

F2 - Processing parameters
SI                65536
SF          125.6923390 MHz
WDW                  EM
SSB                   0
LB                 1.00 Hz
GB                    0
PC                 1.40
```

COSY of compound 14:

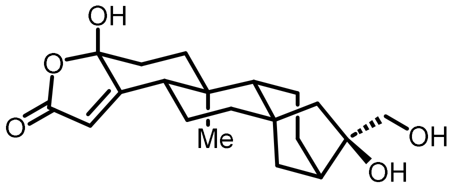

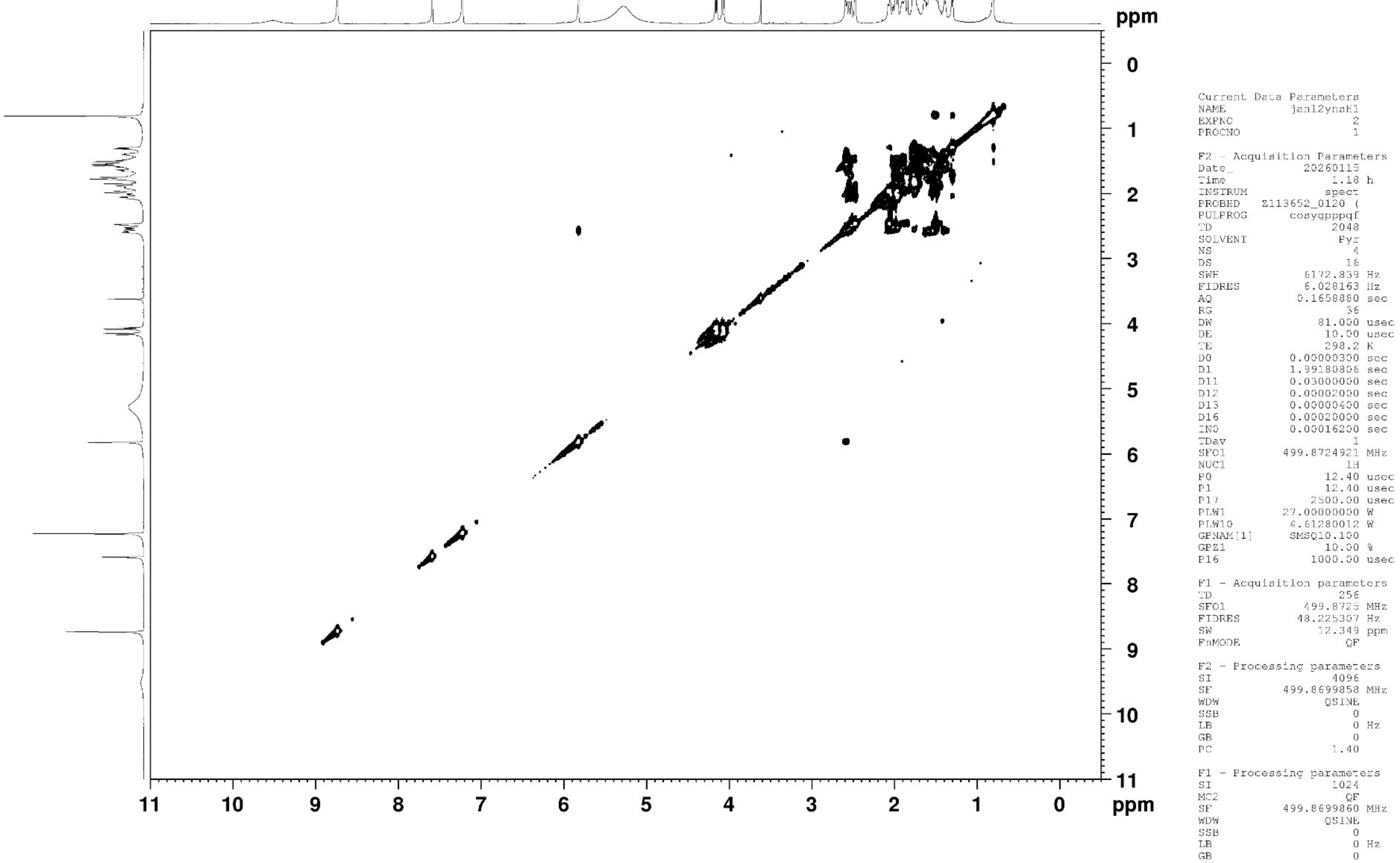

NOESY of compound 14:

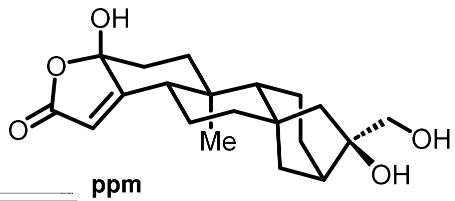

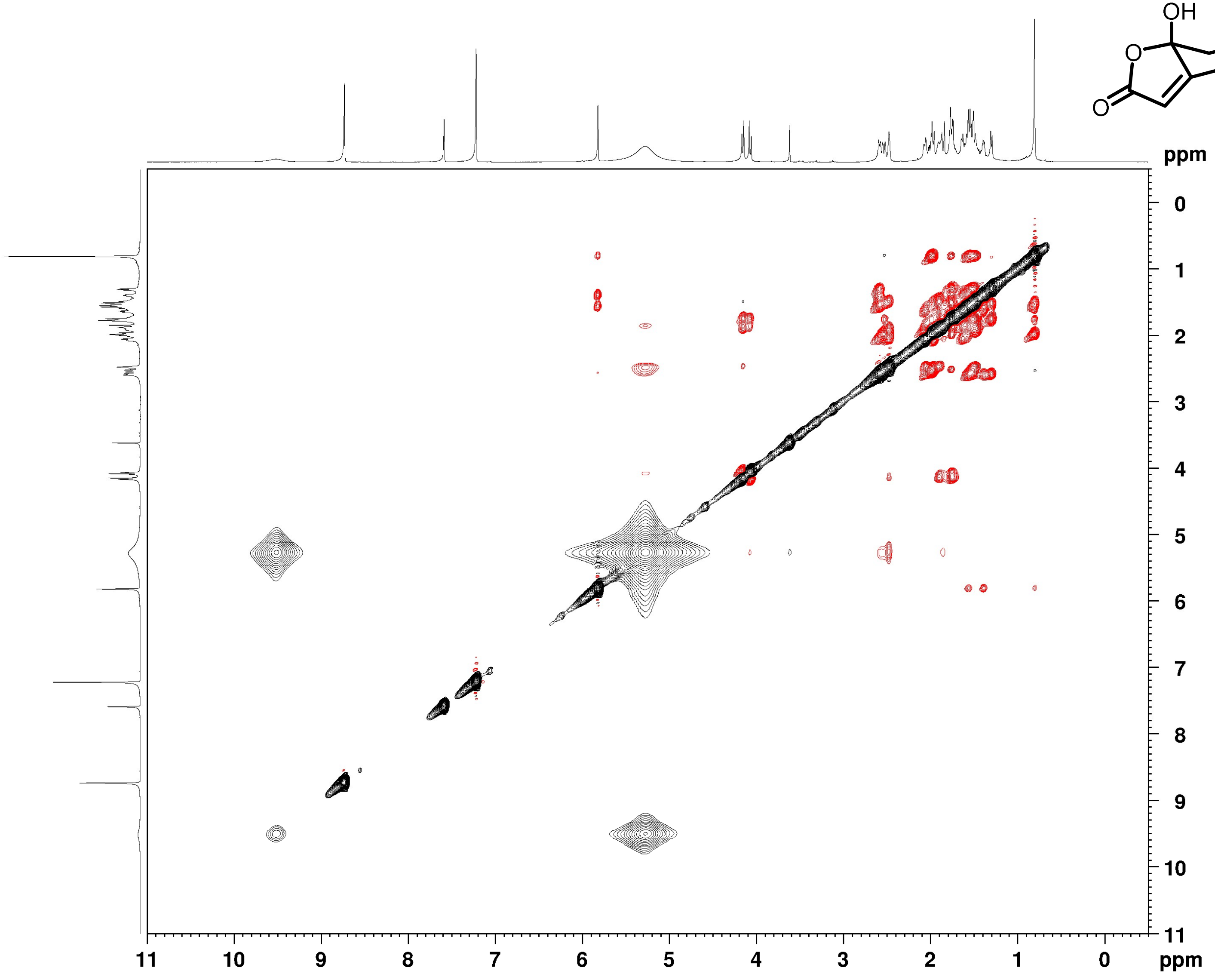


```
Current Data Parameters
NAME            jan12ynsH1
EXPNO                    3
PROCNO                   1

F2 - Acquisition Parameters
Date_             20260115
Time                  1.58 h
INSTRUM              spect
PROBHD     Z113652_0120 (
PULPROG          noesygpph
TD                    2048
SOLVENT                Pyr
NS                       6
DS                      16
SWH               6172.839 Hz
FIDRES            6.028163 Hz
AQ               0.1658880 sec
RG                    45.2
DW                  81.000 usec
DE                   10.00 usec
TE                   298.2 K
D0              0.00006521 sec
D1              1.99180806 sec
D8              0.80000001 sec
D16             0.00020000 sec
IN0             0.00016200 sec
TDav                     1
SFO1           499.8724921 MHz
NUC1                    1H
P1                   12.40 usec
P2                   24.80 usec
PLW1           27.00000000 W
GPNAM[1]        SMSQ10.100
GPZ1                 40.00 %
P16                1000.00 usec

F1 - Acquisition parameters
TD                     256
SFO1              499.8725 MHz
FIDRES           48.225307 Hz
SW                  12.349 ppm
FnMODE                TPPI

F2 - Processing parameters
SI                    4096
SF             499.8699872 MHz
WDW                     GM
SSB                      0
LB                   -1.00 Hz
GB                   0.005
PC                    1.00

F1 - Processing parameters
SI                    1024
MC2                   TPPI
SF             499.8699869 MHz
WDW                     GM
SSB                      0
LB                   -1.00 Hz
GB                    0.01
```

HSQC of compound 14:

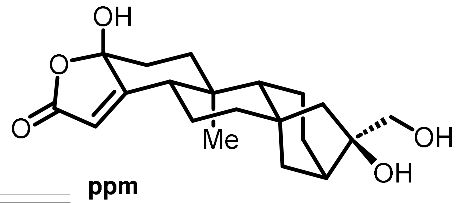

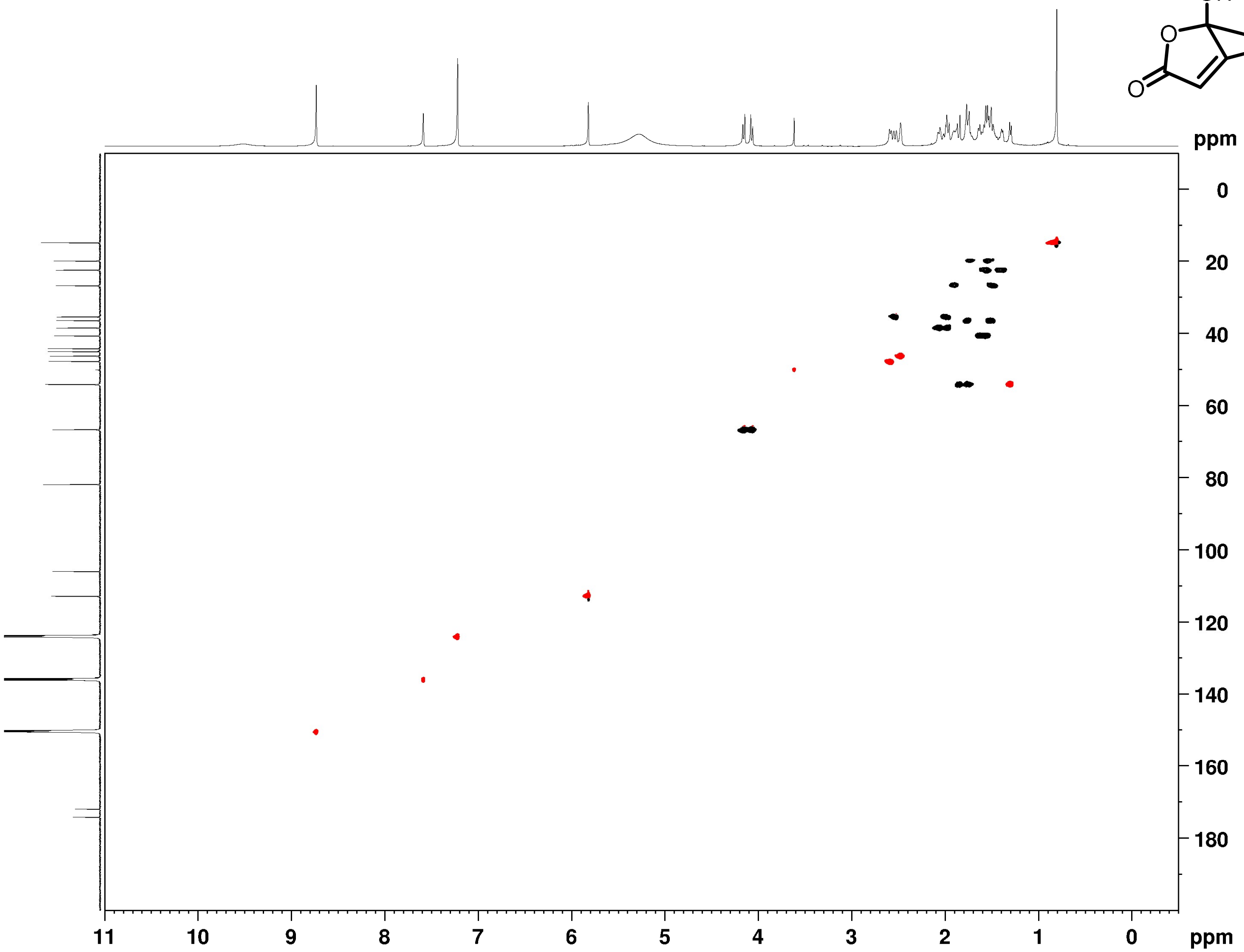


```
Current Data Parameters
NAME           jan12ynsH1
EXPNO                   5
PROCNO                  1

F2 - Acquisition Parameters
Date_            20260115
Time                 6.20 h
INSTRUM             spect
PROBHD   Z113652_0120 (
PULPROG        hsqcedetgp
TD                   2048
SOLVENT               Pyr
NS                      6
DS                     16
SWH              6996.269 Hz
FIDRES           6.832294 Hz
AQ              0.1463637 sec
RG                    203
DW                 71.467 usec
DE                  10.00 usec
TE                  298.2 K
CNST2         145.0000000
D0             0.00000300 sec
D1             2.00000000 sec
D4             0.00172414 sec
D11            0.03000000 sec
D13            0.00000400 sec
D16            0.00020000 sec
D21            0.00344828 sec
IN0            0.00001810 sec
TDav                    1
ZGOPTNS
SFO1          499.8729992 MHz
NUC1                   1H
P1                  12.40 usec
P2                  24.80 usec
P28                     0 usec
PLW1          27.00000000 W
SFO2          125.7049802 MHz
NUC2                  13C
CPDPRG[2             garp
P3                  10.00 usec
P4                  20.00 usec
PCPD2               70.00 usec
PLW2          88.00000000 W
PLW12          1.79589999 W
GPNAM[1]       SMSQ10.100
GPZ1                80.00 %
GPNAM[2]       SMSQ10.100
GPZ2                20.10 %
P16               1000.00 usec

F1 - Acquisition parameters
TD                    256
SFO1              125.705 MHz
FIDRES         215.814911 Hz
SW                219.755 ppm
FnMODE     Echo-Antiecho

F2 - Processing parameters
SI                   4096
SF            499.8699856 MHz
WDW                 QSINE
SSB                     2
LB                      0 Hz
GB                      0
PC                   1.40

F1 - Processing parameters
SI                   1024
MC2        echo-antiecho
SF            125.6923305 MHz
WDW                 QSINE
SSB                     2
LB                      0 Hz
GB                      0
```

HMBC of compound 14:

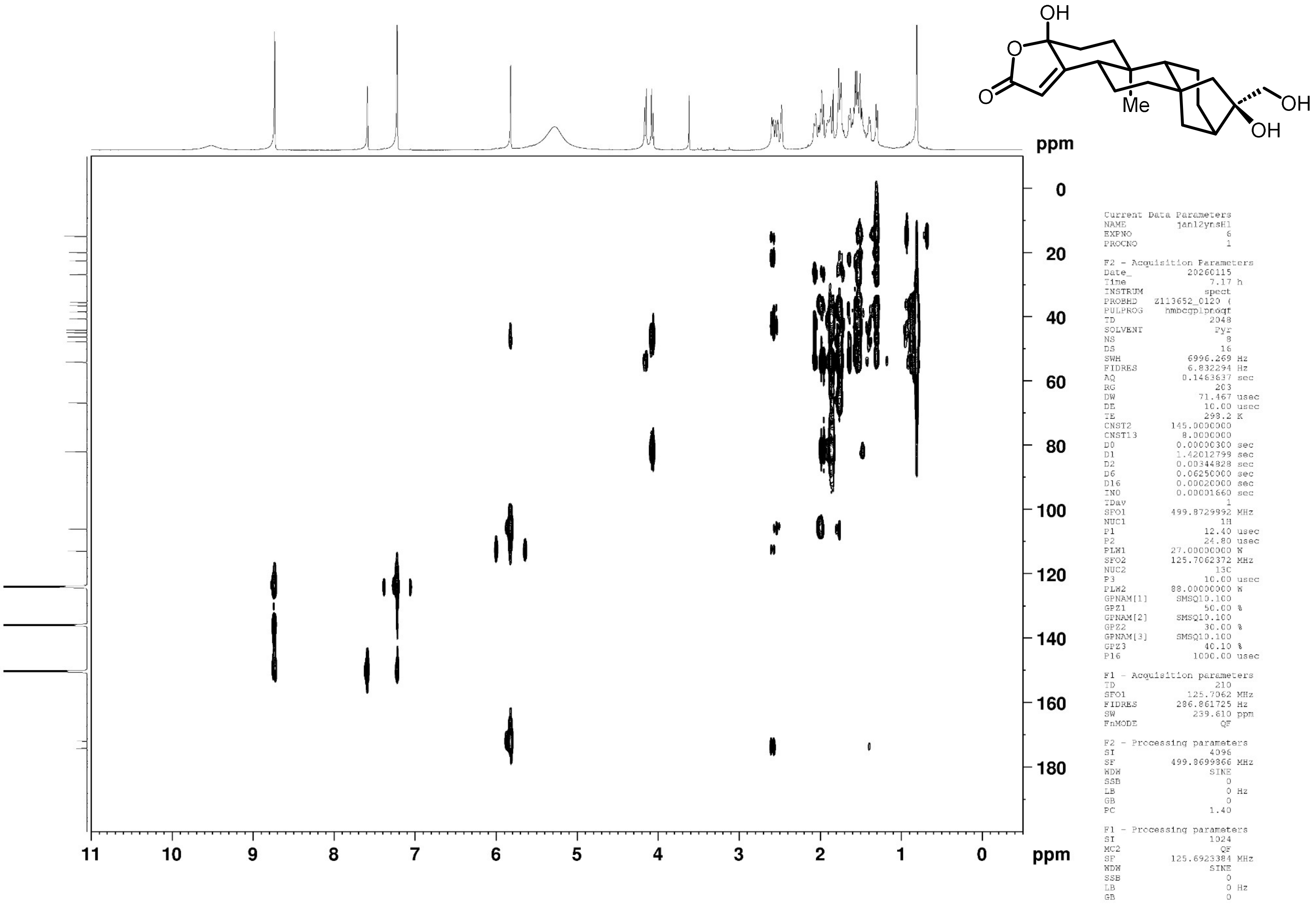

Supplement: Supplementary file 1 [file gg5c00121_si_001.pdf]
